# Neural population models for EEG: From Canonical models to alternative model structures

**DOI:** 10.1371/journal.pcbi.1014222

**Published:** 2026-08-06

**Authors:** Nina Omejc, Sabin Roman, Ljupčo Todorovski, Sašo Džeroski

**Affiliations:** 1 Department of Knowledge Technologies, Jožef Stefan Institute, Ljubljana, Slovenia; 2 Jožef Stefan International Postgraduate School, Ljubljana, Slovenia; 3 Department of Mathematics, Faculty of Mathematics and Physics, University of Ljubljana, Ljubljana, Slovenia; Bournemouth University, UNITED KINGDOM OF GREAT BRITAIN AND NORTHERN IRELAND

## Abstract

Neural population models are widely used to interpret electroencephalography (EEG), yet the relationship between the two remains far less systematically understood as compared with single-neuron models. More fundamentally, it remains unclear whether EEG can support a uniquely plausible population-level mechanism, or whether multiple structurally distinct models can explain the data equally well. To address this question, we combine comparative analysis of canonical model families with grammar-based generation of new candidate architectures. We assemble 17 canonical neural mass and phenomenological models and embed them in a shared structural space. From their common processes, we define a probabilistic grammar over interpretable dynamical components and develop ENEEGMA (Exploring Neural EEG Model Architectures), a Julia-based framework for grammar-based model generation, simulation, and parameter optimization. With this grammar, we generate additional candidate models. We then assess both canonical and generated models by fitting them to EEG independent-component spectra from four datasets for two conditions, i.e., resting state and steady-state visual evoked potentials (SSVEP). Canonical models form six structural clusters. Across conditions, compact low-dimensional polynomial oscillators perform best overall, with generalized Montbrió–Pazó–Roxin, FitzHugh–Nagumo, and Stuart–Landau models offering the best balance of fit quality, stability, and simplicity. Grammar-based exploration further showed that the space of viable EEG node models extends beyond canonical formulations: Even a restricted search over 1,000 generated models produces compact alternatives competitive with nearly all canonical families, with the generated cluster achieving the strongest Bayesian expected rank for SSVEP fits. These findings suggest that EEG spectra constrain classes of plausible population-level dynamical architectures without uniquely determining them and that grammar-based model exploration provides a principled, data-driven framework for EEG-constrained model discovery.

## Author summary

Electroencephalography (EEG) lets us measure brain activity non-invasively, but the signals are indirect, so we rely on mathematical models to explain how neural populations generate them. Many such models exist, yet it is unclear whether standard models cover the full range of plausible explanations for EEG data, or whether several very different models can explain the same signal equally well. In this study, we developed ENEEGMA, an efficient Julia-based tool for comparing established neural population models and automatically generating new candidate models from interpretable building blocks using grammar-based equation discovery. We found that simple low-dimensional oscillator models often matched EEG spectra better than more complex canonical models. We also found that newly generated models could perform nearly as well as, and sometimes better than, established ones, especially for stimulus-driven responses. These results suggest that EEG spectra alone may not be enough to identify a unique underlying neural mechanism. More broadly, our work shows how automated, biologically informed model generation can help to compare, understand, expand, and test the space of candidate neural population models.

## Introduction

In humans, non-invasive recording techniques provide a major source of information about population-level brain dynamics. In particular, electroencephalography (EEG) offers millisecond temporal resolution. However, it provides only indirect and spatially blurred access to the underlying neural population dynamics, which are additionally nonlinear, high-dimensional, and intrinsically noisy [1–3]. Interpreting these signals, therefore, relies on modelling approaches that link neuronal mechanisms to macroscopic EEG observables. These approaches range from classical, biophysically grounded dynamical systems [4–7] to more recent, prediction-oriented data-driven neural networks [8,9]. Here, we focus on biophysically grounded dynamical systems that describe the collective dynamics underlying EEG signals.

**Modelling EEG.** Mathematical models of neural populations span a spectrum [10,11], from detailed single-neuron and spiking-network models [12], through population-density and neural mass formulations [4,13,14], to neural-field models [6,7] and highly reduced phenomenological dynamical systems [15,16]. In this work, we focus on the low-dimensional end of this spectrum, specifically neural mass and phenomenological models, which can be studied within a shared framework of low-dimensional dynamical systems, including ordinary, stochastic, and delay differential equations. This broad low-dimensional class overlaps with what has recently been termed *aggregate neuronal models*, emphasizing macro-level descriptions of collective neural activity [10]. These modelling regimes exhibit a particularly broad diversity of formulations and comparatively fewer standardized reference models than detailed single-neuron and spiking-network models. This combination creates both a practical need and a growing curiosity about the modelling space, highlighting the value of systematic analysis and principled exploration.

Some of the earliest neural mass models were introduced by Lopes da Silva [17], Wilson and Cowan [18], and Jansen and Rit [19]. These models describe the collective dynamics of interacting neuronal populations under the assumption that each population is spatially lumped and internally homogeneous. Their state variables represent population-averaged quantities such as mean membrane potential, mean firing rate, or synaptic current, resulting in low-dimensional dynamical systems. Neural mass models are typically formulated as systems of ordinary differential equations of the form $\dot{x}(t)=f(x(t),u(t))$, often augmented with stochastic fluctuations or transmission delays to capture variability and finite propagation effects [4,14]. Here, *x*(*t*) denotes the vector of popula*t*ion state variables, while *u*(*t*) represents external or afferent inpu*t* to the population. Representative applications include methodological and theoretical studies of seizure, resting-state, and network dynamics [20–27], as well as clinical and translational modeling in disease-related settings [28,29].

Phenomenological models, by contrast, often draw on concepts and mathematical structures originating in physics and dynamical systems theory [11,15]. They have been adapted to capture essential macroscopic dynamical features of neural activity without explicitly modelling underlying biophysical mechanisms. Despite their reduced dimensionality, such models have proven valuable for describing characteristic macroscopic neural dynamics [30], including oscillations, relaxation cycles, synchronization phenomena, and pathological transitions such as seizures [31]. Owing to their balance between mathematical simplicity, computational efficiency, and interpretability at the population level, phenomenological models are used alongside neural mass models in contemporary EEG research [30,32–35].

The literature offers a growing catalogue of candidate neural population models [4,14,36]. Software ecosystems such as The Virtual Brain (TVB) [11,37–39], Brain Dynamics Toolbox [40], PyRates [41,42], and Dynamic Causal Modelling (DCM) [43,44] have made these models widely accessible, providing powerful frameworks for simulating and fitting predefined model structures. Despite the availability of these tools, several conceptual and methodological issues remain insufficiently addressed. In particular, although many neural mass and phenomenological models have been proposed, there is still limited general guidance on how canonical formulations differ in their structural assumptions, dynamical behaviour, and empirical adequacy for specific EEG datasets or scientific questions. Comparative analyses are often model-specific, and a systematic organization of the neural population model space, analogous to that available for single-neuron models, remains largely absent. As a result, it is unclear whether the current collection of canonical models adequately spans the space of plausible population-level dynamics, or whether alternative model structures, consistent with known neurophysiological constraints, could provide equally good or improved descriptions of EEG data.

Addressing these challenges requires a framework that can systematically analyze existing models while also exploring alternative candidate model structures. In practice, such exploration is difficult to carry out manually, given the size and combinatorial diversity of the candidate model space. It therefore depends on a representation of neural population models that is explicit, compositional, and suited to algorithmic search. To this end, we adopt a grammar-based equation discovery approach that probabilistically combines interpretable building blocks derived from canonical neural mass and phenomenological models to generate and formalize candidate neural population model structures.

**Equation discovery via probabilistic grammars.** From a dynamical-systems perspective, modelling an observed signal is an inverse problem: given an observed trajectory 𝐲(t), we assume an underlying dynamical system of the form $\dot{𝐱}(t)=f(𝐱(t),𝐮(t);\theta ),$ where the observed signal is related to the latent state via an observation mapping 𝐲(t)=g(𝐱(t))+ε(t), where ε(t) denotes measurement noise. Classical system identification assumes a known functional form *f* and estimates only the parameters θ. In contrast, equation discovery (also called symbolic regression) infers both the structure of *f* and its parameters from data [45]. In simple terms, equation discovery lets a computer automatically generate and test many candidate equations or model structures, often tens of thousands or more, to identify those that best reproduce the observed data. A variety of methods have been proposed, including early equation-discovery systems such as LAGRAMGE [46,47], sparse regression–based approaches such as SINDy [48,49], probabilistic grammar-based frameworks such as ProGED [50,51], and neural approaches such as DSO [52] and HVAE [53].

In neuroscience, equation-discovery approaches remain relatively unexplored. However, recent work has begun to apply data-driven dynamical reconstruction to large-scale brain modelling. For example, Sip et al. [54] used a data-driven artificial neural network model to infer regional neural-mass-like dynamics and subject- and region-specific parameters from resting-state fMRI while respecting structural connectivity. Similarly, Kashyap et al. [55] applied SINDy-based system identification to learn whole-brain network ODEs from rest and task fMRI data, using the resulting models to separate structured network activity from task-related components. One recent study [56] also used SINDy to recover simulated low-dimensional stochastic decision dynamics in perceptual decision-making tasks, illustrating the promise of such methods in computational neuroscience. To our knowledge, this is the first study to apply grammar-based equation-discovery methods to identify neural population dynamics directly from EEG data. A key challenge in doing so is that searching over arbitrary analytic expressions is difficult: the space of candidate models is vast, and generic algebraic primitives encode little domain-specific knowledge about neural dynamics. To address this challenge, we incorporate domain knowledge by formalizing the search process using a probabilistic grammar, as in ProGED [50,51], that specifies how high-level components of population models are expanded into concrete mathematical forms.

A probabilistic grammar can be thought of as a set of weighted assembly rules: it tells the computer which building blocks can be combined and which combinations are more plausible. This biases the discovery of equations toward simpler, more interpretable, and biologically meaningful structures. Importantly, the interpretation of generated models depends on how the grammar is designed and on which building blocks are included. A grammar restricted to mechanistic neural mass components will tend to generate models with more direct biological interpretations, whereas a grammar that also includes phenomenological dynamical-system components can explore a broader space of macroscopic dynamics, including more abstract oscillator-like descriptions.

Motivated by these considerations, we have developed ENEEGMA (Exploring Neural EEG Model Architectures), a framework that uses a domain-informed, probabilistic grammar to generate, simulate, and evaluate neural population models for EEG data. This framework enables systematic exploration of established formulations as well as a broader model space, including combinations not previously proposed. Using ENEEGMA, we address two central questions: first, how canonical neural mass and phenomenological models compare in terms of structure and empirical performance; and second, whether systematic exploration can reveal alternative, neurophysiologically informed and mathematically interpretable architectures with comparable or improved adequacy for EEG data.

## Materials and methods

We first introduce the established, canonical neural population models and describe the structural and empirical analyses used to compare them. We then detail the construction of the probabilistic grammar, the generation of novel models, and their analysis.

### Canonical neural population models

We collected a set of canonical neural population models from The Virtual Brain (TVB) [37,39], the Brain Dynamics Toolbox [40], PyRates [41], Dynamic Causal Modeling (DCM) [43], and recent comprehensive reviews [36,57]. In Table 1, we list seventeen canonical models that were analysed, compared, and later also used as domain knowledge to construct the grammar.

**Table 1:** Canonical neural population models and their corresponding dynamical grammar components. Each row lists one reference model, its properties, and its coarse-grained structural and dynamical features as represented in the grammar. The columns report: *N*_pop_, the number of interacting neural populations; *N*_var/pop_, the number of state variables per population; *N*_par_, the number of model parameters in the grammar-generated model (which is not necessarily equal to number of parameters in model’s native formulation); *Input/Output processes*, the local transformation used to map inputs to population dynamics and, where applicable, the output operator; *Conn. funcs*, the functional forms used for inter- or intra- population connectivity; and *Conn. motif*, the characteristic connectivity pattern among populations. Model abbreviations are: WC, Wilson–Cowan; ARM, Alpha Rhythm Model; JR, Jansen–Rit; W, Wendling; MDF, Moran–David–Friston; LW, Liley–Wright (reduced); RRWC, Robinson–Rennie–Wright Cortex; RRWT, Robinson–Rennie–Wright Thalamus; WW, Wong–Wang; WWR, reduced Wong–Wang; LB, Larter–Breakspear; HO, Harmonic Oscillator; MPR, Montbrió–Pazó–Roxin; FHN, FitzHugh–Nagumo; VDP, Van der Pol; SL, Stuart–Landau; and DO, Duffing oscillator. Additional abbreviations are: sat. sig. - saturating sigmoid; base. sig. - base sigmoid; mem. integrator - membrane integrator.

Model	*N*_pop_	*N*_var/pop_	*N*_par_	Input/Output processes	Conn. funcs	Conn. motif
WC	2	1	14	exponential / direct	base. sig.	full
ARM	2	2	10	second–order / direct	linear	ring
JR	3	2	25	second–order / direct	sat. sig.	star
W	5	2	57	second–order / direct	sat. sig.; linear	star-tail
MDF	3	2–5	35	second–order / difference	base. sig.; linear	multi-cycle
LW	2	5	41	second–order / mem. integrator	sat. sig.; linear	chain-loop
RRWC	2	4	24	second–order / spatial gradient	sat. sig.; linear	full
RRWT	2	2	16	second–order / direct	sat. sig.; linear	ring
WW	2	1	16	gating kinetics / direct	rectifier	full
WWR	1	1	7	gating kinetics / direct	rectifier	full
LB	1	3	31	voltage–dependent / direct	linear	null
HO	1	2	3	second–order / direct	linear	null
MPR	1	2	9	polynomial / direct	linear	null
FHN	1	2	10	polynomial / direct	linear	null
VDP	1	2	9	polynomial / direct	linear	null
SL	1	2	13	polynomial / direct	linear	null
DO	1	2	9	polynomial / direct	linear	null

Broadly, the surveyed canonical models can be roughly divided into two groups, neural mass models (NMMs) and phenomenological models. In the neural mass models category, we include Wilson–Cowan (WC) [18,58], the Alpha Rhythm Model (ARM) [13,17], Jansen–Rit (JR) [19], Wendling (W) [59], Moran–David–Friston (MDF) [43,57,60,61], Liley–Wright (LW) [62], the Robinson–Rennie–Wright (RRW) thalamo-cortical model [63,64], Wong–Wang (WW) models as implemented in TVB [21,65,66], Larter–Breakspear (LB) [67,68], and the Montbrió–Pazó–Roxin (MPR) model, also referred to as a next-generation neural mass model [69]. Several of these models were not originally formulated as ordinary differential equations, but rather as spatially extended (e.g., neural field models), and are considered here in their reduced, lumped ODE or neural mass formulations. From the set of phenomenological models, we include the harmonic oscillator (HO), FitzHugh–Nagumo (FHN) [70,71], Van der Pol (VDP), Stuart–Landau (SL), and Duffing oscillator (DO) [15]. A mathematical formulation of all models is provided in S1 Appendix.

Importantly, note that the canonical equations define the reference architectures from which the structural components were extracted. For empirical fitting, parameters were optimized independently, and native cross-parameter relationships were not generally imposed unless they were directly encoded in the model construction. We refer to the resulting empirical implementations as generalized canonical models. Their canonical names identify the source equation architecture rather than guaranteeing membership in the original model’s native parameter manifold.

### Structural analysis of canonical models

We performed a structural analysis of canonical neural population models based solely on their mathematical form. Each model is represented as a system of differential equations, from which symbolic expressions of the right-hand sides are extracted. This allowed us to characterize similarities and differences between models at the level of their equations alone, independently of parameter choices and empirical data.

To compare models structurally, we combined two complementary distance measures with equal weights. The first is a syntactic edit distance between symbolic equations [72], which captures fine-grained differences in algebraic structure. It is defined as the minimum number of insertion, deletion, and substitution operations required to transform one symbolic expression into another, and is normalized to the unit interval using bounds estimated from a large reference set of grammar-sampled models. The second measure captures similarity in equation content by comparing the frequencies of operators and functions, irrespective of their ordering. Specifically, it is computed as the cosine distance between feature vectors encoding the counts of operators and functions present in the respective equations [73].

For the canonical models, we computed pairwise structural distances to construct a distance matrix, which served as the basis for clustering. Clustering was performed using agglomerative hierarchical clustering with optimal leaf ordering [74], as implemented in the Julia package Clustering.jl. This procedure enabled the identification of structurally related model families without imposing prior assumptions about model classes.

Additionally, model complexity was quantified as the total number of state equations, reflecting the dimensionality of the underlying dynamical system and serving as a proxy for structural richness and implementation cost.

### Empirical evaluation of canonical models

We performed an empirical evaluation of the generalized canonical neural population models based on their observable dynamics, assessing how well they reproduce the spectral characteristics of EEG data. This approach enabled comparison across models with widely differing internal structure, dimensionality, and parameterization. Fixed points, stability classes, and bifurcation regimes were not imposed a priori as optimization targets.

**EEG data.** Recorded EEG data were obtained from the dataset of Lee et al. [75], accessed via the MOABB framework [76]. This benchmark was selected because it includes both resting-state (RS) recordings and a steady-state visual evoked potential (SSVEP) paradigm, which yield well-characterized and distinct spectral signatures. For each condition, we selected four representative independent components, one from each dataset. For the SSVEP condition, we selected a visual stimulation frequency of 6.67Hz. Detailed preprocessing of the EEG data, including independent component analysis, is described in S2 Appendix.

**Signal representation.** Both empirical data and simulated model outputs were transformed into power spectral density (PSD) estimates using identical spectral estimation settings to ensure comparability. Using Welch’s method, signals were segmented into overlapping windows of length $n_{perseg}=512$ samples with 10% overlap and a Hann window. Spectra were computed over the frequency range 1–45  Hz, averaged across segments, and analyzed on a logarithmic scale (log-power spectrum; hereafter log-PSD). The resulting log-PSD provides a robust, phase-invariant summary of neural dynamics and allows direct comparison between models whose state variables have different interpretations or units.

**External drive.** All models were driven by externally applied input signals constructed to reflect the experimental condition. For RS simulations, models received white noise, providing a common source of background fluctuations across models. Specifically, the raw RS input consisted of independent samples drawn from a standard normal distribution, 𝒩(0,1), generated at the simulation output sampling rate of 256 Hz and linearly interpolated during numerical integration. The same raw input distribution was used for all models, while the sensory-input coupling parameter was fitted separately for each model and dataset to account for differences in state-variable scaling and in how external drive enters the model equations. This avoided imposing a fixed input gain that could introduce model-specific scaling bias. For SSVEP simulations, we used a periodic sinusoid driving component at the stimulation frequency of 6.67 Hz to enable stimulus-locked responses. Note that the input consisted of a pure sinusoid without harmonic components.

**Noise handling.** Noise entered the simulations through three distinct mechanisms. First, as described above, the externally applied input depended on the experimental condition: in RS simulations, all models received a white-noise external drive, whereas in SSVEP simulations, the external drive was a deterministic sinusoid at the 6.67 Hz stimulation frequency. The external input was applied through the sensory input term $S_{i}(t)$ in Eq. (4). Second, some models contained internal stochasticity. For canonical models, stochastic terms were included when they were part of the model formulation. For grammar-generated models, internal stochasticity was determined by the stochasticity production rule in the grammar. When stochasticity was enabled, an SDE diffusion term with amplitude $\sigma_{i}$ was added to the first state variable of the corresponding input-process block. Deterministic models corresponded to $\sigma_{i}=0$. The noise amplitude $\sigma_{i}$ was treated as a model parameter and optimized together with the other free parameters. Third, additive measurement noise, with amplitude derived from the empirical EEG PSD, was added to the simulated observable. For a given model–dataset–condition combination, the same measurement-noise realization and amplitude were retained across the five repeated evaluations.

**Objective function.** Model fitting was guided by a composite spectral objective defined in log-PSD space.

We define a masked mean absolute error (MAE) as


$$\begin{array}{c} ℒ(\theta ;ℳ)=\frac{1}{\left|ℳ\right|}\sum_{f\in ℳ}\left|P_{model}(f;\theta )-P_{data}(f)\right|, \end{array}$$ (1)

where $P_{data}(f)$ and $P_{model}(f;\theta )$ denote the empirical and simulated log-PSD evaluated over the frequency grid ℱ, and ℳ⊆ℱ specifies the frequency bins included in the loss.

The total loss for the resting state is


$$\begin{array}{c} {ℒ}_{RS}(\theta )=w_{p}\;ℒ(\theta ;𝒫)+w_{ap}\;ℒ(\theta ;𝒜), \end{array}$$

where 𝒫 contains detected spectral peaks and 𝒜=ℱ⧵𝒫 captures broadband aperiodic structure. We fixed $w_{p}$ to 1 and treated $w_{ap}$ as a hyperparameter, varying it between 0 and 1.

In SSVEP, for stimulation frequency *f*_0_, let ℋ denote frequency bins around *f*_0_ and its first *H* harmonics. The harmonic contribution is


$$\begin{array}{c} {ℒ}_{harm}(\theta )=\sum_{h=1}^{H}\alpha_{h}ℒ(\theta ;{ℋ}_{h}),\;\alpha_{h}=\lambda^{h-1}. \end{array}$$ (2)

The total SSVEP loss becomes


$$\begin{array}{c} {ℒ}_{SSVEP}(\theta )=w_{p}\;ℒ(\theta ;𝒫)+w_{harm}\;{ℒ}_{harm}(\theta )+w_{ap}\;ℒ(\theta ;𝒜), \end{array}$$

where 𝒜=ℱ⧵(𝒫∪ℋ). We fixed $w_{harm}$ to 1 and treated $w_{p}$ and $w_{ap}$ as coupled hyperparameters, varying together between 0 and 1.

**Parameter estimation.** Parameter estimation was formulated as a model-agnostic optimization problem in which tunable parameters and unknown initial conditions were adjusted to minimize the spectral objective defined above. Optimization was performed using the Covariance Matrix Adaptation Evolution Strategy (CMA-ES) [77], a derivative-free method suited to nonconvex problems, which searched the bounded parameter space for low-loss solutions. For generalized canonical models, the initial parameter values were derived from model-specific literature parameterizations where available.

The optimization imposed finite parameter-wise bounds. However, native cross-parameter equalities or shared parameter relationships were not generally imposed. Thus, parameters that derive from a common parameter in a native formulation could vary independently after translation into the ENEEGMA representation. This parameter-level treatment enabled the same optimization framework to be applied to grammar-generated systems, for which no source-specific native parameterization or associated parameter manifold is available.

To ensure numerical stability and comparable scaling across heterogeneous parameter sets, parameters were internally reparameterized into a shared dimensionless optimization space.

Ordinary differential equations were integrated using an adaptive solver that switches between stiff (Rodas5) and non-stiff (Tsit5) regimes, while stochastic systems were simulated using the Euler–Maruyama method [78]. Each model was simulated for 60 s. The first 2 s were discarded as an initial transient, and spectral evaluation was performed on the remaining 58 s.

To make the fitting and evaluation procedure explicit, the workflow for each model and condition was as follows. Hyperparameter selection was performed on the first dataset. For canonical models, we evaluated 24 hyperparameter configurations, obtained from combinations of four choices:

- spectral-loss weights: for the RS condition, we varied $w_{ap}\in \{0.5,0.75,1.0\}$ while fixing the task-relevant spectral weights to 1; for the SSVEP condition, we fixed the harmonic weight $w_{harm}$ to 1 and varied $w_{p}=w_{ap}\in \{0.5,0.75,1.0\}$;
- parameter-bound width: parameter defaults were initialized from model-specific literature values where available, and the optimization bounds were varied using either a narrower range, from 0.25 to 4 times the default value, or a broader range, from 0.125 to 8 times the default value. For parameters with zero default value, symmetric fallback intervals of [−5,5] and [−10,10] were used, respectively. These were parameter-wise box bounds and did not generally enforce algebraic relationships among parameters inherited from the native source parameterization;
- CMA-ES initial step size: $\sigma_{0}\in \{2,8\}$;
- CMA-ES population size: {100, 150}.

This yielded 24 configurations per model. During hyperparameter tuning, each configuration was evaluated in five independent optimization runs initialized from different starting parameter values, for a total of 120 runs per model. For each hyperparameter configuration, we computed the median integrated absolute error (IAE) across repeats and selected the hyperparameter configuration with the lowest median IAE as the best. The best-performing configuration was then fixed and used to fit the remaining three datasets for each condition.

Using the selected hyperparameter configuration, each model was fitted separately to each dataset and condition. For each model–dataset–condition combination, CMA-ES was run five times from different random initial parameter values, and the best-fitting parameter set from these five optimization runs was selected. Thus, the comparison figures do not show arbitrary parameter samples drawn from the assigned parameter ranges, but rather simulations using the best-fit parameter set.

**Evaluation.** For the final evaluation, the selected best-fit parameter set was held fixed, and the model was simulated five times, each time with newly sampled initial states. In the RS condition, the external white-noise realization was also varied across evaluations. In the SSVEP condition, all evaluations received the same deterministic sinusoidal drive. These evaluations were used to quantify sensitivity to initial-state selection and, where applicable, stochastic forcing at the selected fitted working point. Final results in the comparison figures and tables, therefore, reflect variability across datasets (subjects) and robustness across repeated simulations at the fitted parameter sets, rather than variability across arbitrary parameter values.

Final model comparison and statistical analysis were based on the integrated absolute error (IAE) over the fitting range ℱ,


$$\begin{array}{c} IAE=\int_{ℱ}\left|P_{model}(f)-P_{data}(f)\right|\;df, \end{array}$$

computed numerically by trapezoidal integration. This avoids dependence on the optimization weighting scheme and provides a directly interpretable, distribution-robust measure of spectral discrepancy that does not assume Gaussian residual structure. For SSVEP data, we additionally computed a harmonic-restricted IAE (h-IAE), defined analogously to IAE, but with the integration domain restricted from ℱ to the stimulus-locked harmonic regions ℋ⊂ℱ. This additional metric ensures that model ranking is not primarily driven by the accurate fit of the broadband background aperiodic structure, but rather reflects fidelity to the evoked harmonic response.

To compare models at the group level, performance summaries were aggregated across datasets. Because only four independent datasets per condition were included, we used a nonparametric Bayesian bootstrap [79]. In simple terms, this method repeatedly reweights the observed datasets, recomputes the group-level summaries, and ranks the models, thereby quantifying uncertainty in model comparison without relying on strong distributional assumptions. From these posterior samples, we estimated pairwise posterior probabilities of model superiority, posterior probabilities of being the best-performing model, and Bayesian expected ranks.

### Probabilistic grammar and grammar-derived candidate models

**Fig 1:**
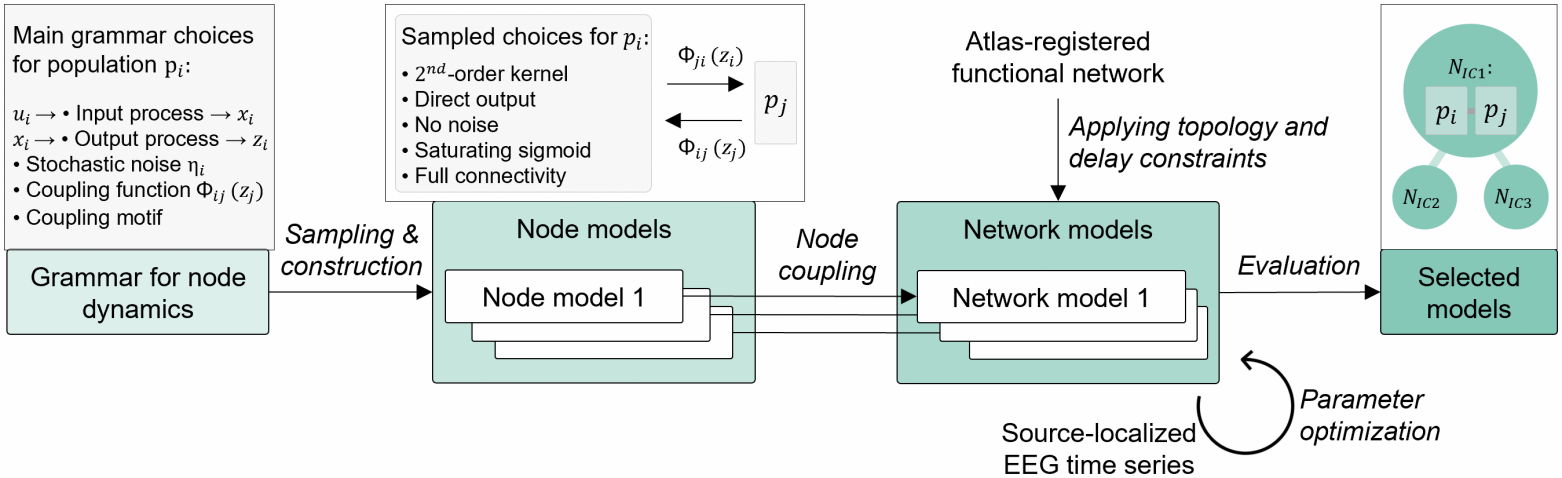
High-level overview of the ENEEGMA pipeline. A probabilistic grammar defines admissible node dynamics in terms of interacting populations, each specified by a selected input process, output process, optional stochasticity, coupling functions, and a connectivity motif. Sampling and construction from this grammar yield candidate node models. These node models are then embedded into atlas-registered functional networks under topology and delay constraints to form network models. The resulting network models are evaluated against source-localized EEG spectral features through parameter optimization, and the best-performing models are selected. Green boxes denote the main components of the ENEEGMA pipeline, italic labels on the arrows indicate the main workflow stages, and gray/white boxes illustrate example components at each stage.

To enable systematic exploration beyond existing neural population models, we developed ENEEGMA. This unified framework integrates grammar-based model generation with the simulation, optimization, and evaluation pipeline described above and graphically in Fig 1. Candidate node-level models are generated by sampling from a probabilistic grammar that encodes construction rules derived from canonical neural population models and specifies how these components are combined into complete systems of differential equations. While ENEEGMA is designed to embed node models within atlas-registered whole-brain networks that incorporate inter-regional coupling and transmission delays, this study restricts the analysis to single-node models. Comprehensive network-level modelling and evaluation are beyond the scope of the present work.

By examining the canonical models, we observe that they can be decomposed into a small number of interacting populations, typically ranging from two to five. We further decompose the dynamics of each population into four main components: (i) *Input processes*, which integrate presynaptic firing rates into internal drive variables; (ii) *Output processes*, which determine how internal quantities are exposed to the rest of the network; (iii) *Connectivity functions*, which describe interactions with other populations within the same node, as well as external connections to other nodes and sensory inputs; and (iv) *Stochastic dynamics* of the model. This modular decomposition is particularly well-suited to neural mass models and provides a natural basis for formalizing model construction. To facilitate comparison, Table 1 presents the structural decomposition of the canonical models and identifies their major dynamical components.

Guided by this decomposition principle, we designed a probabilistic grammar to formalize the construction of node-level neural population models from these components. Table 2 summarizes the core production rules that define node-level model construction. The start symbol Node expands into intermediate nonterminal symbols, which are ultimately instantiated as terminal modelling choices (shown in italics). Expansion proceeds until only terminal symbols remain, yielding a complete structural specification of the node and a blueprint for assembling the corresponding dynamical system. Additionally, each production alternative is associated with a sampling probability. Rather than choosing these probabilities uniformly, we use empirical priors estimated from the relative frequencies of corresponding constructions observed across the set of canonical models. The full grammar, including all production rules, sampling probabilities, and mathematical definitions of each dynamical block, is provided in S3 Appendix.

**Table 2:** Simplified excerpt of the probabilistic grammar for node dynamics. It consists of nonterminal symbols, *terminal symbols* (shown in italics), and production rules describing how the starting nonterminal symbol Node is recursively expanded into terminal symbols corresponding to concrete model components. The grammar defines the hierarchical construction of nodes from populations, their input and output dynamics, intrinsic and extrinsic coupling functions (CF), connectivity motifs (CM), and stochastic noise. Vertical bars (|) indicate distinct alternatives within a rule. Only representative rules and structural alternatives are shown; the full grammar specification, including probabilities, is provided in S3 Appendix.

Node	→	Population CF CM | Population Population CF CM | ...
Population	→	Input processes, Output processes, Coupling flag, Stochasticity
Input processes	→	*Second-order* | *Exponential* | *Linear* | *...*
Output processes	→	*Direct* | *Difference* | *Spatial gradient* | *...*
Coupling function (CF)	→	*Saturating sigmoid* | *Rectifier* | *Linear* | *...*
Connectivity motif (CM)	→	*Full* | *Ring* | *Star* | *...*
Stochasticity	→	*False* | *True*

To illustrate how a canonical neural mass model arises as a specific derivation within the grammar, we consider the classical Wilson–Cowan (WC) model. In this case, the Node symbol is expanded into two interacting populations. For each population, the grammar selects an exponential input process, a direct output process, and no stochasticity. The interaction between populations is specified by a baseline-subtracted sigmoidal coupling function, and the connectivity motif is instantiated as full, allowing reciprocal excitatory–inhibitory interactions. This yields a two-population architecture structurally equivalent to the standard WC model, up to parameter naming and notational conventions. A more detailed derivation of more complex canonical models, such as Jansen–Rit, is provided in S3 Appendix.

More generally, a sampled grammar parse tree induces population-level dynamics of the following form. For each population $i=1,...,n_{pops}$ within a node, the grammar instantiates population-specific dynamics of the form


$$\begin{array}{c} dx_{i}(t)=f_{i}\;\left(x_{i}(t),u_{i}(t);\theta_{i}\right)dt+\sigma_{i}\;dW_{i}(t), \end{array}$$ (3)

where $x_{i}(t)$ denotes the state vector of population *i*, $\theta_{i}$ its parameter set, and $W_{i}(t)$ a standard Wiener process with noise amplitude $\sigma_{i}$ (with $\sigma_{i}=0$ yielding a deterministic system). The functional form of $f_{i}$ is determined by the grammar-selected input and output process blocks. The total input to population *i* is given by


$$\begin{array}{c} u_{i}(t)=\sum_{j=1}^{n_{pops}}\sum_{k}\Phi_{ij}^{\left(k\right)}\;\left(x_{j}(t)\right)+S_{i}(t)+I_{i}^{net}(t), \end{array}$$ (4)

where $\Phi_{ij}^{\left(k\right)}$ are grammar-selected coupling kernels, $S_{i}(t)$ denotes optional external or sensory input, and $I_{i}^{net}(t)$ collects inputs from other nodes. Inter-regional interactions can include finite transmission delays by evaluating coupling terms on delayed states of other nodes, e.g., $x_{j}(t-\tau_{ij})$. In the present work, however, $I_{i}^{net}(t)=0$, as we focus on single-node models. Equations (3)–(4) define the complete population-level dynamics instantiated by a grammar parse tree.

### Structural and empirical analysis of candidate models

Once generated, grammar-derived models were treated analogously to canonical models. Model complexity was quantified as the number of state equations, and structural proximity to canonical families was assessed using the composite structural distance metric already defined above. For a sampled model *M*, its distance to a canonical cluster $C_{k}$ was defined as the minimum structural distance to any model in that cluster,


$$\begin{array}{c} d(M,C_{k})=\min_{M_{i}\in C_{k}}d_{struct}(M,M_{i}). \end{array}$$

Each model was assigned to its closest cluster and characterized jointly by its complexity and minimal cluster distance. This representation places sampled models in a low-dimensional structural space defined by complexity and distance to the nearest canonical family, facilitating comparison, clustering, and identification of structurally novel or canonical-like models.

Empirical analysis followed the same general procedure as for canonical models: generated models were simulated, optimized, and evaluated using identical signal representations, loss functions, and evaluation metrics. The main difference concerned parameter initialization and optimization bounds. For canonical models, parameter defaults and search bounds were taken from model-specific values reported in the literature. For grammar-generated models, such model-specific prior information was not available. We therefore estimated parameter defaults and bounds from the distributions associated with the corresponding grammatical processes in the canonical set. In both groups, the exposed parameters were subsequently optimized independently under the same model-agnostic procedure. To speed up these analyses, experiments were run in parallel on high-performance computing (HPC) clusters. The ENEEGMA source code is publicly available at https://github.com/NinaOmejc/ENEEGMA.

## Results

We first characterized the structural organization and empirical performance of canonical neural population models obtained from the literature, establishing a reference landscape in both structural space and empirical fit. We then evaluated grammar-derived candidate models within this landscape, assessing whether the probabilistic grammar generated structurally novel yet empirically competitive alternatives. Together, these analyses clarified how established models relate to one another and whether the grammar meaningfully expands the space of viable neural population dynamics.

### Structural analysis of canonical models

**Fig 2:**
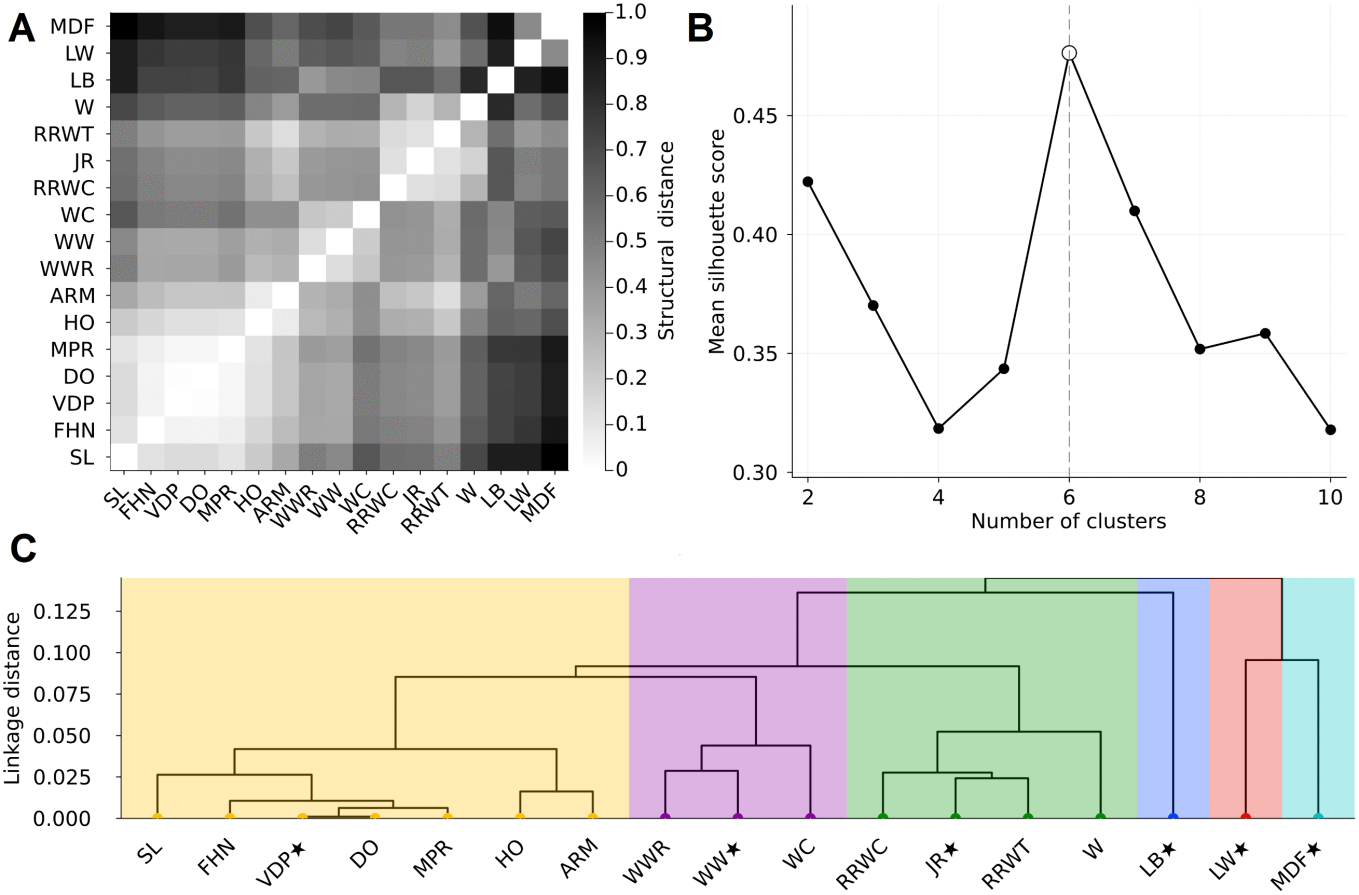
Structural similarity among canonical neural population models. **(A)** Pairwise structural distance matrix, where lower distances (lighter colors) indicate greater structural similarity between models. **(B)** Mean silhouette score as a function of the number of clusters, identifying an optimal partition at *k* = 6. **(C)** Hierarchical clustering based on structural distance separates the models into six structurally coherent clusters (colored regions). The black stars indicate cluster medoids, that is, the most centrally located models within each cluster, defined by the smallest average distance to all other models in the same cluster.

We first examined structural differences among canonical neural population models, quantifying variation in operator and function composition and symbolic structure. Structural difference was measured from the model equations, combining edit distance between symbolic expressions with cosine distance between operator-count vectors. The resulting 17×17 pairwise structural distance matrix is shown in Fig 2A. Hierarchical clustering applied to the matrix produced a stable partition. Evaluation of mean silhouette scores across candidate cluster numbers (Fig 2B) showed a maximum mean silhouette score of 0.48 at *k* = 6, supporting a six-cluster solution. The corresponding dendrogram in Fig 2C delineates six structurally coherent model clusters.

The largest cluster (color-coded in yellow) contains all phenomenological models, together with the compact neural mass models MPR and ARM. It is dominated by low-dimensional, oscillator-like systems (such as VDP, its medoid) with a single population and polynomial nonlinearities, without explicit synaptic convolution or additional output processes. Notably, MPR is a rigorously derived mean-field reduction of spiking neuronal networks, with macroscopic state variables corresponding to population firing rate and mean membrane potential, but it falls within this cluster because its two-dimensional polynomial form is structurally close to low-dimensional oscillator models. ARM also falls within this cluster despite having two populations and second-order dynamics. This likely reflects the fact that, in the present structural analysis, its relatively compact state space, linear coupling, and lack of sigmoidal transfer functions make it more similar to the low-order oscillator group than to more elaborate convolution-based neural mass models such as JR. Thus, this cluster should be interpreted as reflecting equation-level compactness and operator similarity, rather than a single mechanistic model class.

The second-largest cluster (color-coded in green) comprises higher-dimensional architectures organized around second-order synaptic kernels, including JR (medoid), W, and related RRW variants. Despite differences in connectivity motifs, these models share second-order filtered population dynamics and characteristic decay operators.

The third major cluster (color-coded in purple) is less homogeneous than the others and is defined more by a shared architectural template than by a single canonical mechanism. It comprises low-dimensional first-order population models with full coupling motifs and exponential-form transfer nonlinearities, including the baseline-subtracted sigmoid of WC and the relaxed rectifier used in WW/WWR. The medoid of this cluster is WW. Lastly, MDF, LW, and LB form isolated branches. MDF is distinguished by its combination of multiple second-order synaptic subsystems and a difference-based output process, LW by a conductance-based membrane integrator layered on second-order kernels, and LB by voltage-dependent ionic currents and gating variables, placing it structurally apart from convolution-based neural mass formulations.

### Empirical analysis of canonical models

Having characterized the structural organization of canonical models, we next assessed their ability to reproduce the spectral features in EEG data. Note that the exposed parameters were optimized independently within their assigned bounds, and native cross-parameter relationships were not generally enforced. For readability, canonical model abbreviations are retained below as shorthand for these generalized fitted implementations.

**Fig 3:**
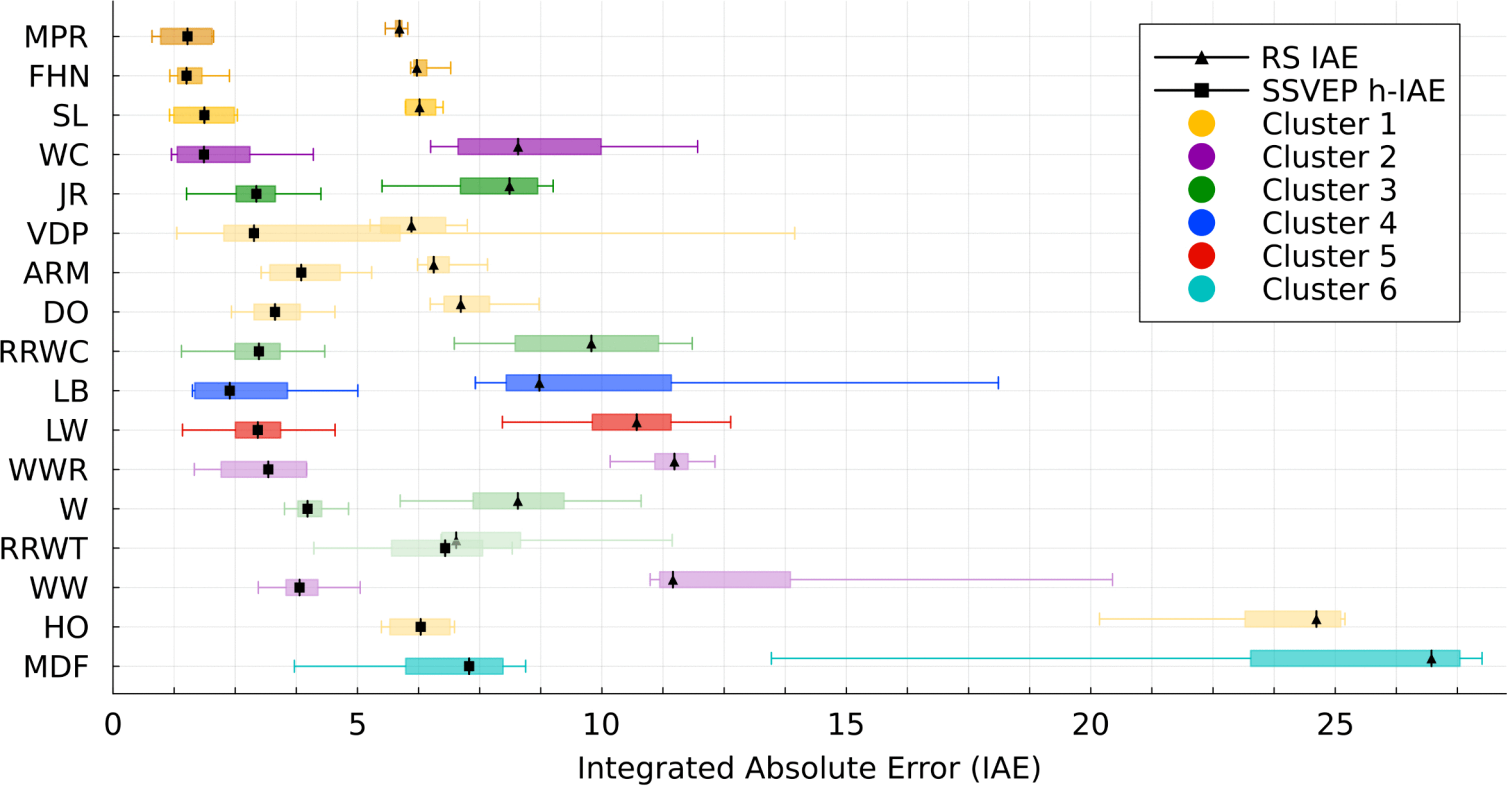
Canonical model comparison across RS and SSVEP conditions. Horizontal boxplots show IAE for RS data (medians marked by triangles) and h-IAE for SSVEP data (medians marked by squares). For each model, dataset, and condition, five evaluations were performed at fixed fitted parameters, varying initial states and, for RS only, external white-noise realizations. SSVEP used the same deterministic sinusoidal drive. Models are ordered by their Bayesian expected rank, averaged across conditions. Canonical abbreviations refer to the model architectures, while the results use generalized fitted implementations.

Fig 3 and Table 3 summarize model performance, measured by the integrated absolute error (IAE) between empirical and simulated log-PSD, across four datasets per condition. For SSVEP (steady-state visual evoked potentials), we report harmonic-restricted IAE (h-IAE) as the primary metric, since full-spectrum IAE can be dominated by broadband structure and thus understate stimulus-locked harmonic accuracy, which was our main quantity of interest. Because h-IAE is computed over a narrower frequency range, its absolute values are smaller and not directly comparable to RS (resting-state) IAE. We therefore base cross-condition comparison primarily on expected ranks.

**Table 3:** Empirical evaluation of canonical models via spectral fitting. Canonical models are ranked by their Bayesian expected rank E[rank], averaged across RS and SSVEP conditions. This rank-based summary is used because median integrated absolute error (IAE) values are condition-specific and therefore not directly comparable on a common scale across the two tasks. The table additionally reports model cluster, average, and condition-specific expected ranks, together with descriptive statistics: dataset-level median IAE for RS, harmonic-restricted IAE (h-IAE) for SSVEP, and relative interquartile range $\left[\Delta Q_{1},\Delta Q_{3}\right]$. Lower values indicate better performance for both IAE and expected rank. Success rate denotes the percentage of runs that produced finite error values. Canonical abbreviations refer to the model architectures, while results use generalized fitted implementations.

Model	Cluster	E[rank]			RS IAE		SSVEP h-IAE		Success
		Avg.	RS	SSVEP	Med.	$\left[\Delta Q_{1},\Delta Q_{3}\right]$	Med.	$\left[\Delta Q_{1},\Delta Q_{3}\right]$	(%)
MPR	1	1.29	1.15	1.43	5.86	[-0.08, 0.05]	1.52	[-0.55, 0.50]	100.00
FHN	1	2.64	3.44	1.84	6.22	[-0.06, 0.20]	1.50	[-0.18, 0.32]	100.00
SL	1	3.10	3.00	3.20	6.27	[-0.28, 0.33]	1.87	[-0.62, 0.61]	100.00
WC	2	6.70	9.63	3.77	8.29	[-1.23, 1.70]	1.86	[-0.55, 0.94]	90.77
JR	3	6.90	7.33	6.46	8.11	[-1.01, 0.58]	2.93	[-0.41, 0.39]	92.73
VDP	1	7.95	2.77	13.13	6.10	[-0.63, 0.70]	2.88	[-0.62, 2.99]	100.00
ARM	1	8.40	4.83	11.97	6.56	[-0.12, 0.32]	3.85	[-0.64, 0.80]	100.00
DO	1	8.41	6.43	10.39	7.11	[-0.34, 0.59]	3.31	[-0.43, 0.52]	100.00
RRWC	3	8.94	10.93	6.95	9.78	[-1.56, 1.37]	2.99	[-0.49, 0.43]	53.33
LB	4	9.77	12.55	6.98	8.72	[-0.68, 2.70]	2.39	[-0.72, 1.18]	100.00
LW	5	10.27	12.55	7.98	10.70	[-0.91, 0.70]	2.96	[-0.46, 0.47]	33.33
WWR	2	10.65	13.64	7.66	11.50	[-0.40, 0.28]	3.18	[-0.97, 0.78]	100.00
W	3	10.66	8.81	12.51	8.28	[-0.92, 0.95]	3.98	[-0.20, 0.29]	87.69
RRWT	3	12.04	8.29	15.79	7.02	[-0.29, 1.32]	6.79	[-1.10, 0.77]	86.67
WW	2	13.07	14.65	11.49	11.50	[-0.27, 2.40]	3.82	[-0.28, 0.38]	100.00
HO	1	15.86	16.39	15.33	24.60	[-1.46, 0.50]	6.29	[-0.63, 0.60]	100.00
MDF	6	16.36	16.61	16.10	27.00	[-3.70, 0.58]	7.29	[-1.30, 0.69]	100.00

Across both conditions, the MPR model achieved the strongest overall performance (E[rank]=1.29) and the highest Bayesian posterior probability of being best (P(best)=84.8% in RS and P(best)=73.6% in the SSVEP task). The second-best model on average, FHN, performed comparably well in the SSVEP condition (E[rank]=1.84) but poorer in the RS condition. The 95% cumulative posterior set was condition-specific: it comprised MPR and VDP in the RS condition, with a cumulative probability of 99.7%, and MPR and FHN in the SSVEP condition, with a cumulative probability of 98.2%. The third-ranked model, SL, with relatively high average rank (E[rank]=3.10), also belongs to the low-dimensional, polynomial, oscillator cluster. Together with MPR and FHN, it exhibits perfect numerical stability (100% success rate), reflecting robust performance across conditions. At a broader level, models in the low-dimensional oscillatory cluster were the strongest performers, particularly in the resting state.

Convolution-based synaptic-kernel architectures (e.g., JR, W, RRWT) occupied an intermediate performance range. Within this cluster, JR performed best, with an expected average rank of E[rank]=6.90. Overall, these models reproduced oscillatory dynamics but exhibited substantially higher RS errors, suggesting a reduced ability to capture broadband spectral structure.

By contrast, several models ranked among the weakest performers. Higher-dimensional neural population models such as LW and MDF showed elevated RS errors and generally weak overall rankings, with average expected ranks of 10.27 and 16.36, respectively. LW was further limited by the lowest numerical stability of any model (33.33% success rate). HO, the structurally simplest linear oscillator, also performed poorly, with high RS error (median IAE = 24.6) and an unfavorable expected rank (E[rank]=15.86).

**Fig 4:**
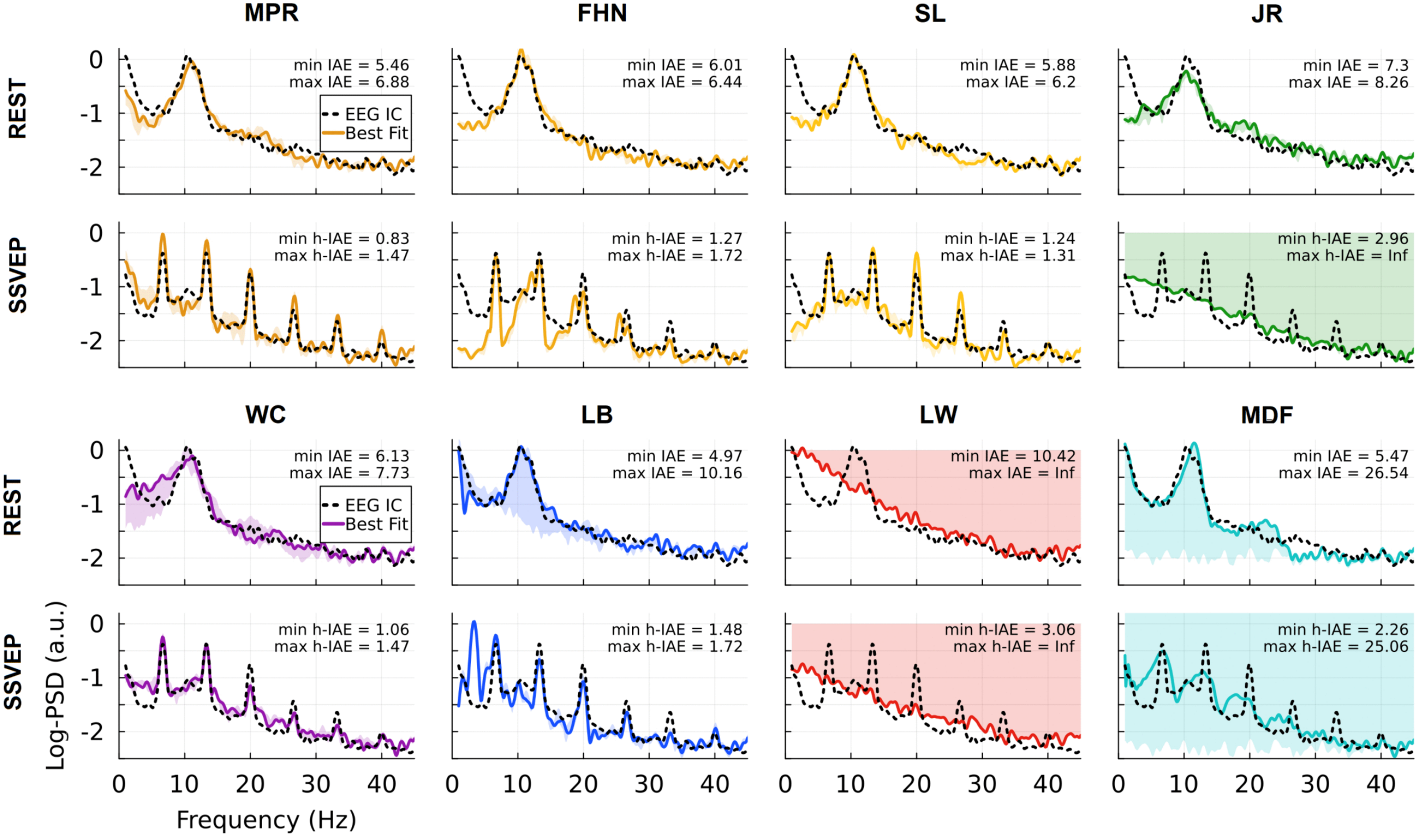
Log-PSD fits of selected canonical models for one representative dataset per condition. Panels show the three top-ranked canonical models together with the best-performing representative from each remaining structural cluster. Rows correspond to RS and SSVEP conditions. Black dashed lines denote empirical log-PSD, colored solid lines the best-fitting simulated spectra, and shaded regions the range (min–max) across five repeated simulations. Five evaluations were performed at fixed fitted parameters, varying initial states and, for RS only, external white-noise realizations. Insets report the minimum and maximum fitting error across repeated simulations (IAE for RS; h-IAE for SSVEP). Infinite maximum errors indicate unsuccessful simulations.

Fig 4 shows illustrative spectral fits for the three top-ranked canonical models together with the best-performing model from each structural cluster, thereby summarizing both the strongest overall performers and representative fits across model families. Results are shown only for the first dataset in each condition, which generally yielded the best fits, because hyperparameters were selected on this dataset and then fixed for the remaining ones. Fits for the other datasets and the remaining canonical models are provided in Figs A and B in S4 Appendix. Visual inspection of Fig 4 indicates that the strongest models are low-dimensional polynomials, particularly MPR, FHN, and SL, which achieved good resting-state fits, capturing both the alpha peak and the overall aperiodic decay with relatively low variability across repeated simulations; the largest residual discrepancies were concentrated mainly at low frequencies. SSVEP fits were generally more demanding, as accurate performance required matching several narrow stimulus-locked harmonic peaks. Even so, MPR, SL, and WC reproduced the harmonic structure well, whereas FHN and LB did so less consistently. By contrast, JR, MDF, and especially LW showed poorer SSVEP fits. Their successful evaluations exhibited greater variability, while some evaluations failed numerically and consequently produced nonfinite error values. These nonfinite values were classified as unsuccessful simulations rather than interpreted as extreme stochastic variability. To complement the PSD-based comparison, we also provide example simulated time series for selected models in Figs B–D in S5 Appendix.

Note that the reason for the presence of multiple peaks in the fitted spectra is a nonlinear two-dimensional dynamical system driven by external input. In the SSVEP condition, the imposed stimulus drive was a pure sinusoid at 6.67 Hz and did not contain harmonics. Harmonic peaks can nevertheless appear in the simulated spectrum because nonlinear dynamics can transform a sinusoidal input into responses containing integer multiples of the driving frequency. Thus, for one-population nonlinear models such as MPR, multiple spectral peaks arise from the interaction between periodic forcing, the fitted operating regime, and nonlinear state dynamics, rather than from multiple population-level oscillators. Fig A in S5 Appendix shows that these harmonic peaks disappear when the sinusoidal forcing is removed.

To characterize the dynamical regime associated with the best-performing canonical architecture, we additionally analyzed the representative generalized MPR fits after setting the external input to zero. In both RS and SSVEP, the autonomous system exhibited a low-activity stable node and a high-activity stable focus separated by a saddle, indicating a bistable fixed-point topology. Relative to Montbrió et al. [69], this topology is analogous to the native MPR bistable regime. However, because the independently fitted parameters do not satisfy the native MPR parameter identities, the fits cannot be mapped to a unique point in the original parameter plane, and the correspondence is therefore only topological. In both conditions, the fitted initial condition converged to the low-activity node. Further details are provided in S9 Appendix.

### Size and complexity of the grammar-generated model space

The grammar defines a rapidly expanding space of admissible node models (Table 4). Although the full grammar can generate a countably infinite number of models, it is still useful to obtain an approximate sense of the scale by treating the *polynomial* input dynamics option as a single admissible choice, rather than enumerating all of the polynomial equations it could generate recursively. Under this simplification, the number of one-population variants follows directly from the available grammar choices for input processes, output processes, coupling terms, and stochasticity, yielding 432 within-population configurations. Combined with the admissible coupling functions and connectivity motif choices at the node level, this gives 5184 distinct one-population models. As the number of populations increases, the number of admissible models grows combinatorially through the combination of additional population blocks, coupling-function assignments, and larger connectivity-motif sets, reaching $1.96\times 10^{17}$ variants for *N* = 5.

**Table 4:** Combinatorial counts of node-model variants generated by the grammar as a function of the number of populations. Because the full grammar can generate a countably infinite number of models, the counts shown here are approximate and are obtained by treating the polynomial input dynamics option as a single admissible choice rather than recursively expanding all polynomial equations it could generate.

Number of populations	Within-population configurations	Connectivity motifs	Coupling-function combinations	Total
1	432	2	6	5184
2	209,952	12	12	$3.02\times 10^{7}$
3	$9.07\times 10^{7}$	107	24	$2.33\times 10^{11}$
4	$4.41\times 10^{10}$	107	48	$2.26\times 10^{14}$
5	$1.90\times 10^{13}$	107	96	$1.96\times 10^{17}$

**Fig 5:**
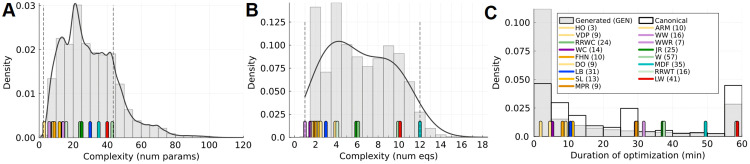
Structural complexity and optimization time for generated and canonical models. Histograms together with density curves show the complexity of one million models sampled from the probabilistic grammar, quantified either by the number of model parameters (Panel **A**) or by the number of state equations (Panel **B**). Color-coded ticks along the horizontal axis indicate the exact values for the canonical models listed in the legend, and dashed vertical lines mark the minimum and maximum values attained by the canonical set. A small horizontal jitter was added to the colored ticks in panel B for visual clarity. **C:** Optimization duration for a subset of 1000 generated models from the low-dimensional oscillator-like region of the grammar-generated model space (GEN; gray histogram), compared with canonical models (white outlined histogram). In panel C, colored ticks indicate the median optimization duration of each canonical model, whereas the outlined histogram summarizes the full distribution across canonical models. Note that for practical computational reasons, the duration of each optimization run was limited to 60 minutes. Numbers in parentheses in the legend denote the number of parameters in each canonical model.

We additionally characterized one million grammar-generated models in terms of structural complexity. Fig 5 shows that the complexity of most sampled models lies within the range covered by the canonical models, as indicated by the dashed vertical lines, especially when the number of state equations quantifies complexity. When quantified by the number of free parameters, the generated models also showed substantial overlap with the canonical range: approximately 81.6% of the one million sampled models had parameter counts within the range spanned by the canonical models. The generated distribution nevertheless had a right tail extending toward more parameter-rich models, reflecting the flexibility of the grammar and its ability to generate structures beyond the parameter-count range represented by the canonical models.

**Fig 6:**
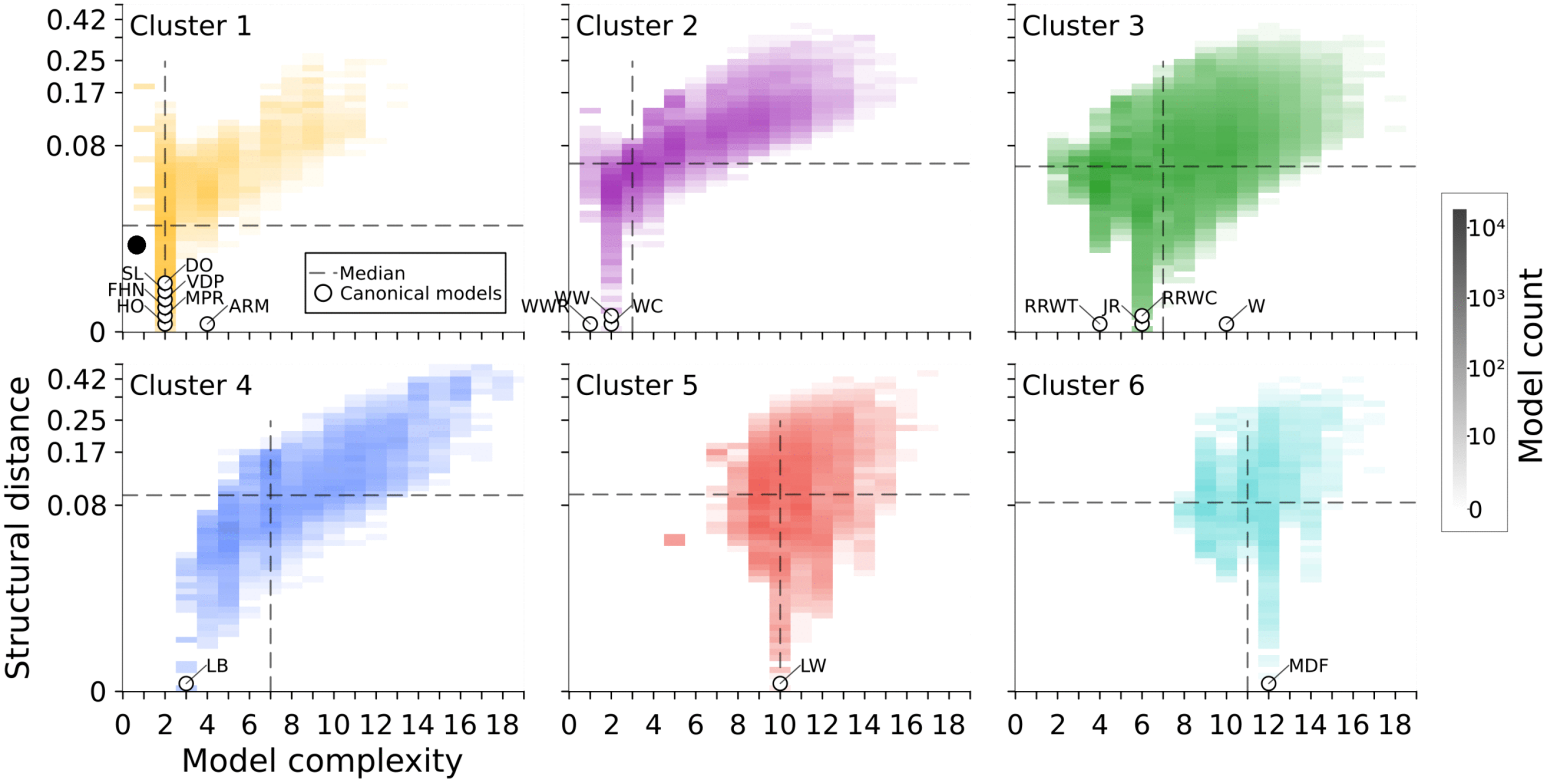
Grammar-sampled models in structural complexity–distance space. Each panel corresponds to one canonical model cluster. Generated models are assigned to clusters based on minimal structural distance to any canonical model within that cluster. The y-axis shows the structural distance to the nearest canonical model within the assigned cluster, and color intensity reflects model density. Dashed vertical and horizontal lines denote the median model complexity and median structural distance, respectively. White circles mark canonical models; their structural distance is zero and is shown with vertical jitter for visibility. The filled black circle indicates the sampled region for empirical evaluation, namely the lower-left quadrant of the best-performing Cluster 1 (yellow).

This broader tail may raise concerns about overfitting if parameter-rich models are fitted without further constraints. However, ENEEGMA makes model complexity explicit, allowing models to be constrained during sampling or filtered after sampling by the number of parameters, number of state equations, structural distance to canonical models, or specific grammar components. In the present study, we used such post-sampling selection by evaluating models located in the low-complexity region of the structural space, which is further explained below (Fig 6).

To assess computational cost, we separately examined optimization times for a subset of 1000 generated models drawn from the low-dimensional oscillator-like region of the grammar-generated model space. Their generally short runtimes are consistent with the fact that the optimized generated subset was restricted to relatively low-dimensional oscillator models. By contrast, the canonical models spanned a broader range of structural complexity, which was reflected in a wider distribution of optimization times, including substantially longer runs.

### Structural analysis of grammar-generated models

In Fig 6, we visualize the structural diversity of grammar-generated models. Each sampled model is assigned to one of the six canonical clusters based on its minimal structural distance to any model within that cluster. The models are embedded in a two-dimensional space defined by structural complexity (number of state equations) and structural distance to the nearest canonical model within the assigned cluster. This representation makes the relationship between generated and canonical models explicit: generated models close to the origin of a panel can be interpreted as canonical-like variants, whereas models farther away represent increasingly distinct equation structures within the same broad structural family. As an additional validation of the grammar construction, we observed that some sampled models had zero structural distance to a canonical model, corresponding to exact recipe matches. In total, the grammar recovered seven canonical models when sampling one million models from the grammar: HO, RRWT, RRWC, JR, WWR, ARM, and LB. This confirms that the grammar can recover established formulations as special cases, rather than only producing novel combinations.

To aid interpretation, we divided the complexity–distance space into four quadrants using the median structural complexity and median structural distance within each panel (dashed lines in Fig 6). For example, the upper-right quadrant contains highly complex and structurally distant models, which tend to be algebraically intricate and less tractable. In contrast, the lower-left quadrant contains simple models that remain close to canonical forms, making them a natural starting point for further evaluation because of their balance of interpretability and continuity with established formulations. Guided by this partition, we did not attempt to evaluate the full combinatorial space of grammar-generated models. Instead, we restricted sampling to the lower-left quadrant of the low-dimensional oscillator-like cluster (color-coded in yellow), which showed the strongest empirical performance among the canonical model clusters. From this region, we empirically evaluated 1,000 models. This focused evaluation provides a preliminary exploration of the grammar-defined model space and serves as a proof of concept for the proposed framework.

### Functional analysis of grammar-generated models

Most of the evaluated 1,000 grammar-generated models produced numerically valid simulations, with success rates exceeding 75% in the RS condition and 95% in the SSVEP condition.

Table 5 summarizes the results at the cluster level. In Panel A, which compares the best-performing model within each cluster, the generated cluster, with its best model G1, ranked second overall (E[rank]=1.67), behind canonical low-dimensional Cluster 1, with the MPR model (E[rank]=1.51). This difference was driven by resting-state performance, where canonical Cluster 1 performed best, whereas the generated cluster achieved the strongest SSVEP fits (E[rank]=1.0).

**Table 5:** Cluster-level empirical performance of canonical and grammar-derived models. Panel A reports the best-performing model within each cluster, whereas Panel B reports the median performance across models within each cluster. Cluster groups are ranked according to their Bayesian expected rank (E[rank]), averaged across RS and SSVEP conditions, as well as across datasets. The table reports average and condition-specific Bayesian expected ranks, together with descriptive statistics: dataset-level median IAE (or h-IAE) and relative interquartile range $\left[\Delta Q_{1},\Delta Q_{3}\right]$. Lower values indicate better performance for both error and rank. The top-performing cluster for each condition is bolded.

Cluster	E[Rank]			RS IAE		SSVEP h-IAE	
	Avg.	RS	SSVEP	Med.	$\left[\Delta Q_{1},\Delta Q_{3}\right]$	Med.	$\left[\Delta Q_{1},\Delta Q_{3}\right]$
*Panel A. Best model in cluster*							
**Cluster 1 (MPR)**	**1.51**	**1.03**	2.00	**5.71**	**[-0.23, 0.20]**	1.09	[-0.12, 0.29]
**Generated (G1)**	1.67	2.34	**1.00**	6.83	[-0.34, 0.03]	**0.68**	**[-0.12, 0.11]**
Cluster 3 (JR)	3.55	2.63	4.46	6.30	[-0.52, 1.00]	2.93	[-0.44, 0.39]
Cluster 2 (WC)	3.65	4.29	3.00	8.29	[-1.23, 1.60]	1.86	[-0.55, 0.66]
Cluster 4 (LB)	5.15	5.33	4.98	8.72	[-0.68, 2.70]	2.39	[-0.72, 1.18]
Cluster 5 (LW)	5.47	5.38	5.56	10.71	[-0.91, 0.70]	2.96	[-0.46, 0.47]
Cluster 6 (MDF)	7.00	7.00	7.00	26.97	[-3.70, 0.58]	7.29	[-1.30, 0.69]
*Panel B. Cluster median*							
**Generated**	**1.73**	2.46	**1.00**	8.46	[-0.89, 0.88]	**1.18**	**[-0.08, 0.14]**
**Cluster 1**	1.75	**1.00**	2.50	**6.44**	**[-0.19, 0.21]**	2.82	[-0.63, 0.38]
Cluster 4	4.19	4.85	3.54	8.72	[-0.68, 2.70]	2.39	[-0.72, 1.18]
Cluster 3	4.23	2.56	5.90	8.06	[-0.74, 1.14]	3.34	[-0.16, 0.33]
Cluster 5	4.32	4.82	3.81	10.71	[-0.91, 0.70]	2.96	[-0.46, 0.47]
Cluster 2	4.78	5.30	4.26	11.12	[-0.33, 0.23]	3.15	[-0.94, 0.77]
Cluster 6	7.00	7.00	7.00	26.97	[-3.70, 0.58]	7.29	[-1.30, 0.69]

Panel B shows cluster-median performance and therefore reflects typical performance across models within each cluster. Here, the generated cluster ranked first overall (E[rank]=1.73), narrowly ahead of canonical Cluster 1 (E[rank]=1.75). Again, the generated cluster performed best in SSVEP, while the canonical Cluster 1 retained the advantage in RS. Together, these results suggest that the generated low-dimensional models are particularly effective at reproducing stimulus-locked harmonic structure, whereas canonical Cluster 1 remains stronger for resting-state spectra.

**Fig 7:**
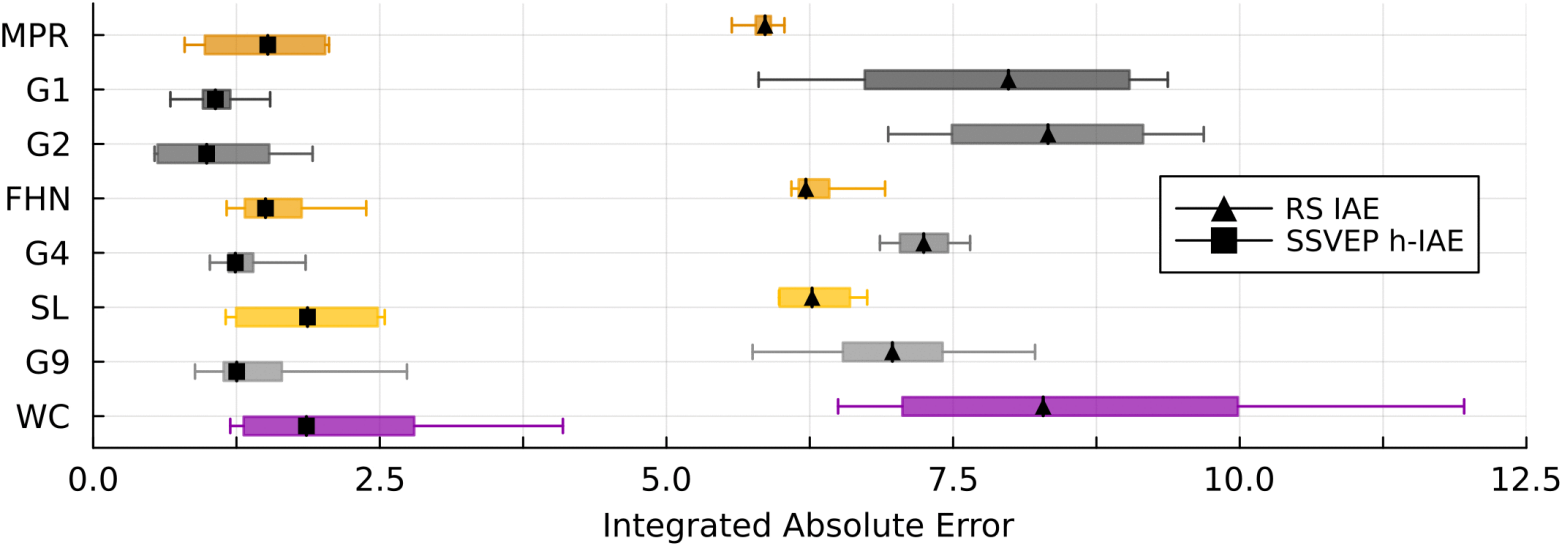
Empirical performance of four of the top-performing generated models. Horizontal boxplots show IAE for RS data (medians marked by triangles) and h-IAE for SSVEP data (medians marked by squares). For each model, dataset, and condition, five evaluations were performed at fixed fitted parameters, varying initial states and, for RS only, external white-noise realizations. SSVEP used the same deterministic sinusoidal drive. Canonical models are included for reference. Models are ordered by their Bayesian expected rank, averaged across conditions. Although only a subset is shown here, rank computation included 20 generated models and 17 canonical models.

**Fig 8:**
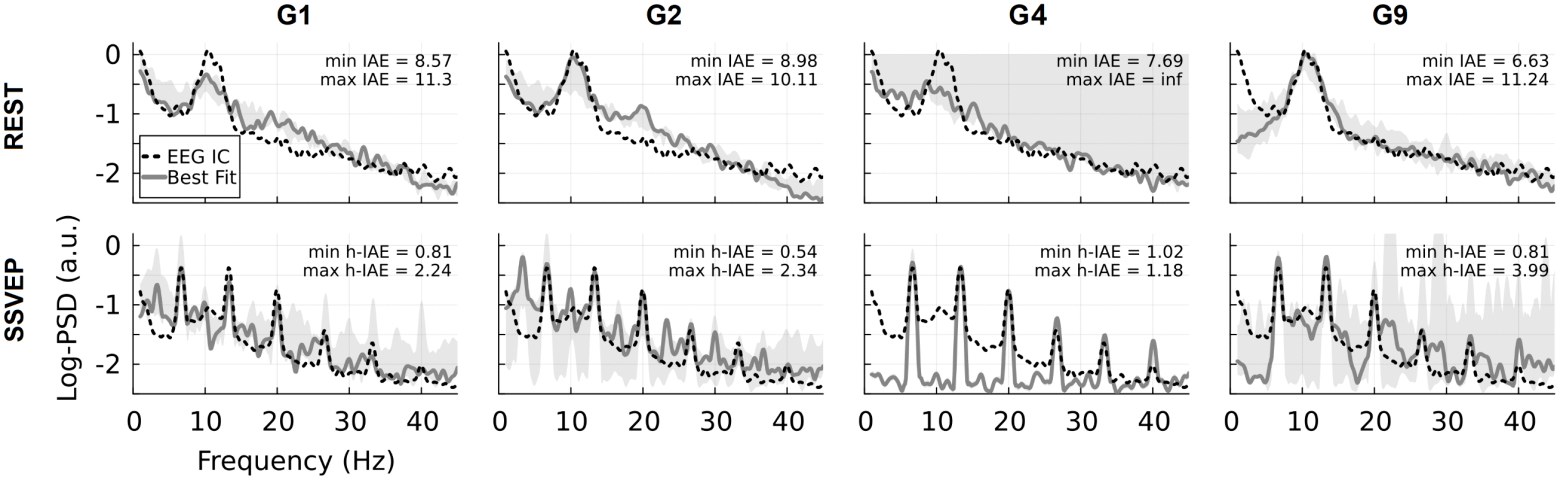
Log-PSD fits of best grammar-generated models. Columns show four representative top-performing generated models (G1, G2, G4, and G9), out of the 1,000 evaluated models in the GEN cluster, and rows correspond to RS and SSVEP conditions. The fits are shown for one representative dataset for each condition. Black dashed curves denote empirical log-PSD, solid gray curves the best-fitting simulated spectra, and shaded regions indicate the range (min–max) across five repeated simulations. Five evaluations were performed at fixed fitted parameters, varying initial states and, for RS only, external white-noise realizations. Insets report the minimum and maximum fitting error across repeated simulations (IAE for RS; h-IAE for SSVEP). Because the SSVEP comparison was at the end based on harmonic-restricted error, close agreement is expected primarily at the stimulus-locked harmonic peaks rather than across the full broadband spectrum.

Figs 7 and 8 together summarize the empirical performance of four top-performing generated models (G1, G2, G4, and G9). In comparison with canonical reference models (MPR, FHN, SL, and WC), the generated models generally had higher RS error than the best canonical low-dimensional models, although their RS performance overlapped with that of WC. In SSVEP, however, all four generated models achieved lower median h-IAE than MPR and substantially outperformed the remaining canonical references, with comparatively tight distributions. The representative log-PSD fits clarify this pattern: in the RS condition, the generated models generally reproduced the dominant alpha peak and more faithfully captured the low-frequency part of the spectrum. However, the alpha peak was often less pronounced than in the empirical data, and residual mismatches remained. In the SSVEP condition, by contrast, they reproduced the stimulus-locked harmonic peaks well, consistent with their low h-IAE values, while mismatches were more evident in broadband spectral regions between and beyond the harmonics. This pattern is expected because optimization was performed using the composite spectral loss, whereas final SSVEP comparison emphasized harmonic-restricted error (h-IAE), which specifically rewards fidelity to the evoked harmonic structure rather than to the full broadband spectrum. Together, these results show that compact grammar-generated models can produce physiologically plausible spectra and are particularly effective and robust in capturing stimulus-locked harmonic structure, while offering more limited gains for resting-state spectral fitting.

The selected generated models all belonged to a common class of compact two-dimensional polynomial systems with direct external drive and low-order nonlinear state interactions. The best model G1 is given by


$$\begin{array}{cc} {\dot{x}}_{11} & =s_{1}\left(c_{11}+c_{13}x_{11}x_{12}+c_{15}\;I(t)\right),\\ {\dot{x}}_{12} & =s_{1}\left(c_{12}+c_{14}x_{11}^{2}+c_{16}\;I(t)\right). \end{array}$$ (5) (6)

Here, *x*_11_(*t*) and *x*_12_(*t*) denote the two state variables, I(t) is the external input, $c_{ij}$ are fitted parameters controlling the constant, coupling, nonlinear, and input terms, and *s*_1_ is a global scaling parameter that sets the overall time scale of the dynamics. This system combines direct input to both state variables with bilinear coupling in ${\dot{x}}_{11}$ and quadratic self-feedback in ${\dot{x}}_{12}$, yielding a compact polynomial oscillator architecture that remains structurally simple while being flexible enough to reproduce the observed spectra. G1 is structurally reminiscent of MPR, the best-performing canonical model, because both are compact two-dimensional polynomial systems with low-order quadratic interactions. Both systems contain a constant term and a bilinear interaction in the first equation, and a constant, external input, and quadratic state dependence in the second equation. However, G1 lacks the full quadratic structure of MPR, in particular, the additional $x_{12}^{2}(t)$ term. The similarity nevertheless suggests that compact quadratic planar dynamics may be particularly effective for the present spectral-fitting task, but does not imply that G1 recovers the MPR mechanism. Complete equations and fitted parameter values are provided in S6 Appendix. We additionally compared the autonomous dynamics of G1 with those of the generalized MPR model. Whereas the representative MPR fits exhibited two stable positive equilibria separated by a saddle, G1 exhibited one stable and one unstable focus in both conditions, with the fitted initial condition converging to the stable focus. The remaining selected models, such as G4 and G9, can be understood as richer variants of the same general class, with additional higher-order and more asymmetric polynomial interactions. Further details on dynamical analysis are provided in S9 Appendix.

## Discussion

In this study, we explored the space of neural population models with two main aims. First, we compared canonical models in terms of both structural organization and the empirical performance of their generalized fitted implementations on single-node EEG spectra. Second, we asked how distinctive these canonical formulations are within the broader model space, and whether systematic grammar-based exploration can reveal alternative node architectures with comparable or improved empirical fit.

To address these two questions, we first organized 17 canonical models within a common structural space derived from their symbolic equation structure and overall complexity. We then evaluated their empirical performance on EEG spectra from four datasets under two experimental conditions: resting state (RS) and steady-state visual evoked potentials (SSVEP). Furthermore, using canonical models as a basis, we constructed a probabilistic grammar, generated one million new candidate models, selected a subset of 1,000 models from a specific region of the structural space associated with the best-performing canonical model cluster, and assessed their performance on real EEG data. All analyses were performed within ENEEGMA, our custom Julia-based framework for grammar-based model construction and analysis. The main findings are discussed below.

**Among the canonical models, low-dimensional polynomial oscillators combine structural simplicity with the best single-node EEG spectral fits.** The structural analysis showed that our 17 canonical models group into 6 clusters. In particular, 1) low-dimensional polynomial oscillators (VDP, SL, FHN, DO, MPR, ARM, HO) formed the largest cluster, distinct from 2) second-order synaptic-kernel neural mass models (JR, W, RRWT, RRWC), 3) low-dimensional population models with full coupling motifs and exponential-form transfer nonlinearities (WW, WWR, WC), and three additional single-model clusters: 4) LB, 5) LW, and 6) MDF.

Low-dimensional oscillator models, especially MPR, FHN, and SL, achieved the strongest overall performance across both RS and SSVEP conditions. This is not entirely surprising, given the long use of compact oscillator models and related low-dimensional dynamical systems in electrophysiological brain modelling (e.g., [32,34,80–82]), together with the fact that many salient EEG features are organized around rhythmic spectral components [1,83]. Importantly, these models should not be understood as purely arbitrary phenomenological oscillators. The native MPR equations constitute a rigorous mean-field reduction of a heterogeneous network of quadratic integrate-and-fire neurons [69], whereas FHN is a simplified two-dimensional model of neuronal excitability and spike generation, commonly viewed as a relaxation-oscillator reduction of Hodgkin–Huxley-type dynamics [70]. The MPR implementation fitted here was generalized, as its independently optimized parameters did not satisfy the native MPR parameter identities. Nevertheless, the representative RS and SSVEP fits exhibited a bistable topology analogous to the native MPR bistable regime. Their advantage in the present setting appears to lie in their compactness: they are sufficiently expressive to generate oscillatory peaks, waveform asymmetry, and harmonics, while remaining stable and relatively easy to optimize. By contrast, more mechanistically detailed neural mass models may encode richer biological structure, but that additional structure is not automatically rewarded in a single-node spectral fitting framework. Additionally, some models, such as WW and WWR, are typically used as local components of larger recurrent circuits or whole-brain systems [84], where oscillatory structure emerges through coupling, feedback, and network interactions rather than from isolated node dynamics alone [65]. Their relatively weak performance here is therefore not a general criticism of the Wong–Wang framework, but rather a consequence of the specific setting studied: single-node spectral fitting without inter-regional coupling. Likewise, the poorer performance of models such as LW, RRWC, and RRWT was not only a matter of fit quality but also of numerical robustness, indicating that stability under repeated optimization and simulation is itself an important practical dimension of model adequacy.

Taken together, these findings show that canonical neural population models differ in both structural organization and empirical performance in the single-node spectral setting. Their symbolic architecture is associated with systematic differences in empirical adequacy, with some model classes proving better matched to EEG spectra in the present setting than others.

**Grammar-based exploration can generate novel models with strong empirical performance.** The ENEEGMA framework makes the node-model structure itself a search variable. Rather than selecting a fixed model family a priori and optimizing only its parameters, as in most current brain modeling toolboxes [38,40,41,43,85], the framework represents node models compositionally in terms of populations, input processes, output processes, coupling functions, connectivity motifs, and stochasticity. This provides a common and extensible language for comparing established neural population models and for generating new candidate systems from the same set of interpretable building blocks. In this sense, the generated models can be interpreted relative to the canonical landscape: some sampled models exactly recover known formulations, while others occupy nearby regions of the same structural families and differ in specific choices of nonlinear, input, and coupling terms. As a validation of this construction, seven canonical models were recovered exactly among the one million sampled models, confirming that the grammar contains established formulations as special cases. Because the grammar is modular, it can also be adapted to specific scientific questions, for example, by adding new dynamical components, restricting particular constructions, or expanding the space of admissible mechanisms.

The empirical results from the grammar-derived models provide a proof of principle for this approach. Although the grammar defines a much larger admissible model space, the present study evaluated only 1,000 samples from one restricted region within the structural space of the best-performing low-dimensional cluster. Even under this limited sampling regime, the generated models were competitive with nearly all canonical families. At the cluster level, the best generated models ranked second overall and achieved the strongest SSVEP fits of all clusters. This is consistent with the preceding section, which showed that compact low-dimensional oscillator-like mechanisms are particularly effective in the present single-node spectral setting. The structural similarity between the best generated model, G1, and MPR also provides an indirect validation of the generated-model search: a data-driven grammar exploration selected a compact quadratic planar structure close to a model that was independently derived from spiking-neuron networks. This correspondence should not be interpreted as mechanistic equivalence, but it suggests that the search recovers equation structures that are consistent with previously established low-dimensional descriptions of population activity.

More broadly, the grammar is useful not only for generating new models, but also for probing which levels of model structure are associated with the best empirical performance. Some of the best-performing generated systems remained structurally close to known oscillator forms. In an additional analysis, shown in Fig A in S7 Appendix, we grouped generated models by population–connectivity configuration, defined by the number of populations and the connectivity motif within a node. The performance distributions were broadly overlapping across configurations, suggesting that coarse circuit architecture alone was not the dominant factor separating generated-model performance. Instead, empirical adequacy likely depended on the model’s full dynamical realization.

We further examined whether fitting robustness was related to model complexity and parameterization scale. The results, shown in Figs A and B in S8 Appendix, indicated that the final fitting error was only weakly related to the number of fitted parameters or state equations, whereas fitting success was more clearly associated with these quantities and, most strongly, with the aggregate scale of literature/default parameter values. Although this aggregate scale is only an exploratory proxy because parameters have different units, its stronger association with fitting success suggests that robustness depends not only on the number of free parameters or state variables, but also heavily on parameter scaling and the sensitivity of the resulting dynamical regimes.

**Spectral fit alone does not uniquely identify model structure.** These findings also raise a broader methodological question: how distinguishable are alternative model structures when several of them can reproduce the same empirical spectra reasonably well? Previous work has largely examined parameter identifiability within fixed neural mass formulations [86–88]. Here, we highlight a complementary issue of model-level distinguishability: even when parameters are well fit, EEG spectra alone may remain insufficient to uniquely discriminate among structurally different population models. This limitation is evident in our results. Although model choice clearly mattered in the single-node setting studied here, several structurally different models still produced reasonably good fits, suggesting that viable EEG node models may be more numerous than is often assumed and that spectral adequacy alone is insufficient to identify a unique underlying mechanism or circuit architecture. The present findings, therefore, indicate that identification from EEG spectra alone may be intrinsically limited, particularly when optimization is based on high-level summary statistics such as the power spectral density. In that sense, a good spectral fit supports a plausible modelling description, but not necessarily a unique biological mechanism.

The additional time-series examples in Figs B–D in S5 Appendix illustrate this limitation from another perspective and point to one way of reducing it – using richer empirical constraints than the PSD alone. While several resting-state PSD fits also qualitatively resembled the empirical time series, SSVEP models could reproduce harmonic peaks in the PSD, while showing poorer time-domain resemblance. This likely reflects the deterministic sinusoidal SSVEP drive used here, without additional stochastic background input, and the harmonic-focused spectral objective. Future work should therefore combine spectral and time-domain constraints and systematically compare alternative evoked-input formulations, for example, deterministic sinusoidal drive alone versus sinusoidal drive combined with background fluctuations. Such multi-observable fitting could also incorporate additional constraints such as functional connectivity, phase coherence, or cross-spectral structure in whole-brain settings, either through normalized weighted loss terms or through Pareto-based multi-objective optimization. Together, these extensions would help reduce, though not fully eliminate, the underdetermination inherent in PSD-only model identification.

**Limitations and future work.** Several limitations of the present study should be acknowledged. First, the empirical canonical-model fits used generalized parameter-level implementations rather than parameterizations constrained to every source model’s native parameter manifold. This was a deliberate methodological choice: it allowed highly heterogeneous canonical and grammar-generated architectures to be fitted within a common optimization framework, including generated systems for which no model-specific native parameterization exists. Literature values informed the canonical parameter defaults and bounds, but exposed parameters were optimized independently, and cross-parameter identities or parameter-sharing constraints were not generally retained unless directly encoded in the implementation. This generalization may increase the empirical flexibility of some canonical architectures and prevent direct mapping of some fitted parameter sets onto their native bifurcation diagrams. Future work should therefore compare generalized and native-parameter-constrained versions of the same architectures, using explicit parameter tying, mechanistic priors, and dynamical-regime constraints to quantify the trade-off between empirical fit and mechanistic fidelity.

Second, the empirical evaluation was based on four datasets and one representative independent component per condition, which limits both statistical power and the breadth of physiological variation. Future work should therefore extend the evaluation to larger cohorts, more components, and more diverse datasets. Nevertheless, the present study still represents a more substantial empirical test than is common in much of the neural population modelling literature, where models are often assessed in simulated settings and only rarely against realistic EEG recordings.

Third, only 1,000 generated models from one restricted region of the grammar-defined space were evaluated. The present search should therefore be regarded as preliminary rather than exhaustive. Moreover, because the best-performing canonical models belonged to the simplest low-dimensional cluster, our targeted sampling focused on that part of the structural space rather than on more biologically detailed neural mass models, where the advantages of the probabilistic grammar may prove even greater. Future work will therefore require broader sampling across the admissible model space.

Fourth, all models were evaluated as isolated single nodes. This was a deliberate simplification, but, as already mentioned, it favors models that can generate relevant spectral structure locally. A natural next step is therefore to extend the framework to coupled multi-node and whole-brain settings, where the interaction between local dynamics and network coupling can be evaluated directly.

Fifth, the grammar currently does not include several important neural population models, including the Epileptor model [35,89], extended next-generation neural mass models with explicit synaptic dynamics [6], the Zerlaut mean-field model [90], low-dimensional density reductions by Pietras and Schwalger [91,92], the Huang–Lin formulation [93], some extensions of the Jansen–Rit model [94], and phase-based dynamical models [95–97]. This also applies to recent excitatory–inhibitory MPR/QIF-based extensions, such as the next-generation neural mass model used by Forrester et al. [98], which includes additional mechanisms such as conductance-based chemical synapses, electrical/gap-junction coupling, and explicit synchrony/order-parameter dynamics. Some of these models are not directly compatible with the current grammar because of fundamental differences in state representation, whereas others would require moderate or substantial extensions, for example, to support partial differential equations, conductance-based synaptic interactions with reversal potentials, or additional order-parameter variables. Expanding the grammar to incorporate these families is therefore an important direction for future work.

Sixth, future work should employ more efficient search strategies, for example, using variational autoencoder-based generative models [53,99]. These approaches use a trained neural generative model to concentrate sampling on more promising regions of model space, rather than relying on random sampling from the grammar.

Finally, we also plan to enable export of generated models in a TVB-compatible format. This would facilitate their use in whole-brain simulations and could improve parameter estimation by leveraging existing TVB optimisation tools [85,100].

**In conclusion,** canonical neural population models were shown to be structurally organized and empirically distinguishable rather than interchangeable in the single-node spectral setting considered here. In particular, compact low-dimensional polynomial oscillator models such as MPR and FHN combined structural simplicity with the strongest overall EEG spectral fits. Second, grammar-based exploration showed that canonical formulations do not exhaust the space of viable EEG node models: even a restricted search recovered alternative architectures with competitive empirical performance. Together, these results establish an EEG-grounded framework for principled, comparative, and data-driven exploration of neural population models.

## Supporting information

S1 Appendix Description of canonical models. (PDF)

S2 Appendix Details of data preprocessing. (PDF)

S3 Appendix Details of the probabilistic grammar. (PDF)

S4 Appendix Additional canonical-model results. (PDF)

S5 Appendix Harmonic generation and simulated time series. (PDF)

S6 Appendix Generated models and fitted parameters. (PDF)

S7 Appendix Performance of generated models across population–connectivity configurations. (PDF)

S8 Appendix Model complexity, parameter scale, and fitting robustness. (PDF)

S9 Appendix Parameterization and dynamical regime of the generalized MPR and G1 models. (PDF)
