# Supplementary material for: Neural population models for EEG: From Canonical models to alternative model structures
Source: PLoS Comput Biol. 2026 Aug 6;22(8):e1014222. doi: 10.1371/journal.pcbi.1014222 (PMC13460749; doi:10.1371/journal.pcbi.1014222)
Supplement: Supplementary file 1 [file pcbi.1014222.s001.pdf]

## Supporting Information

Neural Population Models for EEG: From Canonical Models to Alternative Model Structures

# S1 Appendix: Description of canonical models

Several of the neural mass models considered here originate from spatially extended mean-field or neural field formulations; in this work, we analyze their commonly used spatially lumped reductions.

This appendix summarizes the canonical neural-mass and phenomenological models used to construct the grammar. To provide a consistent representation, all models are rewritten in first-order form with state variables denoted $x_1, x_2, \ldots$. Model-specific parameters are retained in their original notation, while conceptually shared parameters (e.g. time constants, synaptic rates, coupling weights) are unified whenever this can be done without altering the meaning of the model.

**Wilson–Cowan model.**

$$\tau_e \dot{x}_1 = -x_1 + S(w_{ee}x_1 - w_{ei}x_2 + u_e(t)), \tag{1}$$
$$\tau_i \dot{x}_2 = -x_2 + S(w_{ie}x_1 - w_{ii}x_2 + u_i(t)), \tag{2}$$

with baseline subtracting sigmoid function:

$$S(v) = \frac{1}{1 + \exp[-a(v-\theta)]} - \frac{1}{1+\exp(a\theta)}.$$

Here $x_1$ and $x_2$ denote excitatory and inhibitory firing rates, filtered by time constants $\tau_e$ and $\tau_i$. The four coupling parameters $w_{ee}, w_{ei}, w_{ie}, w_{ii}$ encode excitatory–excitatory, excitatory–inhibitory, inhibitory–excitatory, and inhibitory–inhibitory interactions, respectively, while $a$ and $\theta$ define the slope and threshold of the firing-rate nonlinearity. The terms $u_e(t)$ and $u_i(t)$ represent the total afferent input to the node. Refractory saturation factors sometimes used in Wilson–Cowan models, e.g. $(1 - r_e x_1)$ and $(1 - r_i x_2)$, were not included in the present formulation; this corresponds to setting $r_e = r_i = 0$.

**Alpha rhythm model.**

$$\mathrm{d}x_1 = x_2\,\mathrm{d}t, \tag{3}$$
$$\mathrm{d}x_2 = \left[A_e\alpha_e^2\big(u_e(t) - w_{ei}x_3\big) - 2\alpha_e x_2 - \alpha_e^2 x_1\right]\mathrm{d}t + \sigma_e\,\mathrm{d}W_{e,t}, \tag{4}$$
$$\mathrm{d}x_3 = x_4\,\mathrm{d}t, \tag{5}$$
$$\mathrm{d}x_4 = \left[A_i\alpha_i^2\big(u_i(t) + w_{ie}x_1\big) - 2\alpha_i x_4 - \alpha_i^2 x_3\right]\mathrm{d}t + \sigma_i\,\mathrm{d}W_{i,t}, \tag{6}$$

where $W_{e,t}$ and $W_{i,t}$ are independent Wiener processes. The variables $x_1$ and $x_3$ represent relay and reticular membrane potentials, and $x_2, x_4$ are their time derivatives introduced by rewriting the second-order alpha kernels into first-order form. Parameters $\alpha_e, \alpha_i$ determine synaptic rates, $A_e, A_i$ scale PSP amplitudes, and $w_{ie}, w_{ei}$ are excitatory–inhibitory loop weights. The inputs $u_e(t)$ and $u_i(t)$ denote pooled afferent drives to the relay and reticular populations, while $\sigma_e$ and $\sigma_i$ control the amplitude of stochastic perturbations that sustain narrowband alpha oscillations.

**Jansen–Rit model.**

$$\dot{x}_1 = x_2, \tag{7}$$
$$\dot{x}_2 = Aa\,S(x_3 - x_5) - 2a\,x_2 - a^2 x_1, \tag{8}$$

$$\dot{x}_3 = x_4, \tag{9}$$
$$\dot{x}_4 = Aa\big(u(t) + C_2 S(w_e x_1)\big) - 2a\,x_4 - a^2 x_3, \tag{10}$$

$$\dot{x}_5 = x_6, \tag{11}$$
$$\dot{x}_6 = Bb\,S(w_i x_1) - 2b\,x_6 - b^2 x_5, \tag{12}$$

with

$$S(v) = \frac{2e_0}{1 + \exp[r(v_0 - v)]}.$$

State variables $(x_1, x_2)$ correspond to the pyramidal (PYR) population postsynaptic potential and its derivative; $(x_3, x_4)$ correspond to the excitatory interneuron (EIN); and $(x_5, x_6)$ correspond to the inhibitory interneuron (IIN). Parameters $A$ and $B$ set excitatory and inhibitory synaptic gains, while $a$ and $b$ define their characteristic decay rates. The coupling weights $w_e$ and $w_i$ replace the original $C_1$ and $C_3$ and encode the strength of PYR→EIN and PYR→IIN projections, respectively. The remaining gain $C_2$ modulates the EIN→PYR feedback. The external drive $u(t)$ represents the pooled afferent input to the column, such as thalamic excitation or the exogenous sensory input.

**Wendling–Chauvel model.**

$$\begin{aligned}
\dot{x}_1 &= x_2,\\
\dot{x}_2 &= A_1 a_1\,S(x_3 - x_5 - x_7) - 2a_1 x_2 - a_1^2 x_1,\\
\dot{x}_3 &= x_4,\\
\dot{x}_4 &= A_2 a_2\big(p(t) + C_2 S(C_1 x_1)\big) - 2a_2 x_4 - a_2^2 x_3,\\
\dot{x}_5 &= x_6,\\
\dot{x}_6 &= Bb\,C_4 S(C_3 x_1) - 2b x_6 - b^2 x_5,\\
\dot{x}_7 &= x_8,\\
\dot{x}_8 &= Gg\,C_7 S(C_5 x_1 - C_6 x_9) - 2g x_8 - g^2 x_7,\\
\dot{x}_9 &= x_{10},\\
\dot{x}_{10} &= Bb\,S(C_3 x_1) - 2b x_{10} - b^2 x_9.
\end{aligned}$$

with

$$S(v) = \frac{2e_0}{1 + \exp[r(v_0 - v)]}.$$

In this form, $(x_1, x_2)$ represent the pyramidal population (PYR) postsynaptic potential and its derivative; $(x_3, x_4)$ represent the excitatory interneuron (EIN); $(x_5, x_6)$ the slow dendritic inhibitory interneuron; $(x_7, x_8)$ the fast somatic inhibitory interneuron; and $(x_9, x_{10})$ an auxiliary inhibitory branch that modulates the fast inhibitory population. Parameters $A_1, A_2, B, G$ and rates $a_1, a_2, b, g$ define the amplitudes and time constants of the synaptic kernels. The external input $p(t)$ drives the excitatory interneuron population. The coupling constants $C_1, \ldots, C_7$ determine the strengths of the intrinsic connections: pyramidal activity drives the excitatory, slow inhibitory, fast inhibitory, and auxiliary branches, while the auxiliary branch feeds back into the fast inhibitory population through the term $S(C_5 x_1 - C_6 x_9)$. The pyramidal population receives the

combined excitatory and inhibitory feedback through $S(x_3 - x_5 - x_7)$.

**Moran–David–Friston model.**

The equations below follow the reduced first-order formulation summarized and implemented by [1], rather than a direct transcription of the original DCM papers.

$$\dot{x}_1 = x_2, \tag{13}$$
$$\dot{x}_2 = \kappa_e H_e \left[\gamma_1 S(x_9 - a) + I(t)\right] - 2\kappa_e x_2 - \kappa_e^2 x_1, \tag{14}$$
$$\dot{x}_3 = x_4, \tag{15}$$
$$\dot{x}_4 = \kappa_e H_e \gamma_2 S(x_1) - 2\kappa_e x_4 - \kappa_e^2 x_3, \tag{16}$$
$$\dot{x}_5 = x_6, \tag{17}$$
$$\dot{x}_6 = \kappa_i H_i \gamma_4 S(x_{12}) - 2\kappa_i x_6 - \kappa_i^2 x_5, \tag{18}$$
$$\dot{x}_7 = x_8, \tag{19}$$
$$\dot{x}_8 = \kappa_e H_e \gamma_3 S(x_9) - 2\kappa_e x_8 - \kappa_e^2 x_7, \tag{20}$$
$$\dot{x}_{10} = x_{11}, \tag{21}$$
$$\dot{x}_{11} = \kappa_i H_i \gamma_5 S(x_{12}) - 2\kappa_i x_{11} - \kappa_i^2 x_{10}, \tag{22}$$
$$\dot{x}_9 = x_4 - x_6, \tag{23}$$
$$\dot{x}_{12} = x_8 - x_{11}. \tag{24}$$

with

$$S(v) = \frac{1}{1 + \exp[-\rho_1(v - \rho_2)]} - \frac{1}{1 + \exp(\rho_1 \rho_2)}.$$

In this formulation, $(x_1, x_2)$ represents the granular excitatory population, $(x_3, x_4)$ and $(x_5, x_6)$ represent excitatory and inhibitory inputs to the pyramidal population, and $(x_7, x_8)$ and $(x_{10}, x_{11})$ represent excitatory and inhibitory inputs within the inhibitory subpopulation. The variables $x_9$ and $x_{12}$ are integrated difference variables: $x_9$ represents the net pyramidal drive, while $x_{12}$ represents the net inhibitory-population drive. The parameters $H_e$ and $H_i$ are excitatory and inhibitory synaptic gains, while $\kappa_e$ and $\kappa_i$ determine the corresponding synaptic rates. The parameters $\gamma_1$ and $\gamma_5$ define relative coupling strengths among subpopulations, and $a$ is an adaptation or offset term, which was removed from ENEEGMA modeling. The variable $I(t)$ denotes the external or stochastic drive to the granular excitatory population.

**Liley–Wright model.**

$$\dot{x}_1 = x_2, \tag{25}$$
$$\dot{x}_2 = -2\gamma_{ee}x_2 - \gamma_{ee}^2 x_1 + \gamma_{ee}\Gamma_{ee}\, A\big(x_5; N^{\mathrm{b}}_{ee}, p_{ee}, S_{e,\max}, \mu_e, \sigma_e\big), \tag{26}$$
$$\dot{x}_3 = x_4, \tag{27}$$
$$\dot{x}_4 = -2\gamma_{ei}x_4 - \gamma_{ei}^2 x_3 + \gamma_{ei}\Gamma_{ei}\, A\big(x_5; N^{\mathrm{b}}_{ei}, p_{ei}, S_{e,\max}, \mu_e, \sigma_e\big), \tag{28}$$
$$\tau_e\dot{x}_5 = h_{e,r} - x_5 + \Psi(x_5; h_{ee,\mathrm{eq}}, h_{e,r})\, x_1 + \Psi(x_5; h_{ie,\mathrm{eq}}, h_{e,r})\, x_3, \tag{29}$$
$$\dot{x}_6 = x_7, \tag{30}$$
$$\dot{x}_7 = -2\gamma_{ie}x_7 - \gamma_{ie}^2 x_6 + \gamma_{ie}\Gamma_{ie}\, A\big(x_{10}; N^{\mathrm{b}}_{ie}, p_{ie}, S_{i,\max}, \mu_i, \sigma_i\big), \tag{31}$$
$$\dot{x}_8 = x_9, \tag{32}$$
$$\dot{x}_9 = -2\gamma_{ii}x_9 - \gamma_{ii}^2 x_8 + \gamma_{ii}\Gamma_{ii}\, A\big(x_{10}; N^{\mathrm{b}}_{ii}, p_{ii}, S_{i,\max}, \mu_i, \sigma_i\big), \tag{33}$$
$$\tau_i\dot{x}_{10} = h_{i,r} - x_{10} + \Psi(x_{10}; h_{ei,\mathrm{eq}}, h_{i,r})\, x_6 + \Psi(x_{10}; h_{ii,\mathrm{eq}}, h_{i,r})\, x_8, \tag{34}$$
$$\tag{35}$$

All reversal-potential scalings share a unified form

$$\Psi(v; h_{\mathrm{eq}}, h_r) = \frac{h_{\mathrm{eq}} - v}{|h_{\mathrm{eq}} - h_r|},$$

and the effective presynaptic rates are written as

$$A(v; N^{\mathrm{b}}, p, S_{\max}, \mu, \sigma) = N^{\mathrm{b}}\, S(v; S_{\max}, \mu, \sigma) + p,$$

with a shared sigmoidal firing-rate function

$$S(v; S_{\max}, \mu, \sigma) = \frac{S_{\max}}{1 + \exp\big(-\sqrt{2}\,(v-\mu)/\sigma\big)}.$$

We represent the excitatory population by states $(x_1, \dots, x_5)$ and the inhibitory population by $(x_6, \dots, x_{10})$, with the membrane potentials placed last in each population. Here $x_5$ and $x_{10}$ are the excitatory and inhibitory membrane potentials. The variables $(x_1, x_2)$, $(x_3, x_4)$, $(x_6, x_7)$ and $(x_8, x_9)$ form second-order synaptic kernels for the $ee$, $ei$, $ie$, and $ii$ pathways. Parameters $\tau_e, \tau_i$ are membrane time constants, $h_{e,r}, h_{i,r}$ resting potentials, and the pairs $(\Gamma_{\cdot\cdot}, \gamma_{\cdot\cdot})$ set synaptic strength and decay. The terms $N^{\mathrm{b}}_{\cdot\cdot}$ and $p_{\cdot\cdot}$ are background and constant presynaptic rates. This reduced Liley–Wright formulation preserves the conductance-based structure of the original model while remaining low-dimensional.

**Robinson–Rennie–Wright thalamocortical model.**

For the cortical node we use

$$\dot{x}_{c1} = x_{c2}, \tag{36}$$
$$\dot{x}_{c2} = -(\alpha+\beta)x_{c2} + \alpha\beta\big(-x_{c1} + \nu_{ee}x_{c3} + \nu_{ei}S(x_{c1}) + \nu_{es}S(x_{t1}(t-\tau))\big), \tag{37}$$
$$\dot{x}_{c3} = x_{c4}, \tag{38}$$
$$\dot{x}_{c4} = -\gamma x_{c4} - \gamma^2 x_{c3} + \gamma^2 S(x_{c1}), \tag{39}$$
$$\dot{x}_{c5} = x_{c6}, \tag{40}$$
$$\dot{x}_{c6} = -(\alpha+\beta)x_{c6} + \alpha\beta\big(-x_{c5} + \nu_{re}x_{c3}(t-\tau) + \nu_{rs}S(x_{c5})\big), \tag{41}$$

with

$$S(v) = \frac{Q_{\max}}{1 + \exp[-(v-\theta)/\sigma]}.$$

Here $x_{c1}$ denotes the cortical excitatory membrane potential and $x_{c3}$ the cortical excitatory axonal field, with $x_{c2}$ and $x_{c4}$ their first derivatives introduced by the first-order reduction of the original second-order equations. The pair $(x_{c5}, x_{c6})$ represents a cortical inhibitory population and its derivative. The parameters $\alpha, \beta$ set the synaptic rise and decay rates, $\gamma$ governs axonal propagation, and the gains $\nu_{ee}, \nu_{ei}, \nu_{es}$ specify local excitatory coupling and delayed thalamocortical input.

For the thalamic node, we use

$$\dot{x}_{t1} = x_{t2}, \tag{42}$$

$$\dot{x}_{t2} = -(\alpha+\beta)x_{t2} + \alpha\beta\big(-x_{t1} + \nu_{se}x_{c3}(t-\tau) + \nu_{sr}S(x_{t3}) + \nu_{sn}\Phi_n(t)\big), \tag{43}$$

$$\dot{x}_{t3} = x_{t4}, \tag{44}$$

$$\dot{x}_{t4} = -(\alpha+\beta)x_{t4} + \alpha\beta\big(-x_{t3} + \nu_{re}x_{c3}(t-\tau) + \nu_{rs}S(x_{t1})\big), \tag{45}$$

with the same sigmoid $S$.

In the thalamic subsystem, $x_{t1}$ and $x_{t3}$ represent the specific and reticular thalamic membrane potentials, while $x_{t2}$ and $x_{t4}$ are their derivatives. The gains $\nu_{se}$ and $\nu_{re}$ encode delayed corticothalamic input from the cortical excitatory field $x_{c3}$, $\nu_{sr}$ and $\nu_{rs}$ describe intra-thalamic coupling, and $\nu_{sn}\Phi_n(t)$ represents a subcortical (noise) drive. In ENEEGMA we treat these cortical and thalamic subsystems as two separate nodes that together form the full RRW thalamocortical unit.

**Wong–Wang excitatory–inhibitory model.**

These are TVB implementations of the reduced Wong–Wang family, rather than direct transcriptions of the original 2006 decision model [2].

$$\mathrm{d}x_1 = \left(-\frac{x_1}{\tau_1} + (1-x_1)\,\gamma_1\, S(w_p J_N x_1 - J_i x_2 + u_1(t))\right)\mathrm{d}t + \sigma_1\,\mathrm{d}W_{1,t}, \tag{46}$$

$$\mathrm{d}x_2 = \left(-\frac{x_2}{\tau_2} + \gamma_2\, S(J_N x_1 - x_2 + u_2(t))\right)\mathrm{d}t + \sigma_2\,\mathrm{d}W_{2,t}. \tag{47}$$

with relaxed-rectifier transfer

$$S(I) = \frac{aI - b}{1 - \exp[-d(aI-b)]}.$$

Here $x_1$ and $x_2$ denote the excitatory and inhibitory synaptic gating fractions. The parameters $\tau_1$ and $\tau_2$ are synaptic decay time constants, while $\gamma_1$ and $\gamma_2$ denote population gain factors. The parameters $w_p$, $J_N$, and $J_i$ encode recurrent excitatory and inhibitory coupling, and $u_1(t)$ and $u_2(t)$ represent constant external drive to the two populations.

The function $S(\cdot)$ is a parametric approximation of the firing-rate input–output relationship derived from noisy integrate-and-fire neurons in Wong and Wang (2006). The parameters $a$, $b$, and $d$ control the shape of the nonlinearity. The noise amplitudes $\sigma_1$ and $\sigma_2$ scale independent Wiener processes $W_{1,t}$ and $W_{2,t}$; in the deterministic limit, the model is recovered by setting $\sigma_1 = \sigma_2 = 0$.

**Reduced Wong–Wang model.**

This is a one-dimensional reduced Wong–Wang model, from the TVB implementation [3].

$$\mathrm{d}x_1 = \left(-\frac{x_1}{\tau_s} + (1-x_1)\,\gamma\, S\big(wJ_N x_1 + u(t)\big)\right)\mathrm{d}t + \sigma\,\mathrm{d}W_t, \tag{48}$$

with a nonlinear transfer

$$S(I) = \frac{aI - b}{1 - \exp[-d(aI - b)]}.$$

Here, $x_1$ is the excitatory synaptic gating fraction. The term $wJ_N x_1$ represents recurrent excitation, and $u(t)$ denotes pooled external input (which in classical formulations reduces to a constant baseline $I_0$). The parameters $\tau_s$ and $\gamma$ are the synaptic decay constant and gain, respectively. The constants $a, b, d$ determine the shape of the relaxed-rectifier nonlinearity, and $\sigma$ sets the amplitude of additive stochastic fluctuations via the Wiener process $W_t$.

**Larter–Breakspear model.**

$$\dot{x}_1 = -\Big(I_{\mathrm{Ca}}(x_1) + I_{\mathrm{K}}(x_1, x_2) + I_{\mathrm{L}}(x_1) + I_{\mathrm{Na}}(x_1) + I_{\mathrm{IE}}(x_1, x_3)\Big) + a_{ne}\, u(t), \tag{49}$$

$$\dot{x}_2 = \phi\, \frac{m_{\mathrm{K}}(x_1) - x_2}{\tau_{\mathrm{K}}}, \tag{50}$$

$$\dot{x}_3 = b\big(a_{ni}\, u(t) + a_{ei}\, x_1\, Q_V(x_1)\big), \tag{51}$$

where the ionic and synaptic currents are

$$I_{\mathrm{Ca}}(x_1) = \big(g_{\mathrm{Ca}} + (1 - C)\, r_{\mathrm{NMDA}}\, a_{ee}\, Q_V(x_1)\big)\, m_{\mathrm{Ca}}(x_1)\, \big(x_1 - V_{\mathrm{Ca}}\big), \tag{52}$$

$$I_{\mathrm{K}}(x_1, x_2) = g_{\mathrm{K}}\, x_2\, \big(x_1 - V_{\mathrm{K}}\big), \tag{53}$$

$$I_{\mathrm{L}}(x_1) = g_{\mathrm{L}}\, \big(x_1 - V_{\mathrm{L}}\big), \tag{54}$$

$$I_{\mathrm{Na}}(x_1) = \big(g_{\mathrm{Na}}\, m_{\mathrm{Na}}(x_1) + (1 - C)\, a_{ee}\, Q_V(x_1)\big)\, \big(x_1 - V_{\mathrm{Na}}\big), \tag{55}$$

$$I_{\mathrm{IE}}(x_1, x_3) = a_{ie}\, x_3\, Q_Z(x_3). \tag{56}$$

The voltage-dependent activation curves and population firing rates are

$$m_{\mathrm{ion}}(x) = \tfrac{1}{2}\Big(1 + \tanh \frac{x - T_{\mathrm{ion}}}{d_{\mathrm{ion}}}\Big), \qquad \mathrm{ion} \in \{\mathrm{Ca}, \mathrm{Na}, \mathrm{K}\}, \tag{57}$$

$$Q_V(x_1) = Q_{V,\max}\, \tfrac{1}{2}\Big(1 + \tanh \frac{x_1 - V_T}{d_V}\Big), \tag{58}$$

$$Q_Z(x_3) = Q_{Z,\max}\, \tfrac{1}{2}\Big(1 + \tanh \frac{x_3 - Z_T}{d_Z}\Big). \tag{59}$$

Here $x_1$ is the pyramidal membrane potential, $x_2$ the potassium activation variable, and $x_3$ an inhibitory interneuron drive. The parameters $g_{\mathrm{Ca}}, g_{\mathrm{K}}, g_{\mathrm{L}}, g_{\mathrm{Na}}$ are maximal calcium, potassium, leak, and sodium conductances with corresponding reversal potentials $V_{\mathrm{Ca}}, V_{\mathrm{K}}, V_{\mathrm{L}}, V_{\mathrm{Na}}$. The thresholds $T_{\mathrm{Ca}}, T_{\mathrm{Na}}, T_{\mathrm{K}}$ and widths $d_{\mathrm{Ca}}, d_{\mathrm{Na}}, d_{\mathrm{K}}$ shape the ionic activation curves, while $V_T, Z_T$ and $d_V, d_Z$ play the same role for the population firing rates $Q_V$ and $Q_Z$, scaled by $Q_{V,\max}$ and $Q_{Z,\max}$. Synaptic strengths $a_{ee}, a_{ei}, a_{ie}, a_{ne}, a_{ni}$ control excitatory and inhibitory coupling, $r_{\mathrm{NMDA}}$ weights the NMDA contribution, and $C$ sets the AMPA–NMDA mixing. The constants $\tau_{\mathrm{K}}$ and $\phi$ govern potassium kinetics, and $b$ sets the time scale of the inhibitory drive $x_3$. The term $u(t)$ denotes the external input (corresponding to $I_{\mathrm{ext}}$ in the original formulation). This conductance-based mean-field model combines ionic and synaptic nonlinearities and can generate bursting, fast oscillations, and chaotic dynamics.

**Harmonic oscillator model.**

$$\dot{x}_1 = x_2, \tag{60}$$

$$\dot{x}_2 = -2\zeta\omega_0 x_2 - \omega_0^2 x_1 + u(t). \tag{61}$$

The variable $x_1$ represents displacement or membrane potential, with $x_2$ its time derivative. The parameters $\omega_0$ and $\zeta$ are the natural frequency and damping ratio; $u(t)$ is an external drive.

**Montbrió–Pazó–Roxin model.**

The MPR model is an exact mean-field reduction of a heterogeneous network of quadratic integrate-and-fire neurons. Although mechanistically derived, its compact two-dimensional polynomial form is structurally similar to low-dimensional oscillator models.

$$\dot{x}_1 = \frac{\Delta}{\pi} + 2x_1 x_2, \tag{62}$$

$$\dot{x}_2 = x_2^2 + \bar{\eta} + J x_1 - \pi^2 x_1^2 + u(t). \tag{63}$$

Here, $x_1$ denotes the population firing rate and $x_2$ the mean membrane potential of the underlying QIF network. The parameter $\bar{\eta}$ is the center of the distribution of neuronal excitabilities, $\Delta$ is its half-width, and $J$ is the recurrent coupling strength. The term $u(t)$ denotes a common time-dependent external input to the population.

**FitzHugh–Nagumo model.**

$$\dot{x}_1 = x_1 - \frac{1}{3}x_1^3 - x_2 + u(t), \tag{64}$$

$$\dot{x}_2 = \frac{1}{\tau}(a + x_1 - b x_2). \tag{65}$$

The activator variable $x_1$ interacts with a slow recovery variable $x_2$ through parameters $a, b$, with $\tau$ governing the time-scale separation.

**Van der Pol model.**

$$\dot{x}_1 = x_2, \tag{66}$$

$$\dot{x}_2 = \mu(1 - x_1^2)x_2 - x_1. \tag{67}$$

Here $x_1$ is the oscillator position and $x_2$ its velocity; $\mu$ determines the strength of nonlinear damping.

**Stuart–Landau model.**

$$\dot{x}_1 = \mu x_1 - \omega x_2 - (x_1^2 + x_2^2)x_1, \tag{68}$$

$$\dot{x}_2 = \omega x_1 + \mu x_2 - (x_1^2 + x_2^2)x_2. \tag{69}$$

This system is the real two-dimensional form of the Hopf normal form. The parameter $\mu$ controls the stability and amplitude of oscillations, while $\omega$ sets the intrinsic frequency. For $\mu > 0$, trajectories converge to a limit cycle of radius $\sqrt{\mu}$, and the dynamics reduce asymptotically to a phase oscillator. In this sense, the Stuart–Landau model is a state-space extension of simple phase-dynamics models, augmenting phase evolution with an explicit amplitude degree of freedom.

**Duffing Oscillator.**

$$\dot{x}_1 = x_2, \tag{70}$$
$$\dot{x}_2 = -\alpha x_1 - \beta x_1^3 - \delta x_2 + F(t). \tag{71}$$

The system models a nonlinear oscillator with linear stiffness $\alpha$, cubic stiffness $\beta$, damping $\delta$, and forcing $F(t)$. It is a standard benchmark for nonlinear and chaotic dynamics.

## References


1. Bastiaens SP, Momi D, Griffiths JD. A comprehensive investigation of intracortical and corticothalamic models of the alpha rhythm. PLOS Computational Biology. 2025;21(4):e1012926.
2. Deco G, Ponce-Alvarez A, Hagmann P, Romani GL, Mantini D, Corbetta M. How local excitation–inhibition ratio impacts the whole brain dynamics. Journal of Neuroscience. 2014;34(23):7886–7898.
3. Deco G, Ponce-Alvarez A, Mantini D, Romani GL, Hagmann P, Corbetta M. Resting-state functional connectivity emerges from structurally and dynamically shaped slow linear fluctuations. Journal of Neuroscience. 2013;33(27):11239–11252.
