# Supplementary material for: Neural population models for EEG: From Canonical models to alternative model structures
Source: PLoS Comput Biol. 2026 Aug 6;22(8):e1014222. doi: 10.1371/journal.pcbi.1014222 (PMC13460749; doi:10.1371/journal.pcbi.1014222)
Supplement: Supplementary file 2 [file pcbi.1014222.s002.pdf]

Supporting Information

Neural Population Models for EEG: From Canonical Models to Alternative Model Structures

S2 Appendix: Details of data preprocessing

Empirical EEG data were obtained from the dataset of Lee et al. [1], accessed via the MOABB framework [2]. The 64-channel recordings were converted to MATLAB format (R2023a, The MathWorks, USA) and preprocessed in EEGLAB [3]. Continuous data were resampled to 256 Hz, 60 Hz line noise was attenuated, and the signals were band-pass filtered between 0.1 and 48 Hz. Noisy channels were detected automatically using the Clean Rawdata plug-in (version 2.0) with a flatline criterion of 10 s, a channel-correlation criterion of 0.85, and a maximum tolerated bad-time fraction of 0.5, and were then interpolated using spherical interpolation. The data were additionally inspected manually, and bad segments were removed. Recordings were rereferenced to the common average, and independent component analysis was performed using extended Infomax (`runica`). Component source localization was further characterized using DIPFIT with a standard BEM head model, MNI coordinates, and the standard 10–05 electrode layout. Components were classified with ICLabel, and non-brain or artifactual components were rejected semi-automatically using a probability threshold of 0.3 for eye components and 0.5 for the remaining non-brain classes, followed by additional manual inspection. On average, we retained X brain components. For the SSVEP condition, stimulus events were relabeled according to the four stimulation frequencies (5.45, 6.67, 8.57, and 12.0 Hz), and the data were epoched from −500 to 4500 ms relative to stimulus onset, with baseline correction over −200 to 0 ms. To obtain representative neural signals for model fitting, components classified as brain were clustered across datasets using spectral, scalp-topographic, dipole-location, and dipole-moment features. When multiple components from the same dataset fell into the same cluster, the component with the lowest dipole residual variance was retained. From these clustered components, one representative resting-state component and one representative SSVEP component at 6.67 Hz were selected for each of the four analyzed datasets.

References

1. Lee MH, Kwon OY, Kim YJ, Kim HK, Lee YE, Williamson J, et al. EEG dataset and OpenBMI toolbox for three BCI paradigms: An investigation into BCI illiteracy. GigaScience. 2019;8(5):giz002.

2. Aristimunha B, Carrara I, Guetschel P, Sedlar S, Rodrigues P, Sosulski J, et al.. Mother of all BCI Benchmarks; 2025. Available from: `https://github.com/NeuroTechX/moabb`.

3. Delorme A, Makeig S. EEGLAB: an open source toolbox for analysis of single-trial EEG dynamics including independent component analysis. Journal of neuroscience methods. 2004;134(1):9–21.
