# Supplementary material for: Neural population models for EEG: From Canonical models to alternative model structures
Source: PLoS Comput Biol. 2026 Aug 6;22(8):e1014222. doi: 10.1371/journal.pcbi.1014222 (PMC13460749; doi:10.1371/journal.pcbi.1014222)
Supplement: Supplementary file 3 [file pcbi.1014222.s003.pdf]

Supporting Information

Neural Population Models for EEG: From Canonical Models to Alternative Model Structures

S3 Appendix: Details of the probabilistic grammar

We first present the full grammar in Table A, then describe its components, and finally illustrate the model generation process with a detailed example of the Jansen-Rit model. The resulting grammar defines a structured yet flexible generative model of node dynamics, balancing expressiveness with fidelity to established neural-mass formulations. Each terminal symbol in the grammar corresponds to a dynamical building block. Chosen blocks are assembled automatically into population-level and node-level dynamical systems. All equations below use the standard notation $x(t)$ for state variables, $u(t)$ for the pooled presynaptic drive, and θ for parameters.

Table A. Probabilistic grammar for node dynamics. It is composed of nonterminal symbols, *terminal symbols*, and production rules, which describe the evolution of the starting nonterminal symbol Node to the terminal symbols, which represent concrete model choices. Vertical bars (|) indicate distinct alternatives within a rule. Each production alternative is associated with a probability (shown in square brackets), enabling stochastic sampling of node architectures. The grammar defines the hierarchical construction of nodes from populations (Pop1, Pop2), their intrinsic and extrinsic coupling functions (ECF, PCF, CF), input and output processes (InputProcess, OutputProcess), algebraic combinations of variables (S, P, V), and connectivity motifs (CM, Digit). Abbreviations: Pop = population; ECF = extrinsic coupling function; PCF = population coupling function; CF = connectivity function; CM = connectivity motif; S = sum; P = product; V = variable.

Node	→	Pop1 CF SCF CM1				[0.50]
		Pops2 CF SCF{1..2} CM2				[0.28]
		Pop1 Pops2 CF SCF{1..3} CM				[0.06]
		Pops2 Pops2 CF SCF{1..4} CM				[0.06]
		Pops2 Pops2 Pop1 CF SCF{1..5} CM				[0.10]
Pops2	→	Pop1 Pop1	[0.78]	|	Pop2	[0.22]
Pop1	→	InputProcess OutputProcess ECF ECF Stochasticity				[1.00]
Pop2	→	InputProcess InputProcess OutputProcess ECF ECF ECF ECF Stochasticity				[1.00]
InputProcess	→	S S	[0.14]	|	*Second-order*	[0.66]
		Exponential	[0.08]	|	*Gating kinetics*	[0.06]
		Linear	[0.03]	|	*Voltage-dependent*	[0.03]
OutputProcess	→	*Direct readout*	[0.84]	|	*Membrane integrator*	[0.06]
		Difference	[0.06]	|	*Spatial gradient*	[0.04]
SCF	→	*Custom*	[0.89]	|	*Linear*	[0.11]
ECF	→	*Linear*	[0.53]	|	*False*	[0.32]
		Custom	[0.15]			
CF	→	*Saturating sigmoid*	[0.52]	|	*Baseline-subtracted sigmoid*	[0.24]
		Relaxed rectifier	[0.24]			
S	→	P + S	[0.5]	|	P	[0.5]
P	→	V * P	[0.42]	|	V	[0.58]
V	→	*x1*	[0.56]	|	*x2*	[0.44]
CM1	→	*Null*	[0.8]	|	*Full*	[0.2]
CM2	→	*Full*	[0.50]	|	*Ring*	[0.30]
		Digit	[0.20]			
CM	→	*Full*	[0.06]	|	*Ring*	[0.06]
		Star	[0.40]	|	*Hub-tail*	[0.06]
		Star-tail	[0.06]	|	*Star-loop-extended*	[0.15]
		EI-extended	[0.15]	|	*Erdos–Renyi* Digit Digit	[0.06]
Digit	→	*0*	[0.1]	|	*1*	[0.1]
		2	[0.1]	|	*3*	[0.1]
		4	[0.1]	|	*5*	[0.1]
		6	[0.1]	|	*7*	[0.1]
		8	[0.1]	|	*9*	[0.1]
Stochasticity	→	*False*	[0.83]	|	*True*	[0.17]

Input processes

Input process blocks transform a pooled presynaptic firing-rate signal $u(t)$ into low-dimensional internal state variables. In the grammar, each block is an ODE system of the form

$$\dot{\mathbf{x}}(t) = f(\mathbf{x}(t), u(t); \theta), \tag{1}$$

and populations may have one or two such blocks, depending on the grammar parse tree. We consider five explicit input-process families: linear, exponential, gating-kinetics, second-order PSP, and voltage-dependent dynamics, together with one grammar-generated polynomial family. These six families were selected because, across canonical neural mass and phenomenological models (Table 1 in the main manuscript), every population-level synaptic or intrinsic subsystem can be represented or closely approximated by one of these dynamical templates. Grammar-generated polynomial dynamics is the most flexible and provides a broad basis for data-driven discovery. The remaining families provide a compact yet mechanistically interpretable basis that spans classical EEG modelling practice. Below, we list mathematical descriptions of the input process blocks:

Linear dynamics. This block represents the simplest affine input-driven dynamics, with baseline term a and input gain b:

$$\dot{x}(t) = a + b\, u(t). \tag{2}$$

First-order exponential synaptic kernel:

$$\dot{x}(t) = \frac{1}{\tau}\left(u(t) - \kappa\, x(t)\right) \tag{3}$$

The parameters are the effective time constant $\tau > 0$ and the decay factor $\kappa > 0$. It is also called a linear leaky integrator and is the canonical Wilson–Cowan synaptic filter.

First-order gating kinetics.

$$\dot{x}(t) = -\frac{1}{\tau} x(t) + \kappa\,(1 - x(t))\, u(t) \tag{4}$$

It represents a synaptic gating variable that opens proportionally to the input $u(t)$, and relaxes back with time constant $\tau > 0$ and input gain $\kappa > 0$. Used in Wong–Wang synaptic gating models [1–3].

Second-order PSP kernel.

$$\dot{x}_1 = x_2, \tag{5}$$
$$\dot{x}_2 = \omega^2\big(g\, u(t) - x_1\big) - 2\zeta\omega\, x_2, \tag{6}$$

where $\omega > 0$ is the natural frequency, g an input gain, and ζ is the damping ratio. All second-order PSP variants (bi-exponential, α-kernel, and resonant kernels) are represented within this single damped-driven oscillator family. Special cases include when $\zeta = 1$, we get a critically damped α-kernel, and when $\zeta > 1$, we get over-damped bi-exponential kernels. When $\zeta < 1$, we get resonant kernels (harmonic oscillator). For reference, the classical *alpha kernel* with amplitude A and rate $a > 0$ is

$$\dot{x}_1(t) = x_2(t), \tag{7}$$
$$\dot{x}_2(t) = Aa\, u(t) - 2a\, x_2(t) - a^2 x_1(t), \tag{8}$$

whose impulse response is proportional to te^{-at}. This is recovered from the unified form by identifying $g = A$, $\omega = a$, and $\zeta = 1$.

A more general *bi-exponential* kernel, with separate rise and decay time constants $\alpha, \beta > 0$, obeys

$$\dot{x}_1(t) = x_2(t), \tag{9}$$

$$\dot{x}_2(t) = \frac{1}{\alpha\beta}\big(u(t) - x_1(t)\big) - \Big(\frac{1}{\alpha} + \frac{1}{\beta}\Big)x_2(t), \tag{10}$$

which corresponds to a particular choice of (ω, ζ, g) in the unified second-order system (here $g = 1$, and ω, ζ are chosen so that $\omega^2 = 1/(\alpha\beta)$ and $2\zeta\omega = 1/\alpha + 1/\beta$). This unified second-order form covers the kernel dynamics used in JR, ARM, RRW, W, and LW, and also includes the harmonic oscillator as a special mathematical case.

Voltage-dependent conductance-based dynamics. The conductance-based block consists of three state variables: membrane potential $x_1(t)$, potassium gating variable $x_2(t)$, and an auxiliary inhibitory-state variable $x_3(t)$. The dynamics are

$$\dot{x}_1(t) = -\big(I_{\mathrm{Ca}}(t) + I_{\mathrm{K}}(t) + I_{\mathrm{L}}(t) + I_{\mathrm{Na}}(t) + I_{\mathrm{IE}}(t)\big) + I_{\mathrm{ext}}(t), \tag{11}$$

$$\dot{x}_2(t) = \phi\,\frac{m_{\mathrm{K}}(x_1(t)) - x_2(t)}{\tau_{\mathrm{K}}}, \tag{12}$$

$$\dot{x}_3(t) = b\left(a_{ni}\,u(t) + a_{ei}\,x_1(t)\,Q_V(x_1(t))\right), \tag{13}$$

where the ionic and synaptic currents are

$$I_{\mathrm{Ca}}(t) = \Big(g_{\mathrm{Ca}} + (1 - C)\,r_{\mathrm{NMDA}}\,a_{ee}\,Q_V(x_1(t))\Big)\,m_{\mathrm{Ca}}(x_1(t))\,(x_1(t) - V_{\mathrm{Ca}}), \tag{14}$$

$$I_{\mathrm{K}}(t) = g_{\mathrm{K}}\,x_2(t)\,(x_1(t) - V_{\mathrm{K}}), \tag{15}$$

$$I_{\mathrm{L}}(t) = g_{\mathrm{L}}\,(x_1(t) - V_{\mathrm{L}}), \tag{16}$$

$$I_{\mathrm{Na}}(t) = \Big(g_{\mathrm{Na}}\,m_{\mathrm{Na}}(x_1(t)) + (1 - C)\,a_{ee}\,Q_V(x_1(t))\Big)\,(x_1(t) - V_{\mathrm{Na}}), \tag{17}$$

$$I_{\mathrm{IE}}(t) = a_{ie}\,x_3(t)\,Q_Z(x_3(t)), \tag{18}$$

$$I_{\mathrm{ext}}(t) = a_{ne}\,u(t). \tag{19}$$

The voltage-dependent activation functions are

$$m_{\mathrm{ion}}(x) = \frac{1}{2}\left(1 + \tanh\frac{x - T_{\mathrm{ion}}}{d_{\mathrm{ion}}}\right), \qquad \mathrm{ion} \in \{\mathrm{Ca}, \mathrm{Na}, \mathrm{K}\}, \tag{20}$$

and the firing-rate transforms are

$$Q_V(x_1) = Q_{V,\max}\,\frac{1}{2}\left(1 + \tanh\frac{x_1 - V_T}{d_V}\right), \qquad Q_Z(x_3) = Q_{Z,\max}\,\frac{1}{2}\left(1 + \tanh\frac{x_3 - Z_T}{d_Z}\right). \tag{21}$$

Parameters $g_{\mathrm{Ca}}, g_{\mathrm{K}}, g_{\mathrm{L}}, g_{\mathrm{Na}}$ are the maximal calcium, potassium, leak, and sodium conductances, and $V_{\mathrm{Ca}}, V_{\mathrm{K}}, V_{\mathrm{L}}, V_{\mathrm{Na}}$ are the corresponding reversal potentials. The activation thresholds $T_{\mathrm{Ca}}, T_{\mathrm{K}}, T_{\mathrm{Na}}$ and widths $d_{\mathrm{Ca}}, d_{\mathrm{K}}, d_{\mathrm{Na}}$ determine the sigmoidal activation functions $m_{\mathrm{Ca}}, m_{\mathrm{K}}, m_{\mathrm{Na}}$. The firing-rate transforms Q_V and Q_Z are controlled by their maximal amplitudes $Q_{V,\max}$ and $Q_{Z,\max}$, thresholds V_T and Z_T, and widths d_V and d_Z. Synaptic coupling parameters a_{ee}, a_{ei}, a_{ie}, a_{ne}, and a_{ni} govern, respectively, excitatory-to-excitatory, excitatory-to-inhibitory, inhibitory-to-excitatory, external-to-excitatory, and external-to-inhibitory influences. The parameter r_{NMDA} scales the NMDA-mediated component of excitatory input, while C controls the

AMPA–NMDA mixing ratio. Finally, τ_{K} is the potassium gating time constant, ϕ sets the rate of potassium activation, and b determines the time scale of the inhibitory-state variable x_3.

This three-variable conductance-based system corresponds to the dynamics of the Larter–Breakspear model. We do not treat the inhibitory-state variable x_3 as a separate population because, in the Larter–Breakspear formulation, it does not represent an autonomous neuronal subpopulation with its own incoming and outgoing synaptic projections. Instead, it acts as an internal slow modulatory variable contributing to inhibitory feedback through the nonlinear transform $Q_Z(x_3)$. Treating x_1, x_2, and x_3 as components of a single conductance-based population, therefore preserves the original mean-field interpretation and is consistent with common usage of the model in TVB and related literature.

Grammar-generated polynomials. Polynomial input processes are generated by the following recursive subgrammar:

$$\begin{aligned}
\texttt{InputProcess} \rightarrow \quad & S\,S \quad [0.14] \\
S \rightarrow \quad & S + P\,[0.50] \quad | \quad P\,[0.50] \\
P \rightarrow \quad & V * P\,\,[0.42] \quad | \quad V\,[0.58] \\
V \rightarrow \quad & x_1 \qquad [0.56] \quad | \quad x_2[0.44]
\end{aligned}$$

It can construct polynomial expressions on the right-hand side of neural population equations. It produces exactly two expressions, since `InputProcess` expands to two instances of S. The nonterminal S generates sums of multiplicative terms (P), and P produces products of state variables (V). Rule probabilities are given in square brackets and were inferred empirically for terminal symbols.

Notably, the grammar-generated polynomial family is strictly more expressive than the other types. It can reproduce the structure of low-order polynomial oscillators and approximate some specialized dynamics, including second-order kernels. This overlap does not negate the value of keeping explicit kernel families in the grammar, as they encode the canonical PSP structures used throughout neural mass modelling and thereby provide strong mechanistic inductive biases and a constrained, interpretable search space. On the other hand, polynomial dynamics offer a flexible universal expansion that complements rather than replaces these physiologically grounded components. When counting the number of admissible models, we exclude the recursive polynomial subgrammar, which in principle generates a countably infinite family of polynomial input processes.

Output processes

Output processes determine how internal variables of a population are turned into presynaptic signals that are transmitted to other populations or nodes. In ENEEGMA, these are implemented as optional low-dimensional ODE blocks that append additional state variables to the population and mark a subset of them as "output" variables. For a given population, $\mathbf{x}(t)$ collects the variables created by the input process blocks, and $\mathbf{y}(t)$ contains the additional state variables introduced by an output process block. For notational brevity, when a single internal component is referenced without specifying an index, we write it as $x(t)$; otherwise, $x_j(t)$ denotes the jth component of the internal state vector $\mathbf{x}(t)$. When an output process introduces auxiliary state variables, these obey a system of differential equations of the generic form

$$\dot{\mathbf{y}}(t) = g(\mathbf{y}(t), \mathbf{x}(t); \boldsymbol{\phi})\,, \tag{22}$$

where $\boldsymbol{\phi}$ are the parameters. In our grammar, we set four output process blocks:

Direct readout.

The simplest case, *direct readout*, introduces no additional state variables; instead, an existing internal variable is designated as the population's presynaptic output. This behaviour matches the WC, JR, W, MDF, WW, HO, and polynomial model families, which do not employ additional output processes.

Spatial-gradient (second-order) dynamics.

$$\dot{y}_1(t) = y_2(t), \tag{23}$$

$$\dot{y}_2(t) = \gamma^2(-\,y_1(t) + x(t)) - 2\gamma\, y_2(t), \tag{24}$$

where $\gamma > 0$ is a rate parameter and $x(t)$ is a designated internal state variable from the population's input process blocks. This subsystem implements a critically damped second-order filter acting on $x(t)$, providing temporal smoothing and shaping of the outgoing activity. Such construction appears in the RRW cortical model.

Membrane integrator.

$$\tau\, \dot{y}(t) = h_r - y(t) + \sum_j \Psi(y(t), h_r)\; x_j(t), \tag{25}$$

with the reversal-potential transform

$$\Psi(y(t), h_r) = \frac{h_r - y(t)}{|h_r - h_{\mathrm{eq}}|}. \tag{26}$$

Here $\tau > 0$ is a membrane time constant, h_r a resting potential, and $x_j(t)$ are the input signals from the population's internal dynamics. The factor Ψ scales each input according to the difference between the current membrane state $y(t)$ and the reversal potential h_{eq}. This block captures a simplified conductance-based membrane integration, and is used for conductance-driven populations (e.g., the Larter–Breakspear model).

Difference readout.

$$\dot{y}(t) = x_i(t) - x_j(t), \tag{27}$$

where $x_i(t)$ and $x_j(t)$ are two designated internal state variables of the two input process blocks within the same population. The resulting state $y(t)$ accumulates the difference between the two designated internal variables and is exported as the population output. This construction is used in the MDF model.

Connectivity functions

Connectivity functions transform inputs into the actual drive that enters a population's state equations. Although functions such as sigmoids or rectifiers are sometimes described as "output transformations" in the neural-mass literature—because they map membrane potentials to firing rates—here we reserve the term *output processes* exclusively for mechanisms that introduce additional state variables. Connectivity functions, in contrast, apply transformations directly to incoming signals already appearing in the population's differential equations. Each population may assign different connectivity functions to inputs coming from other populations, sensory

channels, or internode connections. For a given population, all incoming signals are grouped by the connectivity function c assigned to them. The total transformed input is

$$h(t) = \sum_{c \in \mathcal{C}} h_c\bigl(x_c(t); \phi_c\bigr) + I(t), \tag{28}$$

where $\mathcal{C}$ is the set of all connectivity-function types and ϕ_c are the corresponding parameters. For each connectivity-function group c, the aggregated drive is

$$x_c(t) = \sum_{s \in \mathcal{U}_c} v_s\, u_s(t) + \sum_{s \in \mathcal{Z}_c} w_s\, z_s(t), \tag{29}$$

where $\mathcal{U}_c$ denotes the set of internode inputs processed by connectivity function c, and $\mathcal{Z}_c$ denotes the set of within-node population outputs processed by the same function. Here, $u_s(t)$ are internode inputs, $z_s(t)$ are outputs of other populations within the node, and v_s and w_s are the corresponding coupling weights. If present, the global sensory input $I(t)$ is added separately to the appropriate population input. Currently implemented connectivity functions are:

Linear.

$$h(x) = x. \tag{30}$$

A direct linear pass-through of the aggregated input, without parameters.

Saturating logistic sigmoid.

$$h(x) = \frac{e}{1 + \exp\bigl(\rho(\theta - x)\bigr)}. \tag{31}$$

A classical logistic nonlinearity with maximum value e, slope parameter ρ, and midpoint (threshold) θ. Implements the firing-rate transfer used in many neural-mass models.

Baseline-subtracted logistic sigmoid.

$$h(x) = g\left(\frac{1}{1 + \exp\bigl(a(\theta - x)\bigr)} - \frac{1}{1 + \exp(a\theta)}\right). \tag{32}$$

A logistic sigmoid shifted so that $h(0) = 0$. Parameter a controls steepness, θ the midpoint, and g is an overall gain.

Relaxed rectifier (soft threshold).

$$h(x) = \frac{ax - b}{1 - \exp\bigl(-d(ax - b)\bigr)}. \tag{33}$$

A smooth approximation of a rectified linear response. Parameter a is an input gain, b a threshold, and d controls the softness of the transition.

Connectivity motifs

Connectivity motifs specify the adjacency pattern among populations within a node. A full binary connectivity matrix on n populations has n^2 possible directed edges, so the number of distinct adjacency patterns grows as 2^{n^2}. For $n = 5$, this already yields $2^{25} \approx 3.4 \times 10^7$ possibilities, which would make connectivity choices dominate the grammar's model space. To avoid this combinatorial explosion and to bias models toward interpretable structures, we restrict the grammar to a finite library of reusable connectivity motifs.

For $n \leq 2$, the grammar can enumerate all sensible $n \times n$ patterns (i.e. those that actually connect the populations). For $n > 2$, instead of sampling an arbitrary binary matrix, the grammar selects one of several predefined motifs and instantiates it at size n. These motifs are implemented as Boolean adjacency masks and fall into two broad categories: *deterministic* motifs, which come from canonical models and graph theory, and the seeded *random* motifs, which generate adjacency patterns from standard network models, e.g., Erdos–Renyi networks.

Motifs drastically reduce the model space compared to sampling arbitrary adjacency matrices: for an n-population node, a free adjacency matrix has 2^{n^2} possibilities, while a motif chooses one of $\mathcal{O}(10)$ families with fixed or parameterised structure.

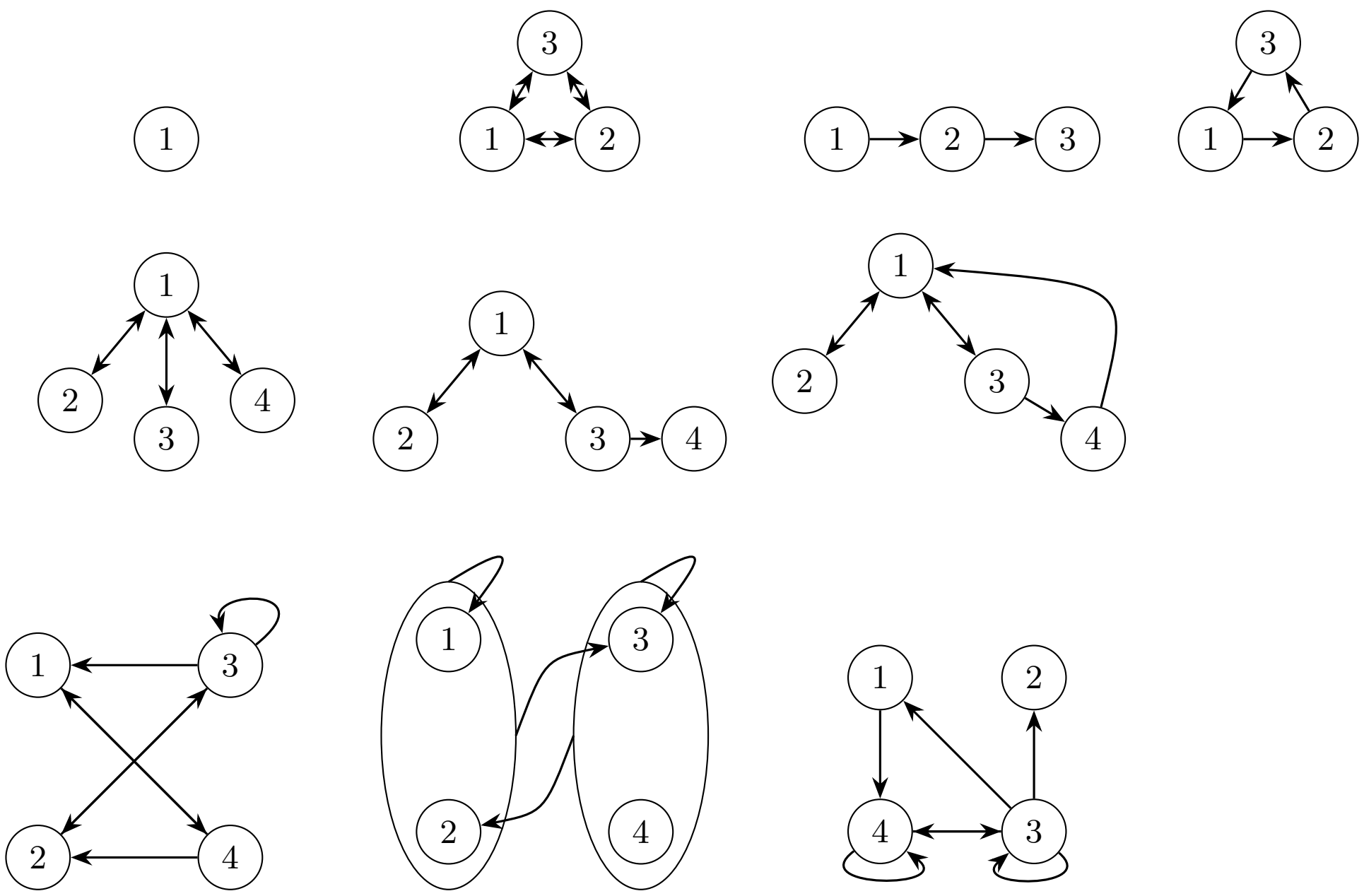

Fig A. Connectivity motifs used for population coupling. In order: Null, Full, Chain, Ring, Star, Star Tail, Star Feedback Tail, Star Loop Extended, EI Extended, and an example of Erdos-Renyi connectivity motif. Arrows indicate directed connections.

Stochastic dynamics

Noise can enter the models in two ways: as external noisy input or as internal stochastic fluctuations. When internal noise is enabled, the builder inserts an SDE diffusion term into the first state variable of each input process block:

$$\mathrm{d}x_i = f_i \, \mathrm{d}t + \sigma_i \, \mathrm{d}W_t. \tag{34}$$

The noise amplitude σ_i is a tunable parameter. Whenever the grammar samples a stochastic specification for a population, the corresponding diffusion term is automatically added to its state equations. External noise, however, is introduced only through the manually constructed sensory input and is not sampled by the grammar.

Additional details about the grammar

This appendix clarifies several aspects of the grammar that are not discussed in detail in the main manuscript.

To reduce the search space and ensure structurally sensible sampled models, we impose two context-dependent constraints during grammar sampling. First, the choice of output process depends on the number of distinct inputs received by a population. In particular, output processes that operate on a single input variable are only allowed for populations with one input process, whereas difference-based outputs are only allowed when two input processes are present. Second, when a population receives multiple inputs, the associated input processes are constrained to share the same dynamical form. This reflects a common feature of canonical neural population models, in which a given population typically integrates different incoming signals through the same synaptic or filtering mechanism.

The sampled grammar parse tree is mostly abstract: most terminal symbols specify classes of dynamical processes rather than explicit differential equations. For example, the production `InputProcess` → `Second-order` selects a second-order synaptic kernel without yet instantiating its concrete state equations. In contrast, the production `InputProcess` → `S S` recursively expands algebraic expressions and directly constructs polynomial right-hand sides during grammar expansion. The resulting formalism is therefore hybrid, combining abstract process-level model specification with direct equation generation. This enables both broad exploration of structurally constrained model families and targeted searches over explicit dynamical mechanisms.

Although these dynamical families are rarely considered within a single unified modelling space, they are not conceptually incompatible. ENEEGMA treats each population type as a modular dynamical block with well-defined inputs and outputs, enabling systematic exploration of model combinations that are uncommon in the literature yet remain mathematically coherent. This does not imply that all such hybrid models are physiologically plausible; rather, it supports structured comparison across modelling assumptions and may help identify intermediate formulations bridging established model families.

Because the recursive polynomial productions impose no a priori bound on expression length or degree, the space of admissible node models is countably infinite. The grammar can therefore generate arbitrarily complex dynamical systems while remaining within a fixed and interpretable structural template.

Jansen–Rit model example

To illustrate how a familiar neural mass model is generated, we use the classical Jansen–Rit (JR) cortical column as an example. Rather than hard-coding the JR equations, ENEEGMA recovers the model through a sequence of grammar expansions that progressively refines the abstract start symbol `Node` into a concrete system of coupled population dynamics.

For the JR model, the symbol `Node` is expanded into a three-population configuration together with one population-coupling function, three sensory-coupling-function slots, and one connectivity motif. This structure is obtained by expanding `Pops2` into two `Pop1` symbols, thereby fixing the total number of populations to three. Each population is then specified by the production

`Pop1` → `InputProcess OutputProcess ECF ECF Stochasticity`,

which determines how presynaptic input is transformed into postsynaptic state variables, how those states are exposed as outputs, and whether stochastic noise is present.

For the JR model, the grammar selects the second-order PSP kernel for `InputProcess` in all three populations, thereby fixing the postsynaptic dynamics to a second-order linear system. It also selects direct readout for `OutputProcess`, so that the membrane-potential-like state of each population is transmitted without additional output dynamics.

In ENEEGMA, this corresponds to instantiating the unified second-order postsynaptic potential (PSP) kernel

$$\dot{x}_1(t) = x_2(t), \tag{35}$$
$$\dot{x}_2(t) = \omega^2(g\,u(t) - x_1(t)) - 2\,\zeta\,\omega\,x_2(t), \tag{36}$$

where $x_1(t)$ denotes the lumped PSP variable and $x_2(t)$ its temporal derivative. The JR model is recovered by choosing the critically damped case $\zeta = 1$ and identifying $\omega = 1/\tau$, with $g = A$ for excitatory synapses and $g = B$ for inhibitory synapses. This yields the classical α-kernel system

$$\dot{x}_1(t) = x_2(t), \tag{37}$$
$$\dot{x}_2(t) = A\,a\,u(t) - 2a\,x_2(t) - a^2 x_1(t), \tag{38}$$

with $a = 1/\tau$. The corresponding impulse response,

$$h(t) \propto t\,e^{-at},$$

is the characteristic α-shaped PSP used in the original JR formulation.

To couple the populations within a node, the grammar selects the saturating sigmoid connectivity function, which implements the firing-rate nonlinearity used in the original JR model. Finally, the connectivity-motif nonterminal is resolved to a star-shaped motif, capturing the canonical three-population coupling pattern of the JR column: pyramidal-to-excitatory, excitatory-to-pyramidal, and inhibitory-to-pyramidal interactions.

Through this sequence of expansions, the grammar refines an abstract node description into a fully specified three-population neural mass model whose equations match the classical Jansen–Rit formulation up to notational differences.

References

1. Wong KF, Wang XJ. A recurrent network mechanism of time integration in perceptual decisions. Journal of Neuroscience. 2006;26(4):1314–1328.

2. Deco G, Ponce-Alvarez A, Mantini D, Romani GL, Hagmann P, Corbetta M. Resting-state functional connectivity emerges from structurally and dynamically shaped slow linear fluctuations. Journal of Neuroscience. 2013;33(27):11239–11252.

3. Deco G, Ponce-Alvarez A, Hagmann P, Romani GL, Mantini D, Corbetta M. How local excitation–inhibition ratio impacts the whole brain dynamics. Journal of Neuroscience. 2014;34(23):7886–7898.
