# Supplementary material for: Neural population models for EEG: From Canonical models to alternative model structures
Source: PLoS Comput Biol. 2026 Aug 6;22(8):e1014222. doi: 10.1371/journal.pcbi.1014222 (PMC13460749; doi:10.1371/journal.pcbi.1014222)
Supplement: Supplementary file 4 [file pcbi.1014222.s004.pdf]

Supporting Information

Neural Population Models for EEG: From Canonical Models to Alternative Model Structures

S4 Appendix: Additional canonical-model results

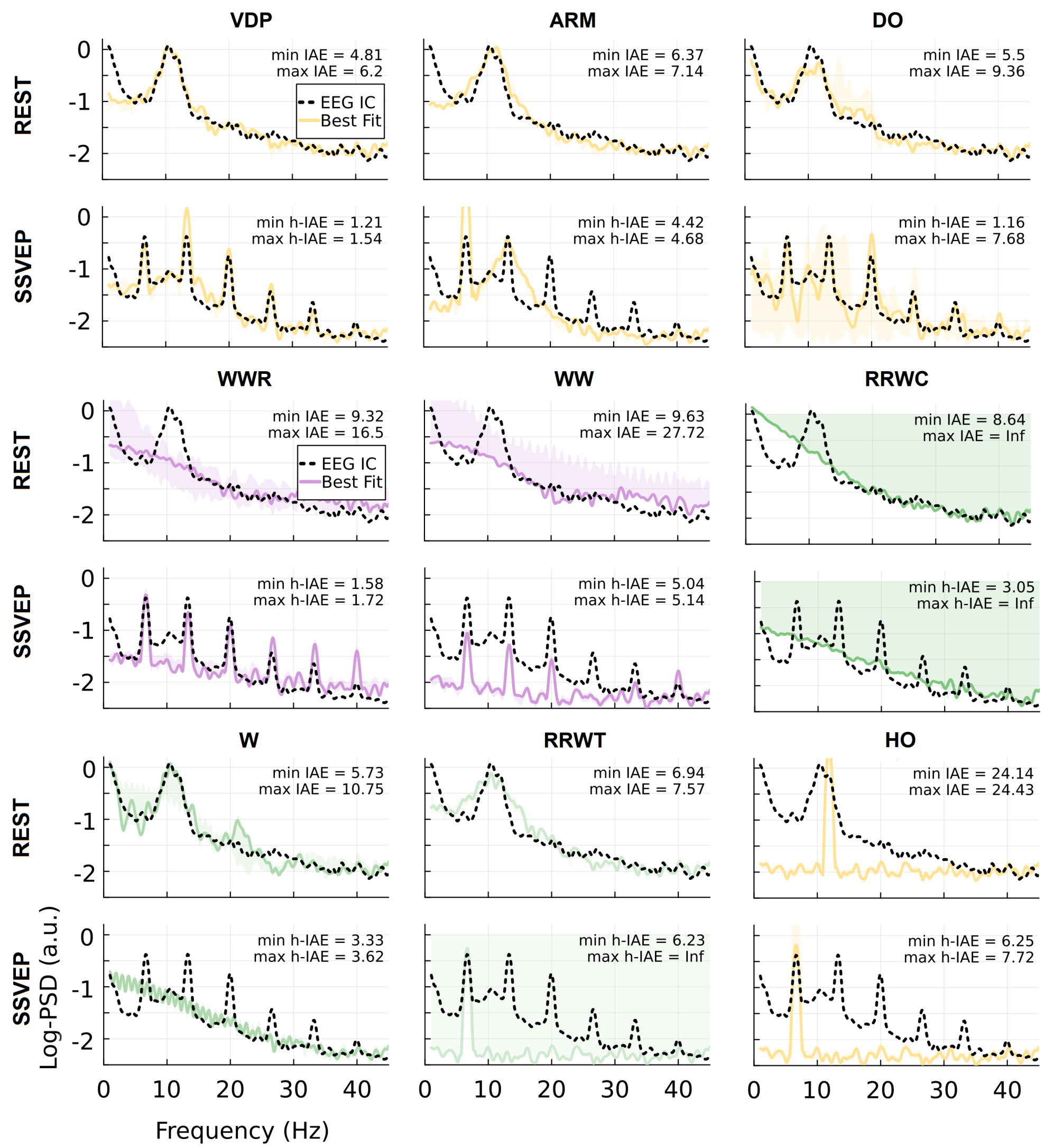

Fig A. Log-PSD fits of additional canonical models. Panels show the canonical models not included in the main-text figure, evaluated for the first representative dataset under RS and SSVEP conditions. Black dashed lines denote empirical log-PSD, colored solid lines the best-fitting simulated spectra, and shaded regions the range (min–max) across five repeated simulations. Five evaluations were performed at fixed fitted parameters, varying initial states and, for RS only, external white-noise realizations. Insets report the minimum and maximum fitting error across repeated simulations (IAE for RS; h-IAE for SSVEP). As in the main text, SSVEP goodness of fit is based on harmonic-restricted error, so close agreement is expected primarily at the stimulus-locked harmonic peaks rather than across the full broadband spectrum.

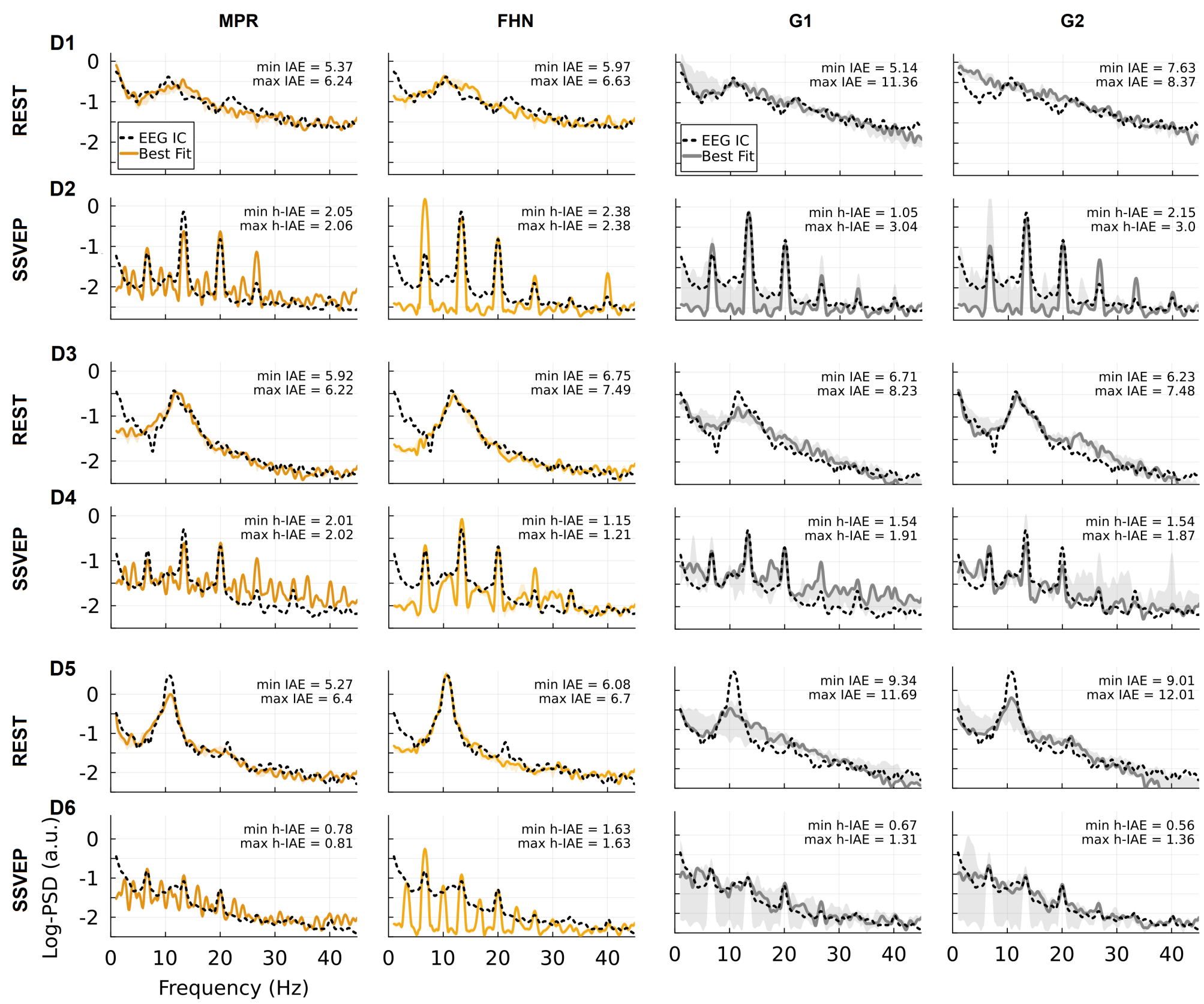

Fig B. Log-PSD fits of top two performing canonical and grammar-generated models across six additional datasets. The left block shows the two top-performing canonical models (MPR and FHN), while the right block shows two top-performing grammar-generated models (G1 and G2). Each dataset is shown in a separate row pair, with RS above SSVEP. Black dashed lines denote empirical log-PSD, solid lines the best-fitting simulated spectra, and shaded regions indicate the range (min–max) across five repeated simulations. Five evaluations were performed at fixed fitted parameters, varying initial states and, for RS only, external white-noise realizations. Insets report the minimum and maximum fitting error across repeated simulations (IAE for RS; h-IAE for SSVEP). As in the main analysis, close SSVEP agreement is expected mainly at the stimulus-locked harmonic peaks because model comparison was based on harmonic-restricted error.
