# Supplementary material for: Neural population models for EEG: From Canonical models to alternative model structures
Source: PLoS Comput Biol. 2026 Aug 6;22(8):e1014222. doi: 10.1371/journal.pcbi.1014222 (PMC13460749; doi:10.1371/journal.pcbi.1014222)
Supplement: Supplementary file 5 [file pcbi.1014222.s005.pdf]

Supporting Information
Neural Population Models for EEG: From Canonical Models to Alternative Model Structures

S5 Appendix: Harmonic generation and simulated time series

This section provides additional analyses addressing harmonic generation and model time-series behavior. Fig A illustrates how harmonic peaks can arise in the single-population MPR model when driven by a sinusoidal input, compared with a noise-only input. Figs B and C show representative non-normalized time-series segments for the eight best-performing and nine remaining canonical models, respectively, during both rest and SSVEP task conditions. Finally, Fig D shows the corresponding time-series segments for the generated models.

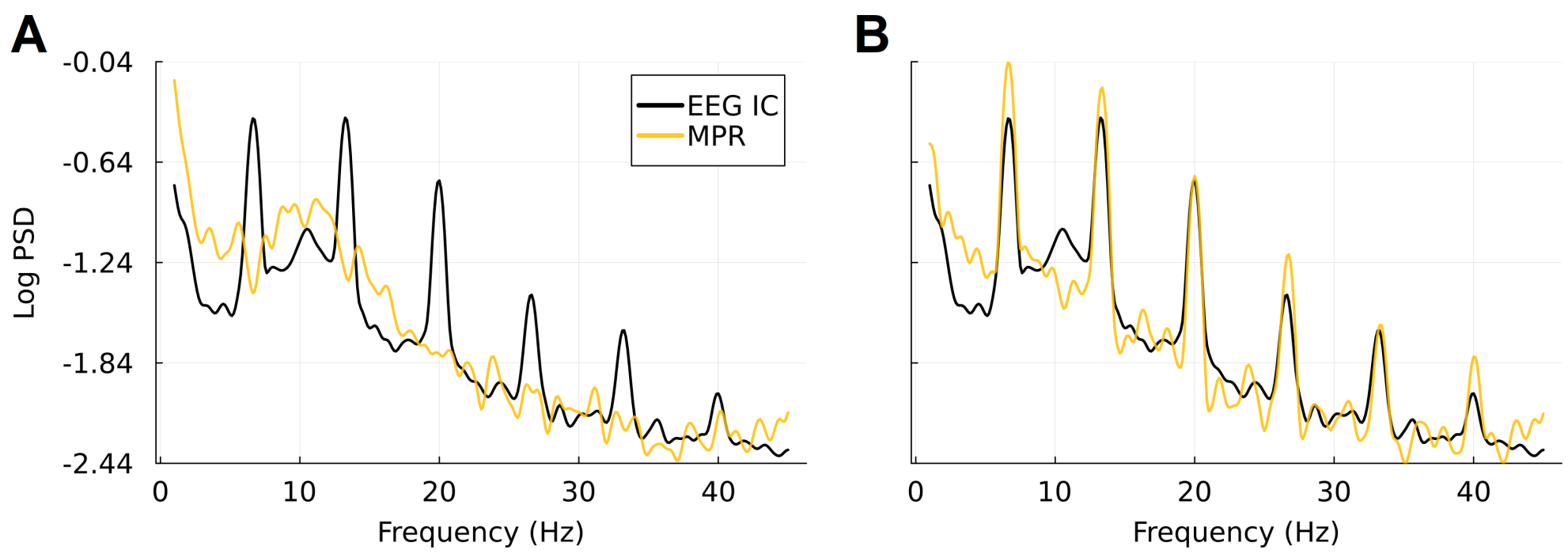

Fig A. Noise-only and sinusoidally driven MPR spectra. Power spectral density fits are shown for the empirical EEG independent component (EEG IC; black) and the MPR model output (orange). **A:** With noise-only input, the MPR model captures the broadband spectral shape but does not generate the harmonic peak structure. **B:** With an additional pure sinusoidal drive at the SSVEP stimulation frequency, harmonics emerge in the model output due to nonlinear dynamics, demonstrating that multiple spectral peaks can arise in a single-population MPR model without requiring multiple interacting populations.

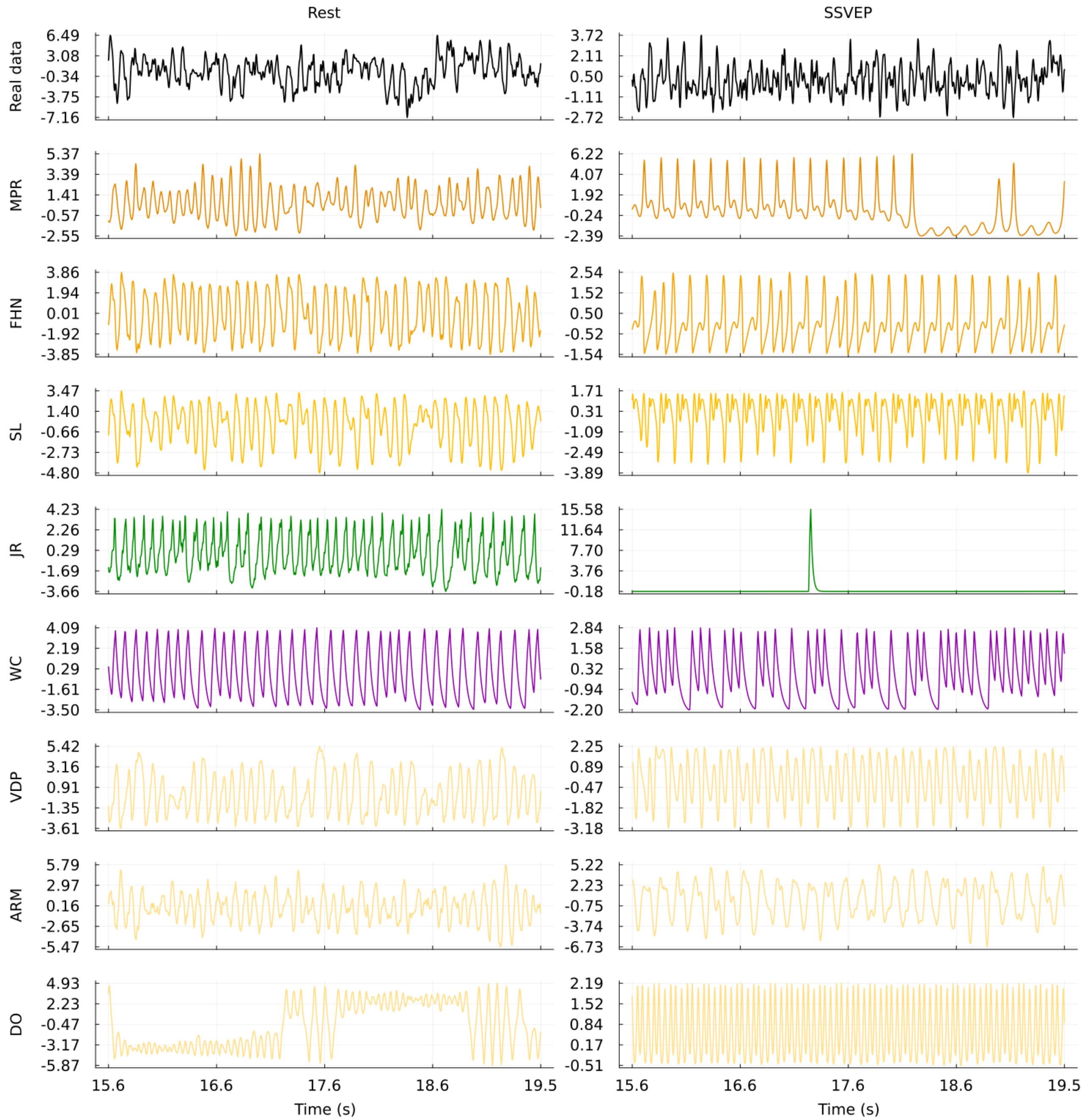

Fig B. Simulated time series for the eight best-performing canonical neural population models during rest and SSVEP tasks. Columns correspond to task condition, as indicated by the column titles, with resting state shown on the left and SSVEP stimulation shown on the right. Each trace shows a 1000-sample segment extracted from the middle of a 60-second simulation, corresponding to approximately 3.9 s at a sampling rate of 256 Hz. The top row shows the empirical data in black, while the remaining rows show the simulated outputs of the canonical models, as indicated by the y-axis labels. For visualization, time series are shown in their original, non-normalized scale; normalization was applied only during the optimization procedure.

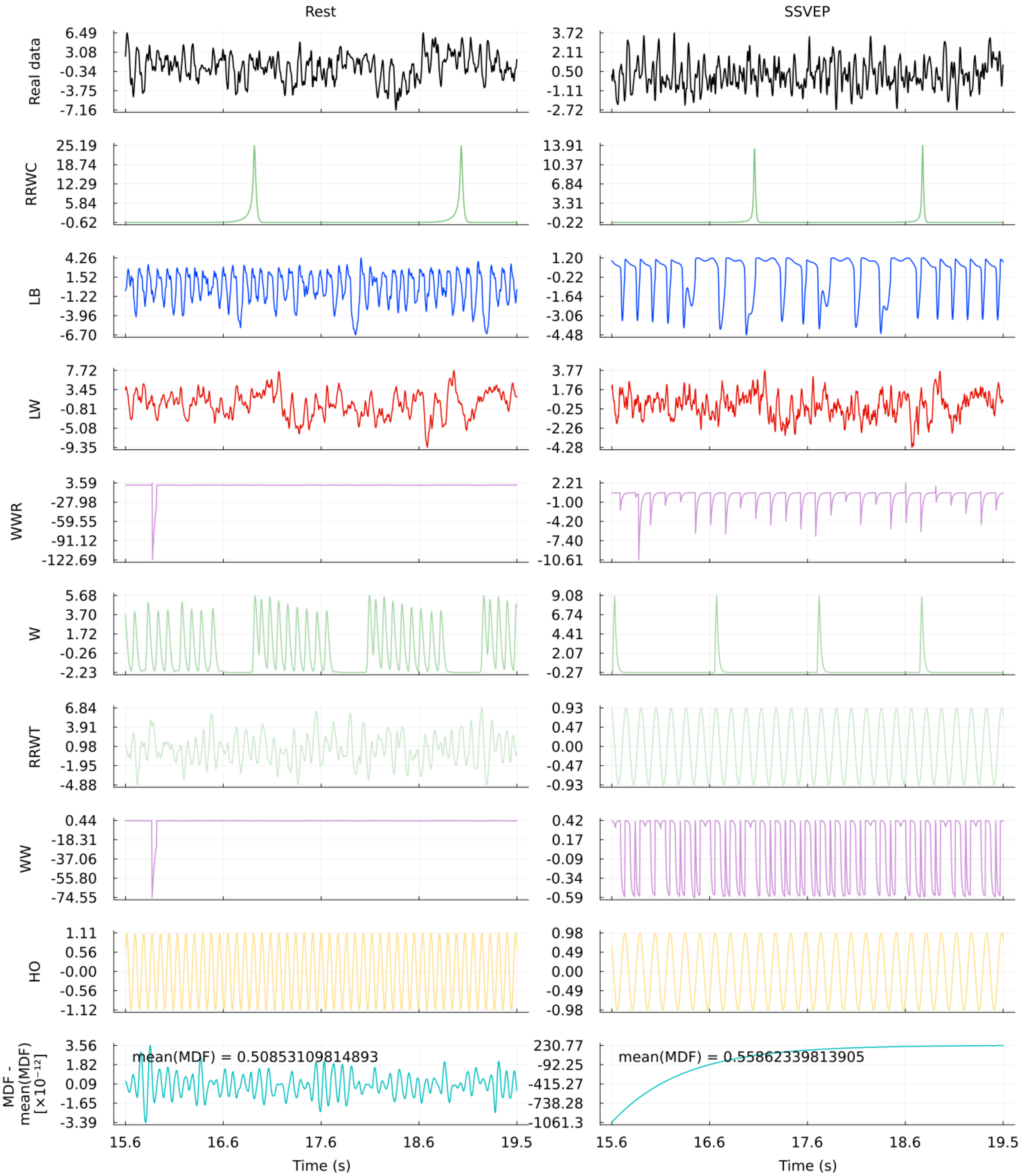

Fig C. Simulated time series for the nine remaining canonical neural population models during rest and SSVEP tasks. Columns correspond to task condition, as indicated by the column titles, with resting state shown on the left and SSVEP stimulation shown on the right. Each trace shows a 1000-sample segment extracted from the middle of a 60-second simulation, corresponding to approximately 3.9 s at a sampling rate of 256 Hz. The top row shows the empirical data in black, while the remaining rows show the simulated outputs of the canonical models, as indicated by the y-axis labels. For visualization, time series are shown in their original, non-normalized scale; normalization was applied only during the optimization procedure.

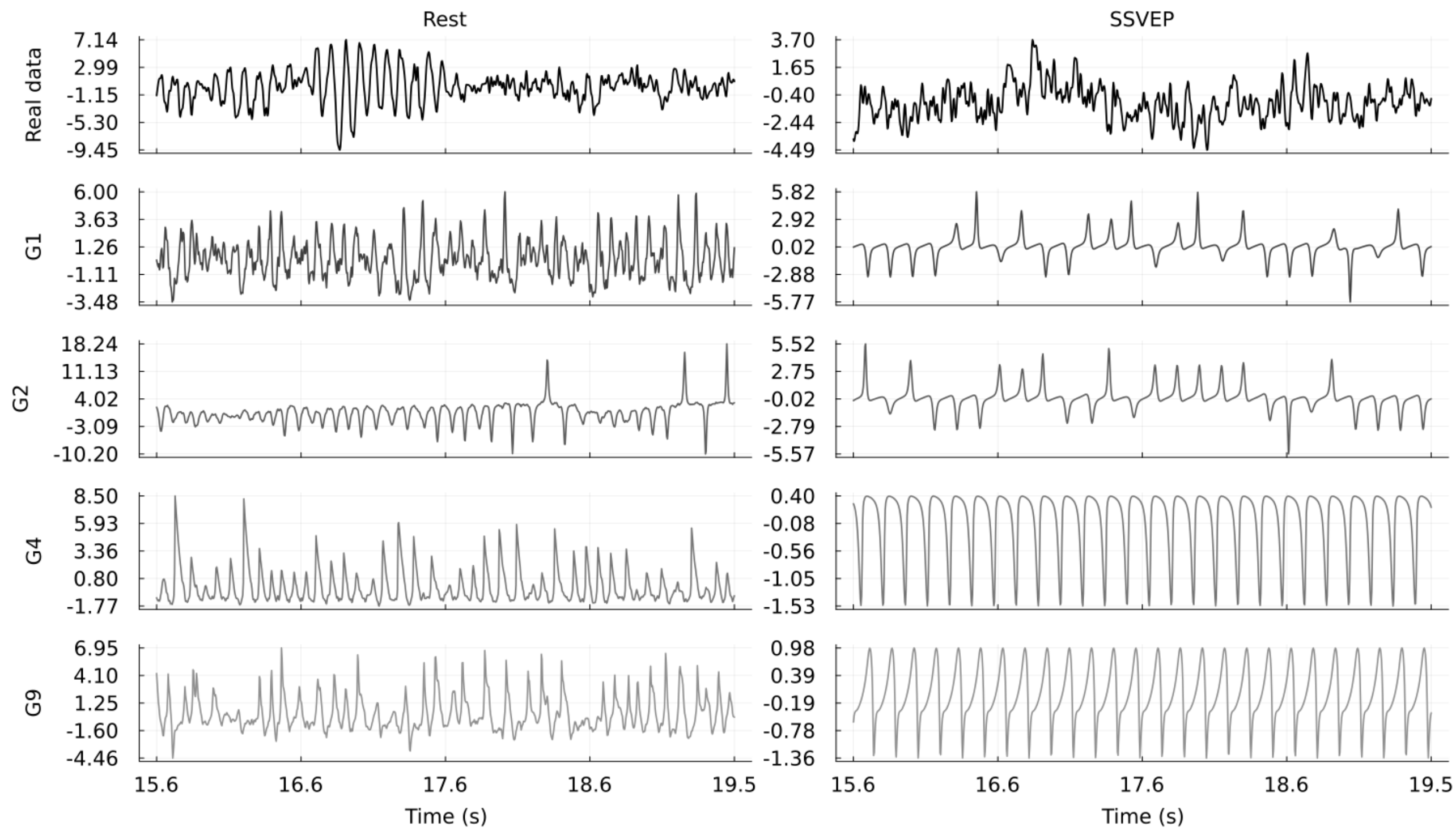

Fig D. Simulated time series for the generated neural population models during rest and SSVEP tasks. Columns correspond to task condition, as indicated by the column titles, with resting state shown on the left and SSVEP stimulation shown on the right. Each trace shows a 1000-sample segment extracted from the middle of a 60-second simulation, corresponding to approximately 3.9 s at a sampling rate of 256 Hz. The top row shows the empirical data in black, while the remaining rows show the simulated outputs of the canonical models, as indicated by the y-axis labels. For visualization, time series are shown in their original, non-normalized scale; normalization was applied only during the optimization procedure.
