# Supplementary material for: Neural population models for EEG: From Canonical models to alternative model structures
Source: PLoS Comput Biol. 2026 Aug 6;22(8):e1014222. doi: 10.1371/journal.pcbi.1014222 (PMC13460749; doi:10.1371/journal.pcbi.1014222)
Supplement: Supplementary file 6 [file pcbi.1014222.s006.pdf]

Supporting Information

Neural Population Models for EEG: From Canonical Models to Alternative Model Structures

S6 Appendix: Generated models and fitted parameters

This appendix lists the generated models discussed in the main text. For each model, we first give the reduced single-node equations in generic parametric form and then report the condition-specific fitted parameter values for the first (representative) dataset, rounded to two decimals. Because the present study considered only single-node dynamics, some distinctions present at the grammar level can disappear during reduction to the realized differential-equation system. In particular, Models G1 and G2 are symbolically identical in their reduced form, even though they arise from different grammar expansions and occupy different fitted parameter regimes across conditions.

Model G1.

$$\dot{x}_{11} = (c_{11} + c_{13}x_{11}x_{12} + c_{15}\,\mathrm{I}(t))\,\tau_1, \tag{1}$$

$$\dot{x}_{12} = \left(c_{12} + c_{14}x_{11}^2 + c_{16}\,\mathrm{I}(t)\right)\tau_1. \tag{2}$$

For RS, the fitted parameters for Dataset 1 were $c_{11} = 0.33$, $c_{12} = 5.73$, $c_{13} = 3.14$, $c_{14} = -0.79$, $c_{15} = -1.65$, $c_{16} = 5.75$, and $\tau_1 = 13.54$.

For SSVEP, the fitted parameters for Dataset 1 were $c_{11} = -1.30$, $c_{12} = -1.69$, $c_{13} = -1.33$, $c_{14} = 0.98$, $c_{15} = -1.74$, $c_{16} = 5.23$, and $\tau_1 = 26.93$.

Model G2.

$$\dot{x}_{11} = (c_{11} + c_{13}x_{11}x_{12} + c_{15}\,\mathrm{I}(t))\,\tau_1, \tag{3}$$

$$\dot{x}_{12} = \left(c_{12} + c_{14}x_{11}^2 + c_{16}\,\mathrm{I}(t)\right)\tau_1. \tag{4}$$

For RS, the fitted parameters for Dataset 1 were $c_{11} = -1.52$, $c_{12} = 6.00$, $c_{13} = 2.41$, $c_{14} = -0.35$, $c_{15} = 5.96$, $c_{16} = 3.47$, and $\tau_1 = 14.27$.

For SSVEP, the fitted parameters for Dataset 1 were $c_{11} = -0.45$, $c_{12} = 5.81$, $c_{13} = 5.54$, $c_{14} = -1.71$, $c_{15} = 6.00$, $c_{16} = 3.25$, and $\tau_1 = 7.44$.

Model G4.

$$\dot{x}_{11} = \left(c_{11} + c_{13}x_{12} + c_{14}x_{11} + c_{15}x_{11}^3x_{12} + c_{17}\,\mathrm{I}(t)\right)\tau_1, \tag{5}$$

$$\dot{x}_{12} = \left(c_{12} + c_{16}x_{11}^2x_{12} + c_{18}\,\mathrm{I}(t)\right)\tau_1. \tag{6}$$

For RS, the fitted parameters for Dataset 1 were $c_{11} = -1.00$, $c_{12} = -1.65$, $c_{13} = -1.51$, $c_{14} = -1.76$, $c_{15} = -1.24$, $c_{16} = -1.68$, $c_{17} = 3.30$, $c_{18} = -0.16$, and $\tau_1 = 37.92$.

For SSVEP, the fitted parameters for Dataset 1 were $c_{11} = 3.58$, $c_{12} = 3.56$, $c_{13} = -1.72$, $c_{14} = -1.01$, $c_{15} = -1.42$, $c_{16} = -1.71$, $c_{17} = 5.84$, $c_{18} = 5.95$, and $\tau_1 = 37.85$.

Model G9.

$$\dot{x}_{11} = \left(c_{11} + c_{15}x_{12} + c_{13}x_{11}^2 + c_{14}x_{11}^2x_{12} + c_{16}x_{11}^3 + c_{19}\,\mathrm{I}(t)\right)\tau_1, \tag{7}$$

$$\dot{x}_{12} = \left(c_{12} + c_{17}x_{12} + c_{18}x_{11} + c_{110}\,\mathrm{I}(t)\right)\tau_1. \tag{8}$$

For RS, the fitted parameters for Dataset 1 were $c_{11} = -16.49$, $c_{12} = 22.65$, $c_{13} = 48.26$, $c_{14} = 19.81$, $c_{15} = 41.38$, $c_{16} = -17.68$, $c_{17} = 20.01$, $c_{18} = -19.43$, $c_{19} = 46.78$, $c_{110} = 26.50$, and $\tau_1 = 2.74$.

For SSVEP, the fitted parameters for Dataset 1 were $c_{11} = -1.44$, $c_{12} = -1.39$, $c_{13} = 5.82$, $c_{14} = -1.30$, $c_{15} = -1.24$, $c_{16} = -1.63$, $c_{17} = 0.90$, $c_{18} = 5.89$, $c_{19} = -0.39$, $c_{110} = 4.59$, and $\tau_1 = 24.87$.
