# Supplementary material for: Neural population models for EEG: From Canonical models to alternative model structures
Source: PLoS Comput Biol. 2026 Aug 6;22(8):e1014222. doi: 10.1371/journal.pcbi.1014222 (PMC13460749; doi:10.1371/journal.pcbi.1014222)
Supplement: Supplementary file 7 [file pcbi.1014222.s007.pdf]

Supporting Information
Neural Population Models for EEG: From Canonical Models to Alternative Model Structures

S7 Appendix: Performance of generated models across population–connectivity configurations

To address whether generated-model performance was associated with coarse circuit architecture, we performed an additional targeted analysis by grouping generated models according to their population–connectivity configuration. This configuration was defined by the number of instantiated populations and the realized within-node connectivity motif after model construction. The analysis should be interpreted as a supplementary structural check rather than a complete empirical comparison of all grammar regions: some configurations were sampled specifically to broaden the range of population counts and connectivity motifs, and not all corresponding grammar regions were analyzed as extensively as the main generated-model subset discussed in the Results.

For each generated model, we summarized performance using a robust best-hyperparameter IAE. For each hyperparameter configuration, the median IAE was computed across repeated runs, with failed, missing, or non-finite runs assigned a large penalty value. The model-level score was then defined as the lowest of these hyperparameter-level medians. This score captures the best robust performance achieved by the model while penalizing unstable hyperparameter configurations. The results are shown in Fig A.

Because the population–connectivity classes were highly unequal in size, statistical comparisons were interpreted together with effect sizes and repeated after excluding classes with fewer than 30 models. Across all finite model-level scores, the global Kruskal–Wallis test detected a small departure from identical distributions across configurations (H=31.40, p=0.0017), but the effect size was negligible ($\epsilon^2 = 0.0052$). We additionally repeated the test within the low-error regime visualized in Fig A, corresponding to the subset of models with (IAE $\leq$ 20). Within this plotted subset, there was no evidence of systematic differences across configurations (H=4.10, p=0.663), and the effect size was approximately zero. Thus, this supplementary analysis did not support a strong dependence of low-error generated-model performance on population–connectivity configuration alone.

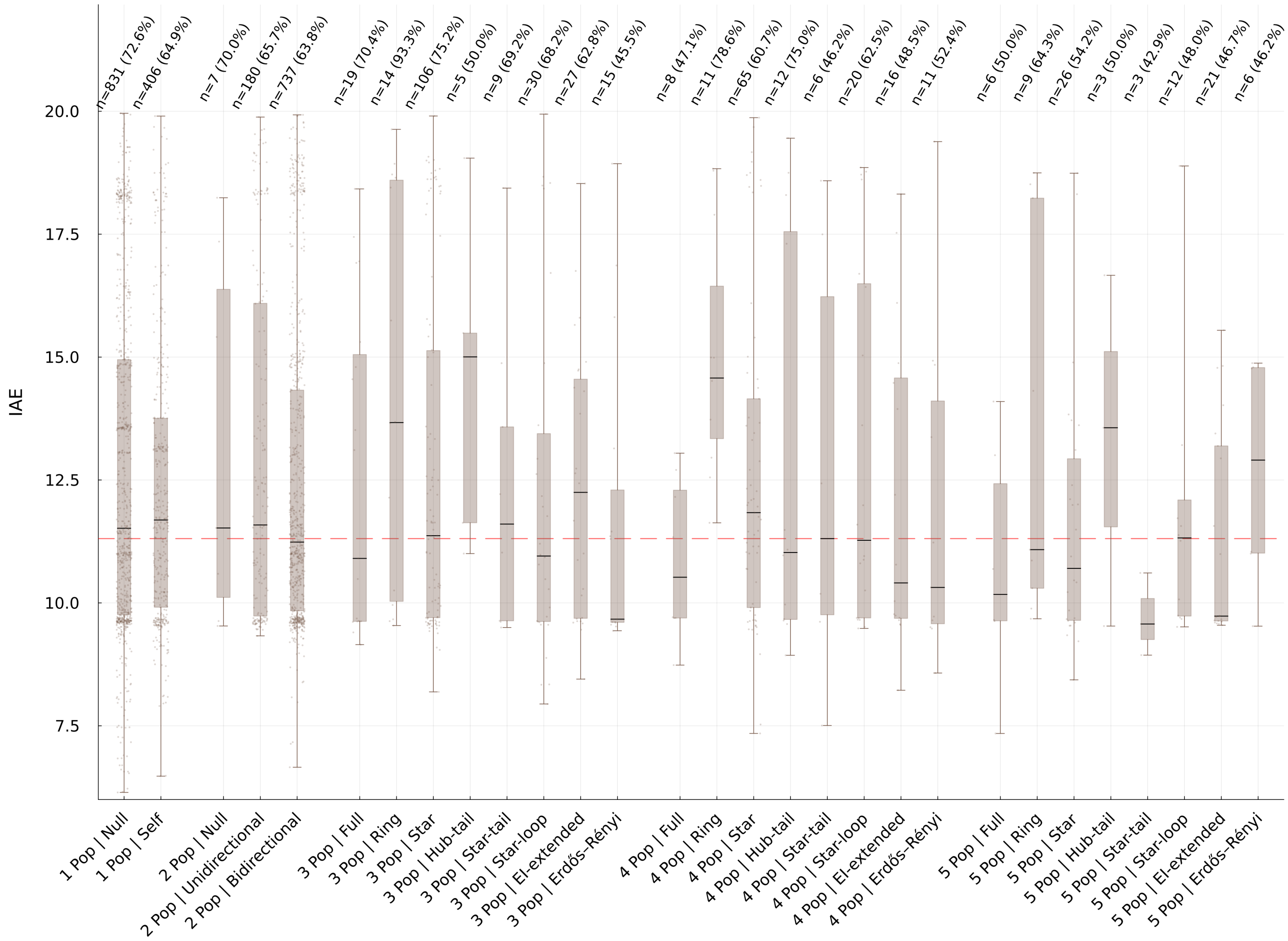

Fig A. Generated-model performance across population–connectivity configurations. Boxplots show best-hyperparameter IAE values for a targeted supplementary set of grammar-generated models grouped by population–connectivity configuration, defined by the number of instantiated populations and the realized within-node connectivity motif. For each generated model, the median IAE was first computed across repeated runs within each hyperparameter configuration, with failed, missing, or non-finite runs assigned a large penalty value; the plotted model-level value is the lowest of these hyperparameter-level medians. Only models with selected median IAE (≤ 20) are shown to visualize the low-error regime. Labels above each box indicate the number of plotted models and the corresponding percentage of finite model-level scores in that configuration that fell below this low-error regime threshold. The dashed horizontal line indicates the global median across plotted models. The broad overlap between configurations suggests that low-error fits were not confined to a single population count or realized connectivity motif.
