# Supplementary material for: Neural population models for EEG: From Canonical models to alternative model structures
Source: PLoS Comput Biol. 2026 Aug 6;22(8):e1014222. doi: 10.1371/journal.pcbi.1014222 (PMC13460749; doi:10.1371/journal.pcbi.1014222)
Supplement: Supplementary file 8 [file pcbi.1014222.s008.pdf]

Supporting Information

Neural Population Models for EEG: From Canonical Models to Alternative Model Structures

S8 Appendix: Model complexity, parameter scale, and fitting robustness

To further examine whether differences in model performance could be partly explained by optimization and numerical robustness, we related both fitting error and fitting success rate to simple model-level descriptors. Final error showed only weak-to-moderate associations with the number of fitted parameters and the number of state equations, indicating that complexity alone did not determine the quality of successful fits. In contrast, fitting success rate showed clearer associations with model complexity and especially with the aggregate magnitude of literature/default parameter values, which showed the strongest descriptive association among the quantities examined. Although this aggregate parameter scale is not physically meaningful in an absolute sense because parameters have different units, it provides an exploratory proxy for parameter-scale heterogeneity. Models with larger default parameter magnitudes may contain stronger gains, faster rates, steeper nonlinearities, or conductance-like terms, making their dynamics more sensitive to parameter perturbations and more likely to enter unstable or stiff regimes during optimization. These results support the interpretation that poor robustness of some canonical models reflects not only biological or spectral inadequacy, but also practical difficulties associated with parameter scaling, optimization bounds, and numerical stability.

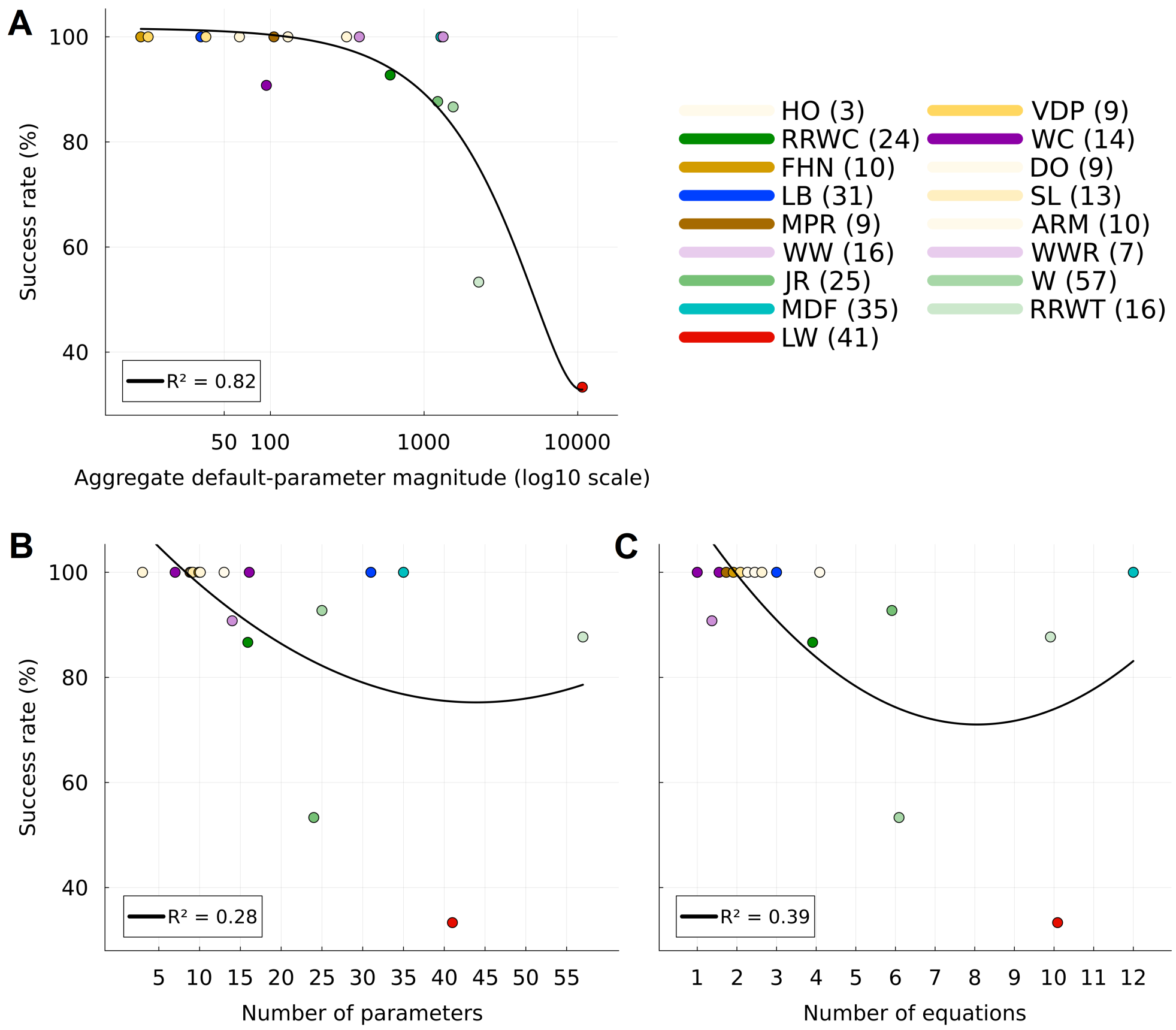

Fig A. Relationship between fitting success rate, model complexity, and parameter scale. Fitting success rate is shown as a function of three model-level quantities: (A) aggregate default-parameter magnitude, (B) number of fitted parameters, and (C) number of state equations. Quadratic fits are included only as descriptive summaries. The clearest negative association was observed for aggregate default-parameter magnitude, whereas the associations with parameter count and state-equation count were weaker. Because parameters have different units and biological meanings, aggregate default-parameter magnitude should not be interpreted as a physically meaningful complexity measure. Instead, it provides an exploratory proxy for parameter-scale heterogeneity, suggesting that fitting robustness depends not only on the number of free parameters or state variables, but also on the numerical scale and sensitivity of the model parameterization.

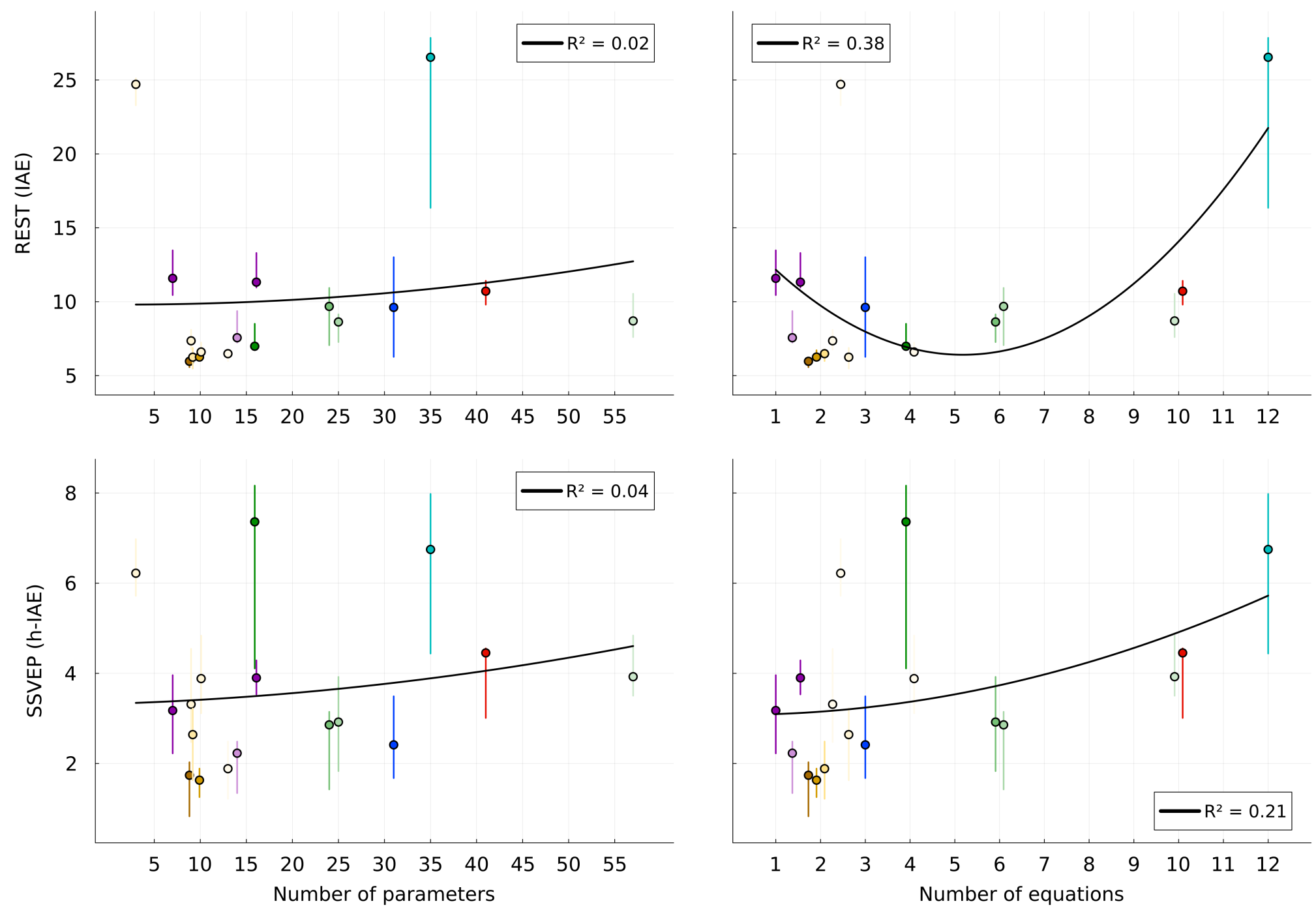

Fig B. Relationship between fitting error and model complexity. Error values are shown as a function of the number of fitted parameters and the number of state equations for the resting-state task (REST; IAE) and the SSVEP task (h-IAE). Curves show quadratic fits used only as descriptive summaries. The weak-to-moderate relationships indicate that final spectral error among successful fits is not determined by complexity alone. Rather, models with different structural complexity can achieve comparable errors once stable parameter regimes are found.
