# Supplementary material for: Neural population models for EEG: From Canonical models to alternative model structures
Source: PLoS Comput Biol. 2026 Aug 6;22(8):e1014222. doi: 10.1371/journal.pcbi.1014222 (PMC13460749; doi:10.1371/journal.pcbi.1014222)
Supplement: Supplementary file 9 [file pcbi.1014222.s009.pdf]

Supporting Information

Neural Population Models for EEG: From Canonical Models to Alternative Model Structures

S9 Appendix: Parameterization and dynamical regime of the generalized MPR and G1 models

Here we report additional analysis of the dynamics of the best canonical model, MPR, and best generated model, G1.

To analyze MPR, we first tried to relate the fitted MPR coefficients to the dynamical regimes reported by Montbrió et al. [1]. Fixed points were calculated with the external input set to zero. For the SSVEP fit, this corresponds to removing the sinusoidal drive before the analysis.

The original nondimensional MPR equations are

$$\dot{x}_1 = \frac{\Delta}{\pi} + 2x_1x_2, \tag{1}$$

$$\dot{x}_2 = x_2^2 + \bar{\eta} + Jx_1 - \pi^2 x_1^2 + u(t), \tag{2}$$

where x_1 denotes population firing rate, x_2 denotes mean membrane potential, Δ controls neuronal heterogeneity, $\bar{\eta}$ is the mean excitability, and J is the recurrent coupling strength. In ENEEGMA, the fitted generalized form is

$$\dot{x}_1 = s\left[c_{11} + c_{13}x_1x_2 + c_{17}u(t)\right], \tag{3}$$

$$\dot{x}_2 = s\left[c_{12} + c_{14}x_1 + c_{15}x_1^2 + c_{16}x_2^2 + c_{18}u(t)\right], \tag{4}$$

where the coefficients were optimized independently and s is a common multiplier that controls the overall time scale.

After factoring out s, the native nondimensional MPR equations correspond to

$$c_{11} = \frac{\Delta}{\pi}, \qquad c_{12} = \bar{\eta}, \qquad c_{13} = 2, \tag{5}$$

$$c_{14} = J, \qquad c_{15} = -\pi^2, \qquad c_{16} = 1. \tag{6}$$

The native formulation additionally requires $c_{17} = 0$, because the external input enters only the membrane-potential equation. For unit input gain, $c_{18} = 1$; in the generalized implementation, c_{18} was optimized independently and can therefore be interpreted as a fitted input-gain coefficient. Because the fixed-point analysis was performed with $u(t) = 0$, neither c_{17} nor c_{18} affects the fixed-point locations or their stability.

The representative fitted coefficients did not satisfy the native MPR relationships, which are: ... For RS, the fitted coefficients were $c_{13} = 34.418$, $c_{15} = -0.474$, and $c_{16} = 11.808$, rather than the native values 2, $-\pi^2$, and 1, respectively. For SSVEP, the corresponding fitted values were $c_{13} = 61.031$, $c_{15} = -0.825$, and $c_{16} = 7.507$. Moreover, c_{17} was nonzero in both conditions. The generalized fits therefore cannot be reduced to a native MPR parameterization or assigned exact coordinates in the parameter plane of Fig 1a in Montbrió et al.

Nevertheless, the fixed-point organization of the generalized fits can be compared qualitatively with the regimes of the native MPR model. With the external input set to zero, both representative fits exhibited four real fixed points. In the physically relevant

region $x_1 \geq 0$, each system contained a low-activity stable node, a saddle, and a high-activity stable focus. The coexistence of two stable fixed points separated by a saddle indicates a bistable fixed-point topology.

Thus, both representative generalized MPR fits exhibited a fixed-point organization qualitatively analogous to the bistable regime shown in Fig 1a of Montbrió et al. [1].

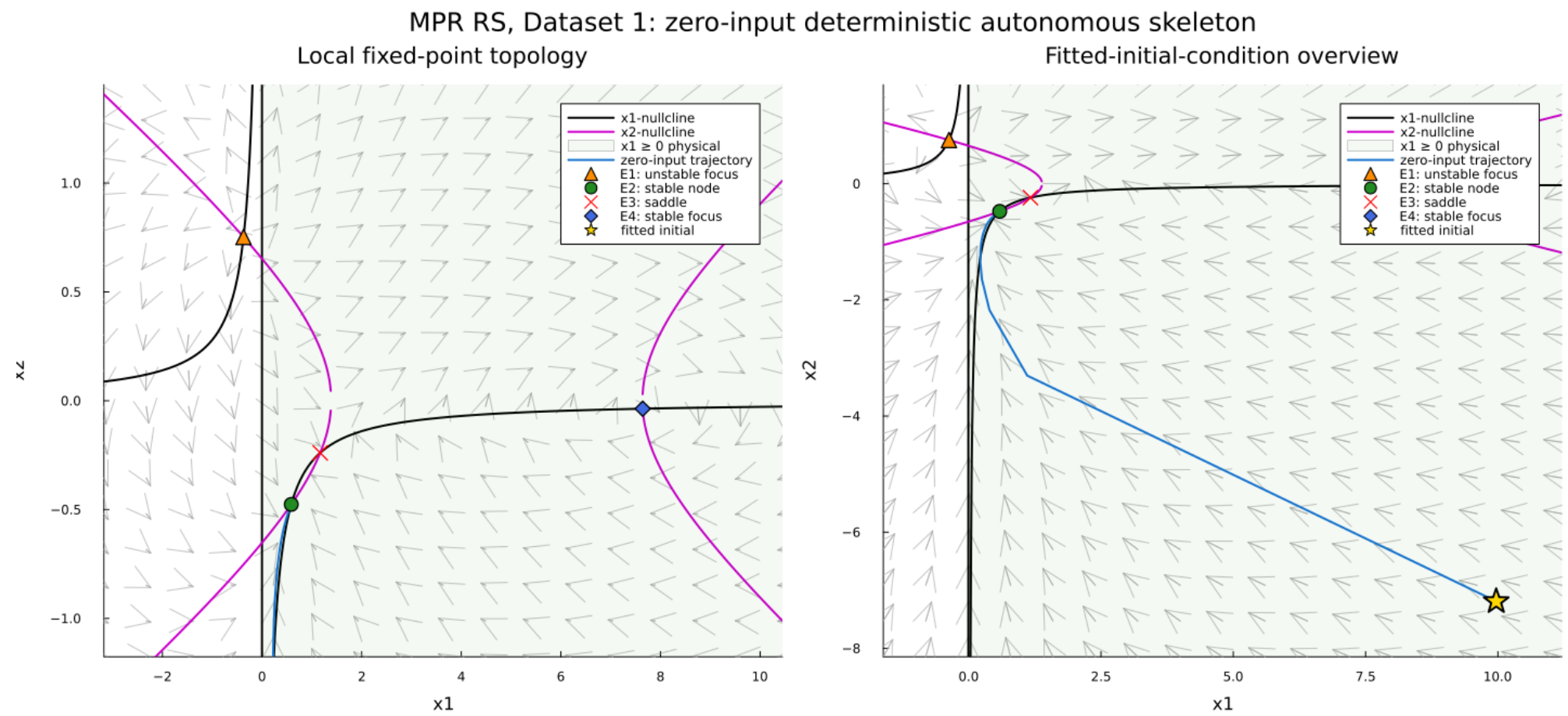

Fig A. Phase plane of the representative generalized MPR fit for RS. The analysis was performed with the external input set to zero. The physically relevant positive firing-rate region contains a low-activity stable node at $(x_1, x_2) = (0.587, -0.476)$, a saddle at $(1.167, -0.239)$, and a high-activity stable focus at $(7.643, -0.037)$. The fitted initial condition converged to the low-activity stable node. The coexistence of two stable fixed points separated by the saddle indicates a bistable topology.

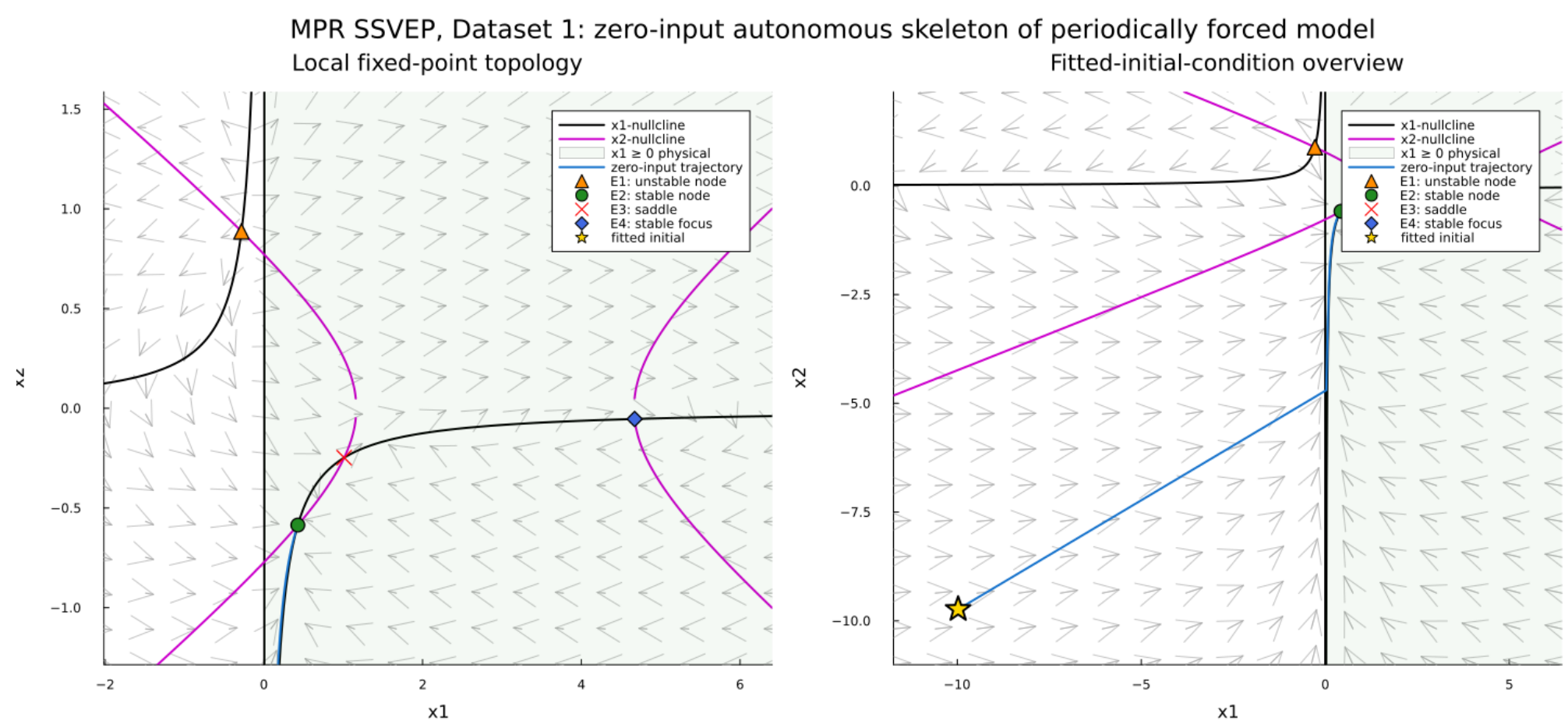

Fig B. Phase plane of the representative generalized MPR fit for SSVEP. The sinusoidal input was removed before the fixed-point analysis. The physically relevant positive firing-rate region contains a low-activity stable node at $(x_1, x_2) = (0.428, -0.586)$, a saddle at $(1.012, -0.248)$, and a high-activity stable focus at $(4.672, -0.054)$. The fitted initial condition converged to the low-activity stable node. As in RS, the coexistence of two stable fixed points separated by a saddle indicates a bistable topology.

We applied the same zero-input fixed-point analysis to representative fits of G1. For the SSVEP fit, the sinusoidal input was removed before the analysis. In both conditions,

G1 exhibited two real fixed points: one stable focus and one unstable focus. Unlike the generalized MPR fits, G1 did not exhibit two stable equilibria separated by a saddle and therefore did not show a bistable fixed-point topology.

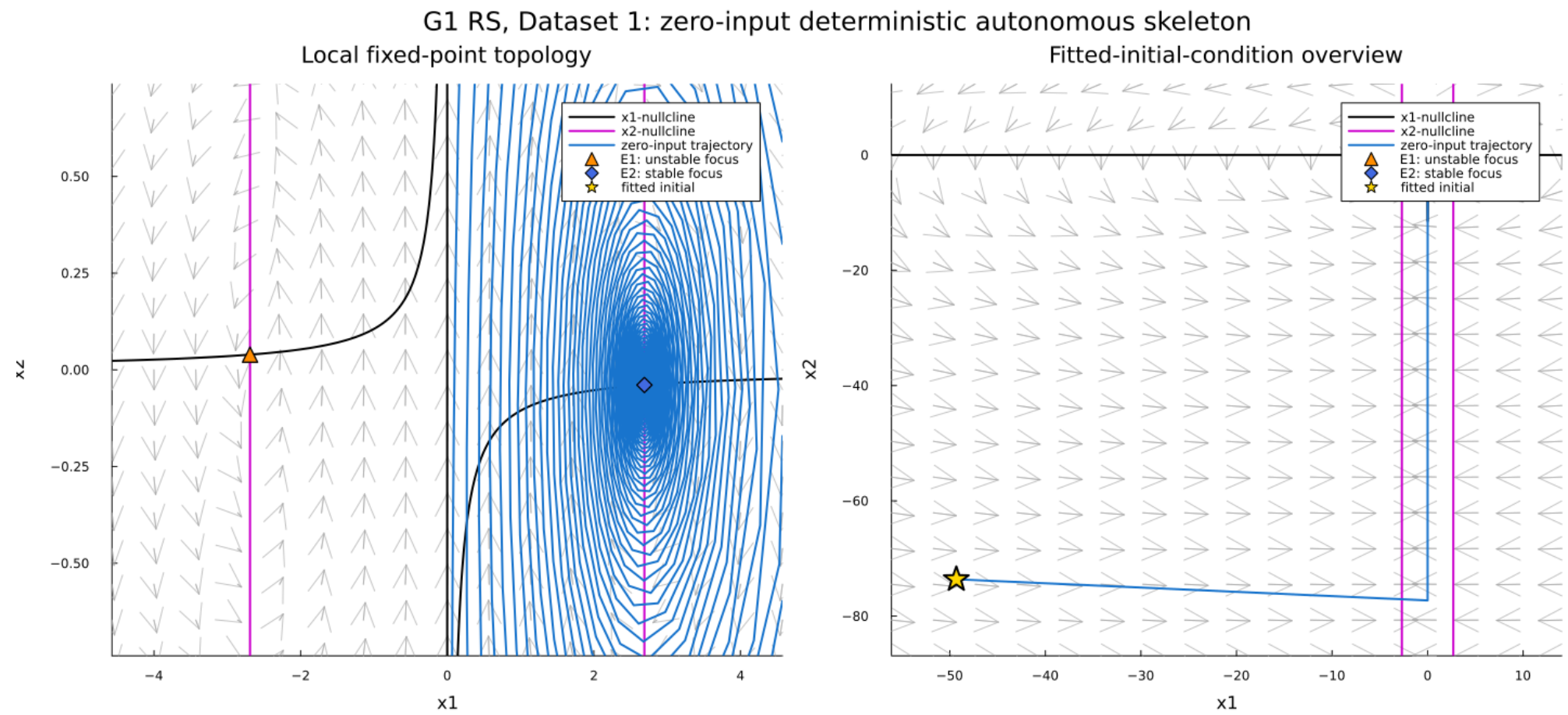

Fig C. Phase plane of the representative G1 fit for RS. The analysis was performed with the external input set to zero. The system contains an unstable focus at $(x_1, x_2) = (-2.690, 0.039)$ and a stable focus at $(x_1, x_2) = (2.690, -0.039)$. The fitted initial condition converged to the stable focus.

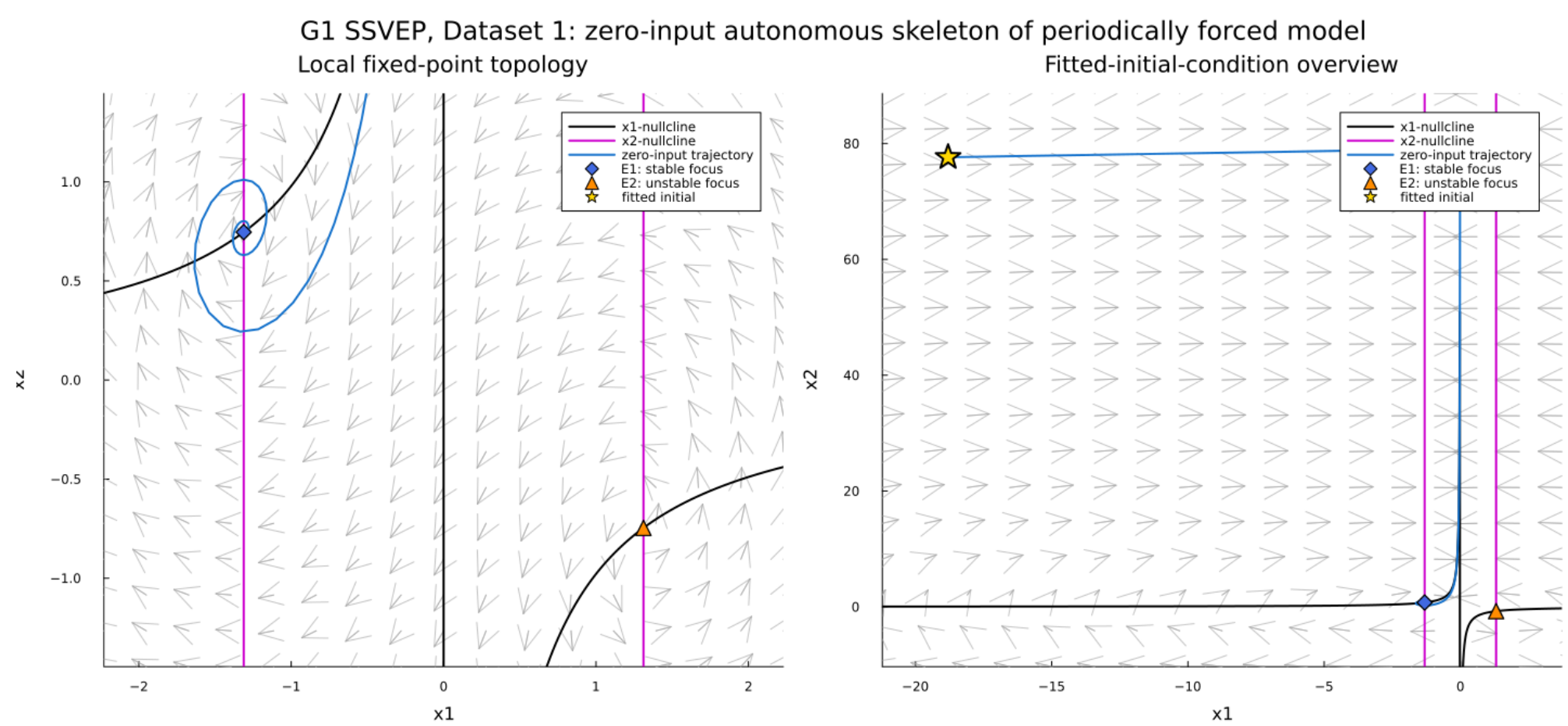

Fig D. Phase plane of the representative G1 fit for SSVEP. The sinusoidal input was removed before the fixed-point analysis. The system contains a stable focus at $(x_1, x_2) = (-1.312, 0.746)$ and an unstable focus at $(x_1, x_2) = (1.312, -0.746)$. Numerical integration from the fitted initial condition converged to the stable focus.

References

1. Montbrió E, Pazó D, Roxin A. Macroscopic description for networks of spiking neurons. Physical Review X. 2015;5(2):021028.
