# Supplementary material for: Neural population models for EEG: From Canonical models to alternative model structures
Source: PLoS Comput Biol. 2026 Aug 6;22(8):e1014222. doi: 10.1371/journal.pcbi.1014222 (PMC13460749; doi:10.1371/journal.pcbi.1014222)
Supplement: Supplementary file 10 [file pcbi.1014222.s011.pdf]

Dear Reviewers,

We would like to thank you for your time and effort to review our manuscript. It was interesting to read your comments and suggestions, for which we are grateful to receive. Please find our responses to your comments below. We first provide your text, point by point, and after each point provide our response, coloured in blue.

Reviewer #1:

This manuscript presents a systematic comparison of 17 canonical models currently used in neuroscience to analyze EEG recordings. The authors employ a Julia-based computational framework they developed, ENEEGMA (Exploring Neural EEG Model Architectures), to compare these models in terms of both mathematical structure and empirical performance. In addition, ENEEGMA is used to generate alternative model architectures with potentially improved performance. The main contribution of the work is thus twofold: the development of the ENEEGMA framework and the results of the comparative analysis. The evaluation is conducted on four EEG datasets under two conditions (resting state and visual-evoked responses).

We appreciate the reviewer's clear and accurate high level summary of the manuscript.

I appreciate the authors' effort to systematically compare neural mass models based on both their mathematical properties and their empirical performance. Although parts of the manuscript describing the design of ENEEGMA draw heavily on machine learning concepts—which are outside my area of expertise and occasionally difficult to follow, I believe the results will be of interest to researchers in computational and mathematical neuroscience. In particular, the study is relevant for those developing and analyzing neural mass models, as well as for researchers focused on EEG data modeling.

The generation of new model architectures is particularly interesting, as it may provide insight into the key mathematical ingredients required to reproduce EEG data. That said, these generated models may or may not be directly related to biophysical mechanisms and may instead fall into the category of phenomenological models. On the other hand, the comparative evaluation of established models is likely to be useful for researchers selecting appropriate modeling frameworks for EEG analysis.

We thank the reviewer for their encouraging assessment and for recognizing the relevance of the work to neural mass modelling and EEG analysis.

Overall, I am positively inclined to recommend this manuscript for publication in PLOS Computational Biology, subject to minor revisions. However, I have several questions and suggestions that should be addressed before publication.

We would also like to thank reviewer for the positive recommendation. We hope we have addressed all your questions and suggestions adequately.

General comment:

I have a general comment regarding the choice and comparison of the canonical models. To clarify

my point, consider the example of the Wilson–Cowan (WC) model. This model describes a canonical neuronal circuit consisting of interacting excitatory (E) and inhibitory (I) populations, each represented by a single ODE describing its activity. This modular structure is shared by many (if not all) neural mass models considered in the manuscript, including the MPR model.

It would be valuable to clarify whether the results and conclusions of the study remain consistent when comparing models built from the same underlying circuit motif (e.g., a single recurrent population or an E–I pair), but implemented using different neural mass formulations. I encourage the authors to discuss this point and, if feasible, to include an additional analysis comparing models at this level.

Such a comparison could be particularly relevant for mechanistic interpretations of EEG activity, linking observed dynamics to underlying circuit motifs (e.g., E–I-driven synchronization, excitatory persistent activity, or inhibitory-driven synchrony). Naturally, this type of comparison would be restricted to neural mass models.

We agree with the reviewer that the NMMs considered in the manuscript share a modular population-level structure, in which each node is composed of one or more interacting populations. This is also the reason why our grammar follows exactly this modular structure as much as possible – the node dynamics is constructed by first choosing the number of populations within the node.

We also agree that comparison only at the level of full equations can obscure an important intermediate level of organization, namely the population-level circuit architecture and that it would be interesting to analyze and discuss more if models with similar population-level architecture behave similarly as well. We interpret the reviewer's term "circuit motif" as broader than the "connectivity motif" used in the grammar. In our terminology, connectivity motif refers specifically to the directed adjacency pattern among populations, whereas the reviewer's point also concerns the number of interacting populations and the resulting population-level architecture.

To analyze this in more detail, we added a targeted supplementary analysis grouping generated models by the number of populations and their connectivity configuration. This analysis was designed as a structural check rather than as a complete empirical comparison of all grammar regions. For each generated model, performance was summarized similarly as described in the Methodology section. Specifically, we used a robust best-hyperparameter IAE metric, computed by first taking the median IAE across repeated optimization runs within each hyperparameter configuration, assigning failed, missing, or non-finite runs a large penalty value, and then selecting the configuration with the lowest penalized median IAE.

The resulting supplementary figure shows that the low-error IAE distributions were broadly overlapping across population--connectivity configurations. We also quantified this observation using Kruskal--Wallis tests. After excluding configurations with fewer than 30 models, the global comparison across all finite model-level scores detected a small statistical departure from identical distributions, but the effect size was negligible (H = 31.40, p = 0.0017, epsilon^2 = 0.0052). Within the low-error regime shown in the supplementary figure (IAE <= 20), there was no evidence of systematic differences across configurations (H = 4.10, p = 0.663, epsilon^2 approximately 0).

These results support the conclusion that population-level circuit architecture is an informative descriptor, but is not by itself sufficient to determine empirical spectral adequacy in the present single-node EEG fitting setting. Models with similar population--connectivity configurations can differ substantially because of local dynamics, input placement, coupling functions,

parameterization, and numerical robustness, while different configurations may yield similar spectral summaries. We have added this clarification to the Discussion and present the population--connectivity analysis in Supplementary Material F.

Specific comments

Line 650: Some authors use the term "next-generation neural mass models" to refer to the MPR model; these are alternative names for the same framework. The apparent differences between Eq. 4 in Ref. [6] and Eqs. 61–62 (SM) arise from a change of variables (see Eq. 18 in Ref. [67]). Moreover, Ref. [6] presents an extended version of Eqs. 61–62 (SM) that includes synaptic kinetics (via two additional ODEs) and conductance-based (instead of current-based) synapses.

Thank you for this clarification. We agree that the terminology in the original manuscript should be more precise and revised the manuscript accordingly. We now refer to MPR also as a next-generation neural mass model and add a clarification that the models not yet included in the present grammar are not the basic MPR formulation itself, but extended next-generation neural mass models that include additional physiological mechanisms, such as explicit synaptic kinetics and conductance-based synapses.

I disagree with the classification of the MPR model as a phenomenological model. The MPR model is unique in that it can be derived exactly from a large network of spiking neurons. Its variables correspond to population firing rate and mean membrane potential, as in classical neural mass models, and it should therefore be regarded as a biologically grounded neural mass model rather than a phenomenological one.

The reviewer is right to point out that the MPR model should not be treated as a phenomenological oscillator. The ambiguity arose because the MPR model combines features that point toward different classification criteria. It has a compact polynomial equation structure similar to some phenomenological models, but it is derived from an underlying spiking-neuron population, as you also point out, and its variables have clear population-level interpretations. We have revised the manuscript to classify MPR as a neural mass / exact mean-field model. We also added a clarification that, although MPR clusters structurally with compact low-dimensional models because of its equation form, its mechanistic interpretation is that of a biologically grounded neural mass model, rather than a phenomenological oscillator. We have found a similar classification also in a recent paper by Hashemi et al., which we also now cite.

Related to the previous points, I recommend that the authors consult the recent review (https://doi.org/10.48550/arXiv.2512.10982), which compares many of the models considered here from a dynamical systems perspective and proposes a classification that may be useful for this study.

We thank the reviewer for pointing us to this recent review. We found it very relevant to the present study, particularly because it organizes the models along a continuum, from low-dimensional oscillator descriptions to more biologically detailed neural mass models. We also found the broader terminology proposed in the review interesting. The term »aggregate neuronal models« can easily encompass models that do not necessarily have an explicit population-level derivation. We have added this reference to the Introduction and revised the text to acknowledge both the proposed terminology and the continuum-based view of their models studied.

Regarding the generated models: do any of them coincide with existing canonical models?

Yes, the grammar is able to reproduce several existing canonical models, which is an important validation of the grammar construction. This confirms that the grammar does not only generate novel architectures, but also contains established models as special cases. We explicitly checked the one million grammar-sampled models against the canonical model recipes and found exact matches for seven canonical models: HO, RRWT, RRWC, JR, WWR, ARM and LB. Interestingly, the best performing canonical models, like MPR and FHN, were not sampled by the grammar. We also note that all canonical models were evaluated independently as reference models throughout the study, so exact matches in the grammar sample were not treated as additional generated-model evaluations. We have clarified this point and added information on which canonical models were recovered exactly in the grammar-sampled set.

Line 64: The authors state that the generated models are biologically plausible alternatives to canonical models. This claim requires clarification. For instance, the model defined by Eqs. (5–6), as well as models G1, G4, and G9, are planar polynomial systems with a fairly general structure. In the absence of a clear interpretation of variables and parameters, it is not evident why these models should be considered biologically plausible. They appear closer to abstract phenomenological models (e.g., Stuart–Landau or harmonic oscillator models) than to biologically motivated ones.

We thank the reviewer for this important comment. We agree that the phrase "biologically plausible alternatives" was too broad in the original manuscript, especially when applied to all grammar-generated models. The grammar used in this study was intentionally constructed from both mechanistic neural mass components and phenomenological dynamical-system components. Consequently, not every sampled model should be interpreted as biologically plausible in the strict mechanistic sense.

We have modified the manuscript to avoid implying that all generated models are biologically plausible alternatives to canonical neural mass models. Instead, we describe the grammar as generating candidate neural population model structures whose degree of biological interpretability depends on the selected grammar components. If the grammar were restricted to neural mass components only, the generated models would be more directly biologically interpretable; in the present broader grammar, however, the model space includes all types of neural population models from the literature, thus both mechanistic neural mass-like formulations and more abstract phenomenological alternatives.

The best-performing generated model (Eqs. 5–6) appears to be structurally similar to the MPR model (albeit missing a quadratic term), which is also the best-performing canonical model. This is an interesting result. Do the authors have an interpretation for this similarity?

This similarity is indeed interesting and suggests that compact, low-dimensional population-level dynamics may be especially effective for these spectra. Both models are compact two-dimensional polynomial systems and share several structural elements, including constant terms and low-order quadratic interactions between the two state variables. In particular, the generated model contains a bilinear interaction term in the first equation and a quadratic term in the second equation, which makes it structurally close to the broad class of quadratic planar systems to which the MPR equations also belong.

We do not, however, interpret this as evidence that the generated model recovers the MPR mechanism. The MPR model has a specific derivation as an exact mean-field reduction of a network of quadratic integrate-and-fire neurons, and its variables have clear interpretations as population

firing rate and mean membrane potential. By contrast, the generated model does not impose these variable interpretations or the coefficient constraints of the MPR formulation, and it lacks one of the quadratic terms present in MPR. We therefore interpret the similarity more cautiously: it suggests that compact two-dimensional quadratic dynamics may be particularly effective for reproducing the spectral features considered in this study, but it does not by itself establish a mechanistic equivalence with MPR. We have added this clarification to the manuscript.

The manuscript would benefit from a clearer discussion of how the generated models relate to the canonical ones, particularly in terms of structural similarities and differences.

We appreciate the reviewer's suggestion. The relationship between generated and canonical models is central to the construction of the grammar, as the grammar was derived from structural components of the canonical models, and generated models were positioned relative to canonical families using the same equation-level structural distance used for the canonical clustering. In particular, Fig. 6 already shows grammar-generated models in a complexity–distance space, where each model is assigned to the closest canonical cluster.

However, we agree that the interpretation of this relationship could be made clearer in the text. We have therefore revised the Results and Discussion to state more explicitly that the evaluated generated models are not exact rediscoveries of canonical models, but canonical-like variants within the compact low-dimensional structural family. They share broad features with canonical models such as MPR, FHN, and SL, including a small state dimension and low-order polynomial interactions, but differ in the specific placement of nonlinear terms, input terms, and coefficient constraints. We also clarify that the present generated-model analysis represents a local exploration around the best-performing canonical structural family, rather than an exhaustive search across all canonical clusters. To make this relationship more concrete, we have also added a dedicated comparison of G1 and MPR in the Results, highlighting both their shared compact two-dimensional polynomial structure and their differences in the placement of nonlinear and input terms.

Minor issues / typos:

Supplementary Material, after Eq. 71: Table 1 is incorrectly referenced or labeled.

We thank the reviewer for the careful inspection. We have corrected this typo in the Supplementary Material and replaced it with an explicit reference to "Table 1 in the main manuscript".

Reviewer #2:

The authors introduce a timely and useful framework, ENEEGMA, which systematically compares canonical neural mass and phenomenological models while using grammar-based equation discovery to explore novel architectures. The work is significant for the field of computational neuroscience, and whole-brain modeling in particular, as it addresses the often-overlooked lack of systematic guidance on how canonical formulations differ in their empirical adequacy for EEG data. The use of a probabilistic grammar to assemble interpretable dynamical components is a principled move toward more transparent "automated modeling".

The paper is well written and technically sound, I would just really prefer having the figures in the text, it is very difficult to follow the work in the current format.

We thank the reviewer for the positive assessment of the manuscript and for recognizing the relevance of our work for computational neuroscience and whole-brain modelling. We also agree that placing the figures within the main text would make the manuscript easier to follow. In the initial submission, we followed the PLOS formatting guidance. To improve readability and facilitate the review process, we now provide an additional version of the manuscript in which the figures are embedded directly within the text. The file is called »Manuscript with figures embedded.pdf«.

The Introduction would benefit from a broader positioning within the recent literature on implicit dynamical systems reconstruction and data-driven brain network modeling, such as the works by Sip et al. (2023) Science Advances, 2023. Similarly, regarding SINDy, it was recently applied to recover the effective connectivity in a whole brain (Kashyap et al Nat Commun 2025) so maybe it is also worth mentioning.

Thank you for pointing us to these relevant recent studies. Because our work focuses on EEG-based neural population models, we had not sufficiently covered related developments in fMRI-based dynamical systems reconstruction and data-driven whole-brain network modelling. We have revised the Introduction to include the suggested recent work.

It is not fully clear to me how the working points are obtained for the comparisons, and whether Fig. 7 for example shows values for different parameter values within some assigned range?

By “working points,” we understand the fitted parameter values and initial conditions used for the final simulations. For the comparison figures, including Figures 3 and 7 as well as Table 3, these values were not sampled arbitrarily from the assigned parameter ranges. Instead, for each model, parameters were first optimized using the spectral objective function, with five optimization runs per model, subject, and task. For the final evaluation shown in the figures, the best-fitting parameter set from these runs was fixed, and the model was then re-simulated five times with different random seeds, corresponding to different initial conditions and noise realizations. The summary figures therefore reflect variability across these repeated simulations and across the four datasets/subjects included in each condition, while using the best-performing parameter set selected from the optimization runs.

We agree this point was not sufficiently clear in the previous version of the manuscript. We have now clarified the fitting and evaluation workflow in the Methods and figure captions.

Same question about the value of the noise, how is it chosen across the models, and in which variables it enters?

After re-examining the manuscript, we found that the description of noise handling was distributed across several parts of the Methods and was therefore not sufficiently clear. We have added a dedicated "Noise handling" paragraph to the Empirical evaluation section of Materials and methods. This paragraph distinguishes between three sources of noise: external input noise, internal stochasticity of the model, and additive measurement noise. It also specifies where these terms enter the equations and how their amplitudes are treated during fitting.

More important concern I have regarding the comparison of PSD fits in Figures 4 and 8. According to Table 1, the MPR model is categorized as having $N_{pop}=1$, but I find it surprising that a single-population model like MPR can exhibit multiple distinct peaks in the spectrum (as seen in Fig 4). The authors should clarify how is this achieved. In this respect it would help if at least in the SM there is a figure with the time series of each model.

We thank the reviewer for raising this important point. We think the reason for the multiple spectral peaks in a one-population model such as MPR in the SSVEP condition, is due to the external drive used in the simulations, which was a pure sinusoid at the stimulation frequency, which did not itself contain harmonic components. However, this sinusoidal drive enters nonlinear model equations. Therefore, harmonics can be generated by the model response through the interaction between periodic forcing, the fitted operating regime, and nonlinear state dynamics.

We have clarified this point in the Results and added Supplementary Material H, which shows example simulated time series of the MPR model, with and without sinusoid input.

We have also added additional plots of the time series of each model. For resting-state data, several of the best-fitting models produce simulated time series that qualitatively resemble the empirical signal, despite the fact that the optimization was performed in spectral space rather than directly on the time series. In contrast, for the SSVEP condition, good harmonic-restricted PSD fits do not necessarily imply close time-domain resemblance. This likely reflects the fact that the SSVEP simulations were driven without noise (the input was only pure sinusoid), whereas resting-state simulations included random noise as input. Another reason for the low resemblance could be the SSVEP objective emphasized stimulus-locked harmonic regions rather than full time-domain waveform matching.

In the manuscript, we have added a discussion of this limitation. In future work, SSVEP fitting could be improved by including more realistic stochastic background drive in addition to the periodic stimulus, and by using objectives that jointly constrain harmonic structure, broadband spectral background, and time-domain features. We also added the code used to generate the results and figures to the GitHub repository, making it easier to inspect the fitted simulations and produce time-series plots for all models.

I also find it peculiar that models with $N_{pop}=1$ (MPR, FHN, SL) achieved the strongest overall rankings. The authors should discuss whether the poorer performance of multi-population models like MDF is due to optimization challenges in higher-dimensional parameter spaces.

Similarly, the manuscript reports a startlingly low success rate for some canonical models, such as the LW at 33.33%. The authors should discuss whether this instability is an inherent property of the

model's stiff dynamics or a failure of the parameter optimization bounds chosen for the CMA-ES algorithm. Given that numerical stability is a "practical dimension of model adequacy," this could be emphasized more strongly as a criterion for model selection in TVB-like frameworks.

Yes, we agree that the lower performance of multi-population or higher-dimensional models should not be interpreted solely as evidence of weaker biological adequacy or be a sign of a poor model. To address this, we added an exploratory analysis relating fitting error and fitting success to model complexity and parameterization scale. Specifically, we examined the number of fitted parameters, the number of state equations, and the aggregate magnitude of literature/default parameter values.

The additional analysis is provided in Supplementary Material G. This analysis showed that final fitting error among successful fits was only weakly related to the number of parameters or equations. In contrast, fitting success rate was more clearly associated with model complexity and, most strongly, with the magnitude of aggregated default parameter values. We interpret the latter cautiously, because parameters have different units and the aggregate scale is not a physically meaningful complexity measure. Nevertheless, it provides a useful exploratory proxy for parameter-scale heterogeneity: models with larger gains, faster rates, steeper nonlinearities, or conductance-like parameters may more easily enter sensitive, unstable, or stiff regimes during optimization.

We have therefore also revised the Discussion to clarify that poorer performance and lower success rates of models such as MDF and LW may reflect a combination of optimization difficulty, numerical fragility, parameter-bound choices, and mismatch between the model's intended physiological regime and the present single-node PSD objective. We also now emphasize fitting success and numerical robustness as practical dimensions of model adequacy, particularly relevant for TVB-like workflows where models must be repeatedly simulated and fitted across many regions, subjects, and conditions.

Regarding the more complex models that are generated, would extensions of those 17 canonical models fell within them? For example the MPR variance with excitatory and inhibitory population, or any of its applications as a "novel neuronal mass" as in the recent Forrester et al Plos CB 2024?

We agree that this is an important question, but we did not directly investigate whether extensions of the 17 canonical models fall within the more complex regions of the generated model space. In principle, some extensions of canonical models would be partly representable by the current grammar, especially when they can be decomposed into the same broad components: interacting populations, input processes, coupling functions, connectivity motifs, and stochasticity that are present in the grammar. However, next-generation neural mass models such as the excitatory–inhibitory MPR/QIF-based formulation used by Forrester et al. include additional structure that is not fully represented in the current grammar, including conductance-based synaptic interactions with reversal potentials, electrical/gap-junction coupling, and explicit synchrony/order-parameter dynamics. These models therefore represent a natural and important direction for extending the grammar, but they were not part of the present analysis. We have added this point to the limitations and future-work discussion.

Maybe the authors should show the exact form of the few of the 1000 models studied in Fig. 6?

We thank the reviewer for this suggestion. We agree that the generated models should be inspectable at the equation level. To avoid overloading the main manuscript with many additional equations, we now provide the exact forms of representative generated models discussed in the Results, and we have made the full set of evaluated generated-model specifications available in the GitHub repository.

Specifically, the repository includes a CSV file containing the terminal-symbol sequences for all 1,000 evaluated generated models, that is, the structural specifications sampled from the grammar. ENEEGMA also includes the code that transforms these grammar specifications into full systems of differential equations. Thus, the exact equations of every evaluated generated model can be reconstructed, inspected, and reproduced.

I'm wondering how would the authors implement the optimization if there were more than one metric, let's say some connectivity metric, in addition to the PSD? Is there a principled way the fit to be performed over a set of parameters?

Yes, we fully agree that fitting only PSD-based summaries can leave substantial underdetermination, and that additional observables, such as functional connectivity, phase coherence, cross-spectral measures, or other network-level statistics, would provide stronger constraints on the model.

In principle, such metrics can be incorporated by extending the objective function with additional discrepancy terms. This is conceptually similar to the loss used in the present study, where different spectral components are combined using task-specific weights. In a multi-observable setting, one could define a composite objective that combines normalized errors across observables, for example a PSD error term together with a functional-connectivity error term. The corresponding weights could be fixed based on the scientific question, selected as hyperparameters, or tuned using validation data, so that one metric does not dominate only because of its scale.

A second principled option would be to treat the problem explicitly as multi-objective optimization. Instead of reducing all observables to a single weighted scalar loss, one could retain a Pareto set of parameter vectors representing different optimal trade-offs between PSD fit, connectivity fit, and other summary statistics. This would be particularly useful when no single weighting of observables is clearly justified.

In the present manuscript, we focused on single-node spectral fitting, so network-level observables such as functional connectivity were outside the scope of the analysis. However, we have now added this point to the Discussion. The modular implementation of ENEEGMA should make it straightforward to add new objective-function terms in future whole-brain extensions, and we view multi-observable and Pareto-based fitting as important next steps for reducing model underdetermination.

Minor Points

- Author Summary: The summary is clear, but could emphasize the Julia implementation's performance benefits for large-scale grammar sampling.

We have taken this suggestion into account and revised the Author Summary to better highlight the computational role of the Julia implementation, which especially supports efficient simulation of differential equations. We phrase this cautiously as a design motivation rather than as a formal performance claim, since we did not perform systematic benchmarking or compare computational costs across programming languages or implementations.

- SSVEP Ranking: The "Generated (G1)" cluster actually achieved the strongest Bayesian expected rank $(E[rank]=1.0)$ for the SSVEP task (Table 5), outperforming all canonical clusters. This is a major highlight that deserves more emphasis in the Abstract.

Thank you also for this suggestion. Although the previous abstract already stated that the generated models achieved the strongest fits on the SSVEP task, it is true we did not specifically highlight the statistical ranking. We have revised it accordingly.

-Model Complexity: The complexity analysis in Figure 5 shows that grammar-generated models (GEN) tend to have more parameters than state equations compared to canonical models. A brief comment on whether this leads to overfitting risks would be insightful.

Yes, this is an important point. We checked this explicitly and found that approximately 81.6% of the one million sampled models still fall within the parameter-count range spanned by the canonical models. Although the generated distribution has a right tail toward models with more parameters (which is expected as is bounded with zero on the left side), the number of such models decreases rapidly with increasing parameter count. We therefore think that the grammar samples the model space in a reasonably controlled way, rather than simply favoring increasingly complex models.

We have nevertheless clarified that the grammar-generated model space should not be interpreted as a set of equally admissible models for empirical fitting. Model complexity, as well as other potential features of the models, can be calculated and controlled explicitly during sampling or model selection. For example, during sampling, the recursive polynomial construction can be strongly restricted by reducing the probability of recursive production rules, thereby biasing the grammar toward simpler equations. Additional constraints can also be imposed on the number of populations, parameters, state variables, or connectivity motifs. In the present study, we used a post-sampling selection strategy by evaluating models from the lower-left, low-complexity region of the structural space. Thus, while overfitting is a potential concern in principle for any sufficiently flexible model-generation framework, there are several straightforward mechanisms for controlling this risk.

- a more recent TVB review by Hashemi et al. 2025 IEEE could be cited.

We thank the reviewer for pointing us to this recent review. In the original manuscript, we cited the foundational TVB references and more recent EBRAINS implementation work. We agree that citing a recent review is also useful for providing readers with an updated overview of the TVB framework. Also, they do have a figure with separating models between two main clusters, biopyhisical and phenomenological, which we found very informative and related to our classification. We have therefore added the recommended reference to the Introduction, where TVB is first discussed as one of the established platforms for large-scale brain network modelling.

- since MPR seems to show the best performance, maybe some of its applications in modeling with TVB should be mentioned.

We appreciate the suggestion. While a detailed review of TVB applications of the MPR model is outside the scope of the present study, we agree that, given the strong performance of MPR in our comparison, it is useful to place this result in the context of recent whole-brain modelling applications. We examined the relevant TVB publication list to assess this point, however, the original two-variable MPR formulation evaluated in our study appears less commonly used in TVB applications than extended MPR -based formulations. In the manuscript, we now mention recent work by Forrester et al., where the extended formulation of MPR is used.
