# Supplementary material for: Neural population models for EEG: From Canonical models to alternative model structures
Source: PLoS Comput Biol. 2026 Aug 6;22(8):e1014222. doi: 10.1371/journal.pcbi.1014222 (PMC13460749; doi:10.1371/journal.pcbi.1014222)
Supplement: Supplementary file 11 [file pcbi.1014222.s012.pdf]

Dear Reviewers,

Thank you again for taking time to review our manuscript. Please find our responses to your comments below. We first provide your text, point by point, and after each point, we provide our response, coloured in blue.

Reviewer #1: The authors have satisfactorily addressed all of my comments and questions, as well as those of the other reviewer, and have revised the manuscript accordingly. I recommend the paper for publication in PLOS Computational Biology.

We thank the reviewer for their time, careful assessment, and valuable feedback. We greatly appreciate their recommendation for publication.

Reviewer #2: The authors have responded to most of my comments. However. I would still require some clarifications.

After the responses about the working point and the noise, now I understand better that the way the models are parametrized for the resting state here seems somewhat different from the way it is done when these neural mass models are employed for whole brain models.
When brain network models are constructed, the initial conditions and noise seeds (the noise is always part of the generative system) are barely of any relevance. I expect this to be the case also for the PSD as a feature for the single node here, given that the initial transient of any model is taken out, the results should be insensitive on the initial conditions and the noise seeds. The only way when the noise can play a role, is if it gets different statistics over time (its strength becomes time-varying as for example in the case of Sip et al, Sci. Adv. 2021). However, every model is very sensitive on the working point as defined by its parameters, that define its dynamical regime. I think this merits additional discussion. For example the most successful model here, MPR, when used in TVB for the resting state (Lavanga et al. Neuroimage 2023, Fousek et al. Scientific Reports 2024, Breyton et al eLife 2025), it is always in its bistable regime, where the required switching dynamics is driven by the noise and the stimuli from the other regions.
So to be more specific, is the strength of the white noise added to the rs simulations evaluated, and how was it chosen for different models?

First, we thank the reviewer for these relevant comments and clarifications. We agree that the fitted working point is a primary determinant of a model’s dynamical regime and that our previous description did not sufficiently distinguish the different sources of variability.

In our final evaluations, all optimized parameters, including the sensory-input gain (strength), were held fixed. Repetitions differed in their initial states. Resting-state simulations additionally used different realizations of the external sensory input, a normally distributed Gaussian noise, whereas SSVEP simulations used the same deterministic sinusoidal input. The amplitude and realization of the measurement noise added before PSD estimation were held fixed across repetitions. Consequently, variation among deterministic SSVEP simulations cannot be attributed to changes in input noise, measurement noise, or sensory-input strength.

Inspection of the repeated evaluations showed that some of the largest apparent differences were associated with unsuccessful simulations from particular initial states, which returned an all-zero PSD and a non-finite error. These cases were treated as numerical failures rather than as valid alternative dynamical outcomes. Within these cases, the successful repetitions were considerably more similar. To investigate the remaining variability directly, we performed an additional diagnostic

using representative MPR and WC SSVEP fits. Repeated simulations using identical fitted parameters and the optimized initial state produced exactly identical PSDs, confirming deterministic reproducibility. Simulations using different initial states reproduced the observed PSD variation. Thus, for these representative fits, the remaining differences are due to sensitivity to the initial state and the finite analyzed time window.

To clarify how external sensory input was handled across models: In the resting-state simulations, the raw external input consisted of independent samples drawn from a standard normal distribution, N(0,1). The same raw input distribution was used for all models. However, because the models differ in state-variable scaling, units, and input implementation, the sensory-input gain was fitted separately for each model and dataset. This allowed the effective input to adapt to the scaling of each model. Although, we acknowledge that fixing the effective input strength would provide a stricter comparison of intrinsic model dynamics.

We have revised the Methods sections *Empirical evaluation of canonical models*, specifically the subsections *External drive* (page 7, lines 212–224) and *Noise handling* (page 7, lines 225–241), to distinguish the standardized condition-specific input, the fitted sensory-input gain, internal stochasticity, and additive measurement noise. We also expanded the *Parameter estimation* (pages 8–9, lines 256–308) and *Evaluation* (page 9, lines 309–329) subsections to clarify the simulation and transient-removal procedure, and which quantities were held fixed or varied across repeated evaluations.

And second, for one specific model, say MPR, to which dynamical regime do correspond the parameters chosen in the optimization, in respect to the Fig. 1a in Montbrio et al PRX 2015?

We thank the reviewer for this question, which prompted us to clarify an important aspect of our procedure. In our implementation, the parameters of the model equations were optimized independently, and the parameter identities of the native MPR formulation were not enforced. Consequently, the fitted parameters cannot be directly assigned a unique point in the native MPR parameter plane presented by Montbrió et al. (2015). We have now clarified throughout the manuscript that the evaluated models are generalized fitted implementations of the canonical architectures rather than native parameterizations, specifically, in Method's subsection *Canonical neural population models* (page 5, lines 151–163) and in Result's subsection Empirical analysis of canonical models (page 14, lines 470-474).

Still, to answer the reviewer's question as directly as possible, we characterized the fitted dynamics directly from our fitted MPR model's fixed points and linear stability. The representative RS and SSVEP fits both exhibited a low-activity stable node and a high-activity stable focus separated by a saddle. The fitted generalized MPR models therefore showed a bistable fixed-point topology qualitatively analogous to the native MPR bistable regime. However, because the native parameter relationships were not satisfied, the fitted models cannot be assigned exact coordinates in the parameter plane of Fig. 1a in Montbrio (2015).

Additional analysis of the dynamics is now reported in the Results section, under Empirical analysis of canonical models (page 17, lines 538–549), and discussed in the Discussion, under »Among the canonical models, low-dimensional polynomial oscillators combine structural simplicity with the best single-node EEG spectral fits« (page 23, lines 716–720). The relationship between native parameters and our generalized parameters and phase-plane figures are provided in S9 Appendix (Supplementary Materials, pages 30–31).

For completeness, we then also included the phase plane analysis of G1, which is located in the S9 Appendix as well.

Additional correction made during revision.

During preparation of this revision, we also identified an error affecting the RS results of the JR model for one dataset. We corrected the affected evaluation rows and repeated all downstream descriptive and Bayesian analyses. Consequently, Figures 3, 4, and 7 and Tables 3 and 5 were updated. The correction changed several JR-specific summary values and rankings but did not alter the principal conclusions: the generalized MPR architecture remained the best-performing canonical model, followed by FHN and SL among the leading low-dimensional models.
