# Supplementary material for: Neural population models for EEG: From Canonical models to alternative model structures
Source: PLoS Comput Biol. 2026 Aug 6;22(8):e1014222. doi: 10.1371/journal.pcbi.1014222 (PMC13460749; doi:10.1371/journal.pcbi.1014222)
Supplement: Peer review history

# Peer review: Decision Letter 0

**DOI:** 10.1371/journal.pcbi.1014222.r001

**Authors:** Emili Balaguer-Ballester, Daniele Marinazzo

8 May 2026

PCOMPBIOL-D-26-00834

Neural Population Models for EEG: From Canonical Models to Alternative Model Structures

PLOS Computational Biology

Dear Dr. Omejc,

Thank you for submitting your manuscript to PLOS Computational Biology. After careful consideration, we feel that it has merit but does not fully meet PLOS Computational Biology's publication criteria as it currently stands. Therefore, we invite you to submit a revised version of the manuscript that addresses the points raised during the review process.

Please submit your revised manuscript by Jul 08 2026 11:59PM. If you will need more time than this to complete your revisions, please reply to this message or contact the journal office at ploscompbiol@plos.org. When you're ready to submit your revision, log on to https://www.editorialmanager.com/pcompbiol/ and select the 'Submissions Needing Revision' folder to locate your manuscript file.

Please include the following items when submitting your revised manuscript:

* A letter that responds to each point raised by the editor and reviewer(s). You should upload this letter as a separate file labeled 'Response to Reviewers'. This file does not need to include responses to formatting updates and technical items listed in the 'Journal Requirements' section below.

* A marked-up copy of your manuscript that highlights changes made to the original version. You should upload this as a separate file labeled 'Revised Manuscript with Track Changes'.

* An unmarked version of your revised paper without tracked changes. You should upload this as a separate file labeled 'Manuscript'.

If you would like to make changes to your financial disclosure, competing interests statement, or data availability statement, please make these updates within the submission form at the time of resubmission. Guidelines for resubmitting your figure files are available below the reviewer comments at the end of this letter.

As the corresponding author, your ORCID iD is verified in the submission system and will appear in the published article. PLOS supports the use of ORCID, and we encourage all coauthors to register for an ORCID iD and use it as well. Please encourage your coauthors to verify their ORCID iD within the submission system before final acceptance, as unverified ORCID iDs will not appear in the published article. Only the individual author can complete the verification step; PLOS staff cannot verify ORCID iDs on behalf of authors.

We look forward to receiving your revised manuscript.

Kind regards,

Emili Balaguer-Ballester, PhD

Academic Editor

PLOS Computational Biology

Daniele Marinazzo

Section Editor

PLOS Computational Biology

**Journal Requirements:**

1) We ask that a manuscript source file is provided at Revision. Please upload your manuscript file as a .doc, .docx, .rtf or .tex. If you are providing a .tex file, please upload it under the item type u2018LaTeX Source Fileu2019 and leave your .pdf version as the item type u2018Manuscriptu2019.

2) Please upload all main figures as separate Figure files in .tif or .eps format. For more information about how to convert and format your figure files please see our guidelines:

https://journals.plos.org/ploscompbiol/s/figures

3) We have noticed that you have uploaded Supporting Information files, but you have not included a list of legends. Please add a full list of legends for your Supporting Information files after the references list.

4) Please amend your detailed Financial Disclosure statement. This is published with the article. It must therefore be completed in full sentences and contain the exact wording you wish to be published.

- State the initials, alongside each funding source, of each author to receive each grant. For example: "This work was supported by the National Institutes of Health (####### to AM; ###### to CJ) and the National Science Foundation (###### to AM)."

- State what role the funders took in the study. If the funders had no role in your study, please state: "The funders had no role in study design, data collection and analysis, decision to publish, or preparation of the manuscript.".

If you did not receive any funding for this study, please simply state: u201cThe authors received no specific funding for this work.u201d

**Reviewers' comments:**

Reviewer's Responses to Questions

**Comments to the Authors:**

**Please note here if the review is uploaded as an attachment.**

Reviewer #1: This manuscript presents a systematic comparison of 17 canonical models currently used in neuroscience to analyze EEG recordings. The authors employ a Julia-based computational framework they developed, ENEEGMA (Exploring Neural EEG Model Architectures), to compare these models in terms of both mathematical structure and empirical performance. In addition, ENEEGMA is used to generate alternative model architectures with potentially improved performance. The main contribution of the work is thus twofold: the development of the ENEEGMA framework and the results of the comparative analysis. The evaluation is conducted on four EEG datasets under two conditions (resting state and visual-evoked responses).

I appreciate the authors’ effort to systematically compare neural mass models based on both their mathematical properties and their empirical performance. Although parts of the manuscript describing the design of ENEEGMA draw heavily on machine learning concepts—which are outside my area of expertise and occasionally difficult to follow, I believe the results will be of interest to researchers in computational and mathematical neuroscience. In particular, the study is relevant for those developing and analyzing neural mass models, as well as for researchers focused on EEG data modeling.

The generation of new model architectures is particularly interesting, as it may provide insight into the key mathematical ingredients required to reproduce EEG data. That said, these generated models may or may not be directly related to biophysical mechanisms and may instead fall into the category of phenomenological models. On the other hand, the comparative evaluation of established models is likely to be useful for researchers selecting appropriate modeling frameworks for EEG analysis.

Overall, I am positively inclined to recommend this manuscript for publication in PLOS Computational Biology, subject to minor revisions. However, I have several questions and suggestions that should be addressed before publication.

------------------------------

General comment:

I have a general comment regarding the choice and comparison of the canonical models. To clarify my point, consider the example of the Wilson–Cowan (WC) model. This model describes a canonical neuronal circuit consisting of interacting excitatory (E) and inhibitory (I) populations, each represented by a single ODE describing its activity. This modular structure is shared by many (if not all) neural mass models considered in the manuscript, including the MPR model.

It would be valuable to clarify whether the results and conclusions of the study remain consistent when comparing models built from the same underlying circuit motif (e.g., a single recurrent population or an E–I pair), but implemented using different neural mass formulations. I encourage the authors to discuss this point and, if feasible, to include an additional analysis comparing models at this level.

Such a comparison could be particularly relevant for mechanistic interpretations of EEG activity, linking observed dynamics to underlying circuit motifs (e.g., E–I-driven synchronization, excitatory persistent activity, or inhibitory-driven synchrony). Naturally, this type of comparison would be restricted to neural mass models.

---------------------------------

Specific comments

-Line 650: Some authors use the term “next-generation neural mass models” to refer to the MPR model; these are alternative names for the same framework. The apparent differences between Eq. 4 in Ref. [6] and Eqs. 61–62 (SM) arise from a change of variables (see Eq. 18 in Ref. [67]). Moreover, Ref. [6] presents an extended version of Eqs. 61–62 (SM) that includes synaptic kinetics (via two additional ODEs) and conductance-based (instead of current-based) synapses.

-I disagree with the classification of the MPR model as a phenomenological model. The MPR model is unique in that it can be derived exactly from a large network of spiking neurons. Its variables correspond to population firing rate and mean membrane potential, as in classical neural mass models, and it should therefore be regarded as a biologically grounded neural mass model rather than a phenomenological one.

- Related to the previous points, I recommend that the authors consult the recent review (https://doi.org/10.48550/arXiv.2512.10982), which compares many of the models considered here from a dynamical systems perspective and proposes a classification that may be useful for this study.

- Regarding the generated models: do any of them coincide with existing canonical models?

- Line 64: The authors state that the generated models are biologically plausible alternatives to canonical models. This claim requires clarification. For instance, the model defined by Eqs. (5–6), as well as models G1, G4, and G9, are planar polynomial systems with a fairly general structure. In the absence of a clear interpretation of variables and parameters, it is not evident why these models should be considered biologically plausible. They appear closer to abstract phenomenological models (e.g., Stuart–Landau or harmonic oscillator models) than to biologically motivated ones.

-The best-performing generated model (Eqs. 5–6) appears to be structurally similar to the MPR model (albeit missing a quadratic term), which is also the best-performing canonical model. This is an interesting result. Do the authors have an interpretation for this similarity?

-The manuscript would benefit from a clearer discussion of how the generated models relate to the canonical ones, particularly in terms of structural similarities and differences.

Minor issues / typos:

- Supplementary Material, after Eq. 71: Table 1 is incorrectly referenced or labeled.

Reviewer #2: The authors introduce a timely and useful framework, ENEEGMA, which systematically compares canonical neural mass and phenomenological models while using grammar-based equation discovery to explore novel architectures. The work is significant for the field of computational neuroscience, and whole-brain modeling in particular, as it addresses the often-overlooked lack of systematic guidance on how canonical formulations differ in their empirical adequacy for EEG data. The use of a probabilistic grammar to assemble interpretable dynamical components is a principled move toward more transparent "automated modeling".

The paper is well written and technically sound, I would just really prefer having the figures in the text, it is very difficult to follow the work in the current format.

The Introduction would benefit from a broader positioning within the recent literature on implicit dynamical systems reconstruction and data-driven brain network modeling, such as the works by Sip et al. (2023) Science Advances, 2023. Similarly, regarding SINDy, it was recently applied to recover the effective connectivity in a whole brain (Kashyap et al Nat Commun 2025) so maybe it is also worth mentioning.

It is not fully clear to me how the working points are obtained for the comparisons, and whether Fig. 7 for example shows values for different parameter values within some assigned range? Same question about the value of the noise, how is it chosen across the models, and in which variables it enters?

More important concern I have regarding the comparison of PSD fits in Figures 4 and 8. According to Table 1, the MPR model is categorized as having $N_{pop}=1$, but I find it surprising that a single-population model like MPR can exhibit multiple distinct peaks in the spectrum (as seen in Fig 4). The authors should clarify how is this achieved.

In this respect it would help if at least in the SM there is a figure with the time series of each model.

I also find it peculiar that models with $N_{pop}=1$ (MPR, FHN, SL) achieved the strongest overall rankings.

The authors should discuss whether the poorer performance of multi-population models like MDF is due to optimization challenges in higher-dimensional parameter spaces.

Similarly, the manuscript reports a startlingly low success rate for some canonical models, such as the LW at 33.33%.

The authors should discuss whether this instability is an inherent property of the model’s stiff dynamics or a failure of the parameter optimization bounds chosen for the CMA-ES algorithm. Given that numerical stability is a "practical dimension of model adequacy," this could be emphasized more strongly as a criterion for model selection in TVB-like frameworks.

Regarding the more complex models that are generated, would extensions of those 17 canonical models fell within them? For example the MPR variance with excitatory and inhibitory population, or any of its applications as a “novel neuronal mass” as in the recent Forrester et al Plos CB 2024?

Maybe the authors should show the exact form of the few of the 1000 models studied in Fig. 6?

I’m wondering how would the authors implement the optimization if there were more than one metric, let’s say some connectivity metric, in addition to the PSD? Is there a principled way the fit to be performed over a set of parameters?

Minor Points

- Author Summary: The summary is clear, but could emphasize the Julia implementation's performance benefits for large-scale grammar sampling.

- SSVEP Ranking: The "Generated (G1)" cluster actually achieved the strongest Bayesian expected rank $(E[rank]=1.0)$ for the SSVEP task (Table 5), outperforming all canonical clusters. This is a major highlight that deserves more emphasis in the Abstract.

- Model Complexity: The complexity analysis in Figure 5 shows that grammar-generated models (GEN) tend to have more parameters than state equations compared to canonical models. A brief comment on whether this leads to overfitting risks would be insightful.

- a more recent TVB review by Hashemi et al. 2025 IEEE could be cited.

- since MPR seems to show the best performance, maybe some of its applications in modeling with TVB should be mentioned.

**********

**Have the authors made all data and (if applicable) computational code underlying the findings in their manuscript fully available?**

The PLOS Data policy requires authors to make all data and code underlying the findings described in their manuscript fully available without restriction, with rare exception (please refer to the Data Availability Statement in the manuscript PDF file). The data and code should be provided as part of the manuscript or its supporting information, or deposited to a public repository. For example, in addition to summary statistics, the data points behind means, medians and variance measures should be available. If there are restrictions on publicly sharing data or code —e.g. participant privacy or use of data from a third party—those must be specified.

Reviewer #1: Yes

Reviewer #2: None

**********

PLOS authors have the option to publish the peer review history of their article (what does this mean?). If published, this will include your full peer review and any attached files.

If you choose “no”, your identity will remain anonymous but your review may still be made public.

**Do you want your identity to be public for this peer review?**  For information about this choice, including consent withdrawal, please see our Privacy Policy.

Reviewer #1: No

Reviewer #2: No

[NOTE: If reviewer comments were submitted as an attachment file, they will be attached to this email and accessible via the submission site. Please log into your account, locate the manuscript record, and check for the action link "View Attachments". If this link does not appear, there are no attachment files.]

**Figure resubmission:**

-->While revising your submission, we strongly recommend that you use PLOS’s NAAS tool (https://ngplosjournals.pagemajik.ai/artanalysis) to test your figure files. NAAS can convert your figure files to the TIFF file type and meet basic requirements (such as print size, resolution), or provide you with a report on issues that do not meet our requirements and that NAAS cannot fix.

After uploading your figures to PLOS’s NAAS tool - https://ngplosjournals.pagemajik.ai/artanalysis, NAAS will process the files provided and display the results in the "Uploaded Files" section of the page as the processing is complete. If the uploaded figures meet our requirements (or NAAS is able to fix the files to meet our requirements), the figure will be marked as "fixed" above. If NAAS is unable to fix the files, a red "failed" label will appear above. When NAAS has confirmed that the figure files meet our requirements, please download the file via the download option, and include these NAAS processed figure files when submitting your revised manuscript.

**Reproducibility:**

To enhance the reproducibility of your results, we recommend that authors of applicable studies deposit laboratory protocols in protocols.io, where a protocol can be assigned its own identifier (DOI) such that it can be cited independently in the future. Additionally, PLOS ONE offers an option to publish peer-reviewed clinical study protocols. Read more information on sharing protocols at https://plos.org/protocols?utm_medium=editorial-email&utm_source=authorletters&utm_campaign=protocols

# Author comment: Author response to Decision Letter 1

**DOI:** 10.1371/journal.pcbi.1014222.r002

24 Jun 2026

Attachment Submitted filename: Response to Reviewers.pdf

# Peer review: Decision Letter 1

**DOI:** 10.1371/journal.pcbi.1014222.r003

**Authors:** Emili Balaguer-Ballester, Daniele Marinazzo

17 Jul 2026

PCOMPBIOL-D-26-00834R1

Neural Population Models for EEG: From Canonical Models to Alternative Model Structures

PLOS Computational Biology

Dear Dr. Omejc,

Thank you for submitting your manuscript to PLOS Computational Biology. After careful consideration, we feel that it has merit but does not fully meet PLOS Computational Biology's publication criteria as it currently stands. Therefore, we invite you to submit a revised version of the manuscript that addresses the points raised during the review process.

Please submit your revised manuscript by Sep 16 2026 11:59PM. If you will need more time than this to complete your revisions, please reply to this message or contact the journal office at ploscompbiol@plos.org. When you're ready to submit your revision, log on to https://www.editorialmanager.com/pcompbiol/ and select the 'Submissions Needing Revision' folder to locate your manuscript file.

Please include the following items when submitting your revised manuscript:

* A letter that responds to each point raised by the editor and reviewer(s). You should upload this letter as a separate file labeled 'Response to Reviewers'. This file does not need to include responses to formatting updates and technical items listed in the 'Journal Requirements' section below.

* A marked-up copy of your manuscript that highlights changes made to the original version. You should upload this as a separate file labeled 'Revised Manuscript with Track Changes'.

* An unmarked version of your revised paper without tracked changes. You should upload this as a separate file labeled 'Manuscript'.

If you would like to make changes to your financial disclosure, competing interests statement, or data availability statement, please make these updates within the submission form at the time of resubmission. Guidelines for resubmitting your figure files are available below the reviewer comments at the end of this letter.

As the corresponding author, your ORCID iD is verified in the submission system and will appear in the published article. PLOS supports the use of ORCID, and we encourage all coauthors to register for an ORCID iD and use it as well. Please encourage your coauthors to verify their ORCID iD within the submission system before final acceptance, as unverified ORCID iDs will not appear in the published article. Only the individual author can complete the verification step; PLOS staff cannot verify ORCID iDs on behalf of authors.

We look forward to receiving your revised manuscript.

Kind regards,

Emili Balaguer-Ballester, PhD

Academic Editor

PLOS Computational Biology

Daniele Marinazzo

Section Editor

PLOS Computational Biology

**Additional Editor Comments (if provided):**

**Journal Requirements:**

If the reviewer comments include a recommendation to cite specific previously published works, please review and evaluate these publications to determine whether they are relevant and should be cited. There is no requirement to cite these works unless the editor has indicated otherwise.

1) Your Response to Reviewers should be uploaded as a single, individual file. Please remove your Response to Reviewers from your Cover Letter.

2) Please amend your detailed Financial Disclosure statement. This is published with the article. It must therefore be completed in full sentences and contain the exact wording you wish to be published.

i) State what role the funders took in the study. If the funders had no role in your study, please state: "The funders had no role in study design, data collection and analysis, decision to publish, or preparation of the manuscript."

ii) If any authors received a salary from any of your funders, please state which authors and which funders..

If you did not receive any funding for this study, please simply state: u201cThe authors received no specific funding for this work.u201d

**Reviewers' comments:**

Reviewer's Responses to Questions

**Comments to the Authors:**

**Please note here if the review is uploaded as an attachment.**

Reviewer #1: The authors have satisfactorily addressed all of my comments and questions, as well as those of the other reviewer, and have revised the manuscript accordingly. I recommend the paper for publication in PLOS Computational Biology.

Reviewer #2: The authors have responded to most of my comments. However. I would still require some clarifications.

After the responses about the working point and the noise, now I understand better that the way the models are parametrized for the resting state here seems somewhat different from the way it is done when these neural mass models are employed for whole brain models.

When brain network models are constructed, the initial conditions and noise seeds (the noise is always part of the generative system) are barely of any relevance. I expect this to be the case also for the PSD as a feature for the single node here, given that the initial transient of any model is taken out, the results should be insensitive on the initial conditions and the noise seeds. The only way when the noise can play a role, is if it gets different statistics over time (its strength becomes time-varying as for example in the case of Sip et al, Sci. Adv. 2021).

However, every model is very sensitive on the working point as defined by its parameters, that define its dynamical regime. I think this merits additional discussion. For example the most successful model here, MPR, when used in TVB for the resting state (Lavanga et al. Neuroimage 2023, Fousek et al. Scientific Reports 2024, Breyton et al eLife 2025), it is always in its bistable regime, where the required switching dynamics is driven by the noise and the stimuli from the other regions.

So to be more specific, is the strength of the white noise added to the rs simulations evaluated, and how was it chosen for different models? And second, for one specific model, say MPR, to which dynamical regime do correspond the parameters chosen in the optimization, in respect to the Fig. 1a in Montbrio et al PRX 2015?

**********

**Have the authors made all data and (if applicable) computational code underlying the findings in their manuscript fully available?**

The PLOS Data policy requires authors to make all data and code underlying the findings described in their manuscript fully available without restriction, with rare exception (please refer to the Data Availability Statement in the manuscript PDF file). The data and code should be provided as part of the manuscript or its supporting information, or deposited to a public repository. For example, in addition to summary statistics, the data points behind means, medians and variance measures should be available. If there are restrictions on publicly sharing data or code —e.g. participant privacy or use of data from a third party—those must be specified.

Reviewer #1: Yes

Reviewer #2: **No:** I haven't checked the github repository

**********

PLOS authors have the option to publish the peer review history of their article (what does this mean?). If published, this will include your full peer review and any attached files.

If you choose “no”, your identity will remain anonymous but your review may still be made public.

**Do you want your identity to be public for this peer review?**  For information about this choice, including consent withdrawal, please see our Privacy Policy.

Reviewer #1: No

Reviewer #2: No

[NOTE: If reviewer comments were submitted as an attachment file, they will be attached to this email and accessible via the submission site. Please log into your account, locate the manuscript record, and check for the action link "View Attachments". If this link does not appear, there are no attachment files.]

**Figure resubmission:**

-->While revising your submission, we strongly recommend that you use PLOS’s NAAS tool (https://ngplosjournals.pagemajik.ai/artanalysis) to test your figure files. NAAS can convert your figure files to the TIFF file type and meet basic requirements (such as print size, resolution), or provide you with a report on issues that do not meet our requirements and that NAAS cannot fix.

After uploading your figures to PLOS’s NAAS tool - https://ngplosjournals.pagemajik.ai/artanalysis, NAAS will process the files provided and display the results in the "Uploaded Files" section of the page as the processing is complete. If the uploaded figures meet our requirements (or NAAS is able to fix the files to meet our requirements), the figure will be marked as "fixed" above. If NAAS is unable to fix the files, a red "failed" label will appear above. When NAAS has confirmed that the figure files meet our requirements, please download the file via the download option, and include these NAAS processed figure files when submitting your revised manuscript.

**Reproducibility:**

To enhance the reproducibility of your results, we recommend that authors of applicable studies deposit laboratory protocols in protocols.io, where a protocol can be assigned its own identifier (DOI) such that it can be cited independently in the future. Additionally, PLOS ONE offers an option to publish peer-reviewed clinical study protocols. Read more information on sharing protocols at https://plos.org/protocols?utm_medium=editorial-email&utm_source=authorletters&utm_campaign=protocols

# Author comment: Author response to Decision Letter 2

**DOI:** 10.1371/journal.pcbi.1014222.r004

22 Jul 2026

Attachment Submitted filename: Response_to_Reviewers_auresp_2.pdf

# Peer review: Decision Letter 2

**DOI:** 10.1371/journal.pcbi.1014222.r005

**Authors:** Emili Balaguer-Ballester, Daniele Marinazzo

26 Jul 2026

Dear Ms. Omejc,

We are pleased to inform you that your manuscript 'Neural Population Models for EEG: From Canonical Models to Alternative Model Structures' has been provisionally accepted for publication in PLOS Computational Biology.

Before your manuscript can be formally accepted you will need to complete some formatting changes, which you will receive in a follow up email. A member of our team will be in touch with a set of requests.

Please note that your manuscript will not be scheduled for publication until you have made the required changes, so a swift response is appreciated.

IMPORTANT: The editorial review process is now complete. PLOS will only permit corrections to spelling, formatting or significant scientific errors from this point onwards. Requests for major changes, or any which affect the scientific understanding of your work, will cause delays to the publication date of your manuscript.

Should you, your institution's press office or the journal office choose to press release your paper, you will automatically be opted out of early publication. We ask that you notify us now if you or your institution is planning to press release the article. All press must be co-ordinated with PLOS.

Thank you again for supporting Open Access publishing; we are looking forward to publishing your work in PLOS Computational Biology.

Best regards,

Emili Balaguer-Ballester, PhD

Academic Editor

PLOS Computational Biology

Daniele Marinazzo

Section Editor

PLOS Computational Biology

***********************************************************

Reviewer's Responses to Questions

**Comments to the Authors:**

**Please note here if the review is uploaded as an attachment.**

Reviewer #2: The authors have addressed all my comments and I suggest the paper to be accepted for publication.

As a suggestion, the authors could consider including in the discussion some of the works that used the single population MPR for whole brain modeling, which I mentioned in the previous round. They might be more suitable to illustrate the utility of the single population model, than the currently used reference of 2 populations.

**********

**Have the authors made all data and (if applicable) computational code underlying the findings in their manuscript fully available?**

The PLOS Data policy requires authors to make all data and code underlying the findings described in their manuscript fully available without restriction, with rare exception (please refer to the Data Availability Statement in the manuscript PDF file). The data and code should be provided as part of the manuscript or its supporting information, or deposited to a public repository. For example, in addition to summary statistics, the data points behind means, medians and variance measures should be available. If there are restrictions on publicly sharing data or code —e.g. participant privacy or use of data from a third party—those must be specified.

Reviewer #2: None

**********

PLOS authors have the option to publish the peer review history of their article (what does this mean?). If published, this will include your full peer review and any attached files.

If you choose “no”, your identity will remain anonymous but your review may still be made public.

**Do you want your identity to be public for this peer review?**  For information about this choice, including consent withdrawal, please see our Privacy Policy.

Reviewer #2: No

# Editor report: Acceptance letter

**DOI:** 10.1371/journal.pcbi.1014222.r006

**Authors:** Emili Balaguer-Ballester, Daniele Marinazzo

PCOMPBIOL-D-26-00834R2

Neural Population Models for EEG: From Canonical Models to Alternative Model Structures

Dear Dr Omejc,

I am pleased to inform you that your manuscript has been formally accepted for publication in PLOS Computational Biology. Your manuscript is now with our production department and you will be notified of the publication date in due course.

The corresponding author will soon be receiving a typeset proof for review, to ensure errors have not been introduced during production. Please review the PDF proof of your manuscript carefully, as this is the last chance to correct any errors. Please note that major changes, or those which affect the scientific understanding of the work, will likely cause delays to the publication date of your manuscript.

Soon after your final files are uploaded, unless you have opted out, the early version of your manuscript will be published online. The date of the early version will be your article's publication date. The final article will be published to the same URL, and all versions of the paper will be accessible to readers.

For Research, Software, and Methods articles, you will receive an invoice from PLOS for your publication fee after your manuscript has reached the completed accept phase. If you receive an email requesting payment before acceptance or for any other service, this may be a phishing scheme. Learn how to identify phishing emails and protect your accounts at https://explore.plos.org/phishing.

Thank you again for supporting PLOS Computational Biology and open-access publishing. We are looking forward to publishing your work!

With kind regards,

Kannan R K Kuppusamy, B.TECH BIOTECHNOLOGY

PLOS Computational Biology | Carlyle House, Carlyle Road, Cambridge CB4 3DN | United Kingdom ploscompbiol@plos.org | Phone +44 (0) 1223-442824 | ploscompbiol.org | @PLOSCompBiol
